# Transition metal-catalysed carbene- and nitrene transfer to carbon monoxide and isocyanides

**DOI:** 10.1039/d1cs00305d

**Published:** 2022-06-24

**Authors:** T. R. Roose, D. S. Verdoorn, P. Mampuys, E. Ruijter, B. U. W. Maes, R. V. A. Orru

**Affiliations:** Department of Chemistry and Pharmaceutical Sciences and Amsterdam Institute for Molecules, Medicines & Systems (AIMMS), Vrije Universiteit Amsterdam De Boelelaan 1108 1081 HZ Amsterdam The Netherlands e.ruijter@vu.nl; Organic Chemistry, Aachen-Maastricht Institute for Biobased Materials (AMIBM), Maastricht University Urmonderbaan 22 6167RD Geleen The Netherlands r.orru@maastrichtuniversity.nl; Organic Synthesis Division, Department of Chemistry, University of Antwerp Groenenborgerlaan 171 2020 Antwerp Belgium bert.maes@uantwerpen.be

## Abstract

Transition metal-catalysed carbene- and nitrene transfer to the C1-building blocks carbon monoxide and isocyanides provides heteroallenes (*i.e.* ketenes, isocyanates, ketenimines and carbodiimides). These are versatile and reactive compounds allowing *in situ* transformation towards numerous functional groups and organic compounds, including heterocycles. Both one-pot and tandem processes have been developed providing valuable synthetic methods for the organic chemistry toolbox. This review discusses all known transition metal-catalysed carbene- and nitrene transfer reactions towards carbon monoxide and isocyanides and *in situ* transformation of the heteroallenes hereby obtained, with a special focus on the general mechanistic considerations.

## Abstract 2

In this review the transition metal-catalysed carbene- and nitrene transfer to carbon monoxide and isocyanides will be disclosed. The resulting heteroallene allows for *in situ* transformation towards numerous functional groups and organic compounds.

## Graphical abstract

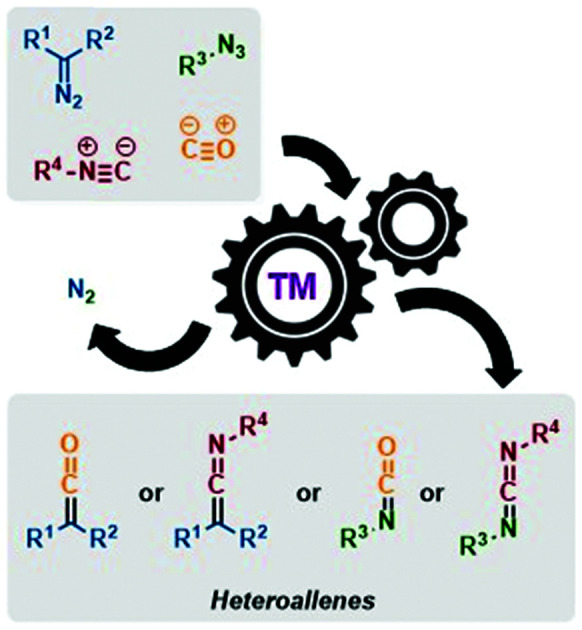

## 1 Introduction

### 1.1. Group transfer to carbon monoxide and isocyanides

**Scheme 1:**
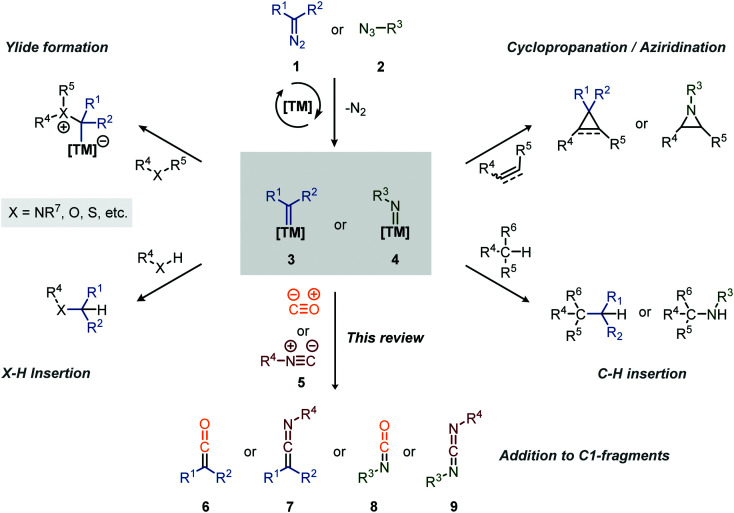
Overview of common transformations involving transition metal-carbene (2) and -nitrene (3) species.

Carbenes and nitrenes are valuable reactive species applied to produce^1^ or (late-stage) functionalise^2^ various complex organic molecules, including numerous medicinally relevant scaffolds and natural products. Transition metal (TM) catalysis plays a pivotal role in the efficient *in situ* formation of these carbene/nitrene species, starting from mainly diazo (or precursors thereof) compounds (1)/azides (2), and in fine-tuning their reactivity.^3,4^ Typical transformations include the reaction with unsaturated bonds to form cyclopropanes^5^ and aziridines,^6^ with heteroatoms to form ylides,^7^ and the insertion into X–H bonds (X = C, N, O, *etc.*, Scheme 1).^8,9^ On the other hand, group transfer of carbenes and nitrenes towards C1-building blocks, such as carbon monoxide (CO) and its isoelectronic counterpart an isocyanide (5) (Scheme 1), surprisingly only received attention from the organic chemistry community in the last two decades. In general, CO is well known for its application in TM-catalysis, not only acting as an important ligand in many catalytically active complexes, but also as C1 insertion partner in carbonylative cross-coupling or addition reactions, providing smooth access to carbonyl derivatives.^10^ Comparably, isocyanides (5) have proven to be valuable C1-building blocks in the construction of imine derivatives *via* TM-catalysed cross-coupling^11^ and the stabilisation of catalytic intermediates in catalysis allowing isolation and characterization.^12^ More generally, they have found widespread use in the fields of synthetic-organic chemistry^13^ in both catalysed and uncatalysed processes, including multicomponent reactions.^14^ Furthermore, both carbonylation^15^ and imidoylation^16^ reactions have shown to be valuable tools towards the synthesis of heterocycles.

The coupling of a carbene or nitrene to CO or an isocyanide provides valuable synthetic intermediates (Scheme 1). More specifically, addition of a carbene to an isocyanide (5) results in a ketenimine (6), which is a versatile reactive species and can participate in *e.g.* nucleophilic- and radical additions, cycloadditions, electrocyclisations and sigmatropic rearangements.^17^ Similarly, the coupling of a nitrene with an isocyanide results in a carbodiimide (9), well known for its application in the synthesis of heterocycles and as peptide coupling agent.^18^ Similarly, the coupling of CO to a carbene and nitrene provides a ketene (6) and isocyanate (8), respectively, both considered valuable synthetic building blocks.^19,20^ Ketenes are for instance involved in the synthesis of β-lactams antibiotics^21^ while isocyanates have found major industrial application in the formation of polyurethanes.^20*b*,22^ The value of the polyurethane market was estimated to be around USD 65.5 billion in 2018.^22^ Therefore, carbene- and nitrene transfer to CO or isocyanides serve as a foothold for the development of novel cascade processes, exploiting *in situ* formed heteroallene 6–9 as building blocks. This review will highlight such transformations.

### 1.2. General mechanistic considerations

**Scheme 2:**
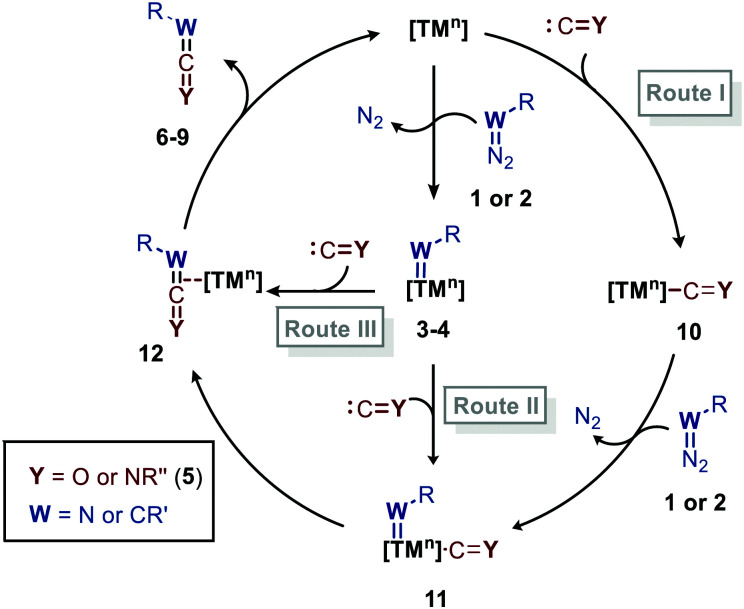
General catalytic cycle for the carbene/nitrene transfer to CO and RNCs.

Before highlighting the recent developments in carbene and nitrene group transfer reactions to carbon monoxide and isocyanides affording heteroallenes 6–9, we will first discuss in this section a general mechanistic rationale based on the available literature. Our analysis is summarized in the cycle depicted in Scheme 2. The general catalytic cycle shows multiple potential pathways.

**Scheme 3:**
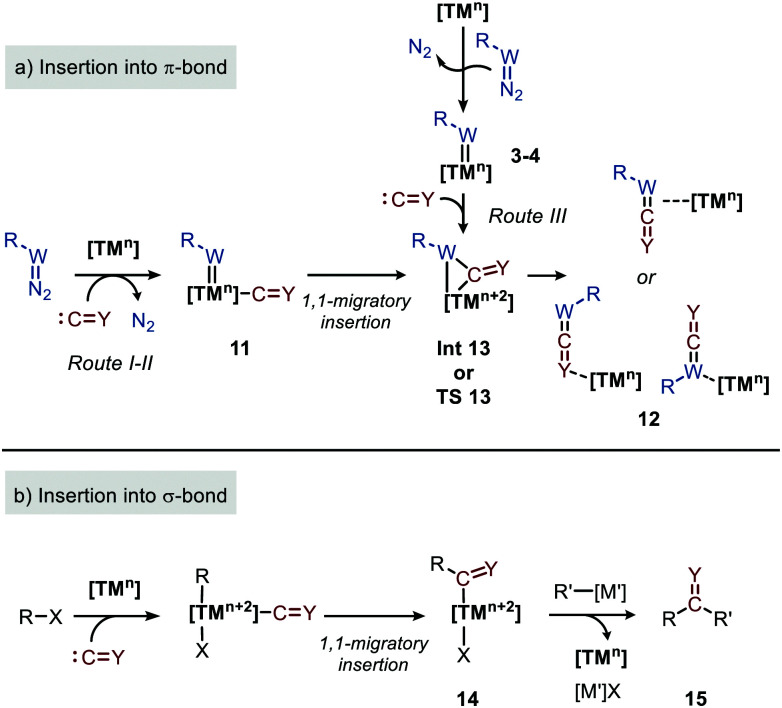
1,1-Migratory insertion of species C = Y (Y = O, NR′′ (5)) into: (a) TM = W (W = CR′ (3), N (4)) double bonds and; (b) TM–R single bonds.

As both CO and isocyanides (5) readily coordinate to TMs,^23,24^ route I commences with the formation of the corresponding CO or isocyanide coordinated complex 10. Important to note is that although CO and isocyanides are isoelectronic the latter are less π-accepting and more sterically hindered affecting TM complexation. Subsequent reaction of diazo compounds and azides with 10 results in the carbene/nitrene coordinated complex 11. Another possibility is the reverse, *i.e.* formation of the carbene/nitrene complex 3–4 prior to coordination of the C1-fragment, also providing intermediate 11 (route II). In general, TM–carbene and TM–nitrene species are accessed *via* the corresponding diazo-type compounds (1) or azides (2), respectively, involving extrusion of molecular nitrogen. However, other carbene and nitrene precursors can also be applied, although less frequently encountered in the field of group transfer to CO and isocyanides. Subsequently, formal 1,1-migratory insertion in complex 11 results in the corresponding η^2^-coordinated metallacycle 13 as shown in Scheme 3a. However, intermediate 13 may alternatively also serve as transition state in certain catalytic systems^25^ towards metallated heteroallene 12, of which several coordination modes are depicted.^26^ The migratory insertion step in intermediate 11 to 13 can be considered as a formal insertion of either CO^3*c*^ or isocyanide^11*c*,23^ into a TM = CRR′ or TM = NR π-bond. Carbonylative and imidoylative transformations relying on the formal 1,1-migratory insertion of CO or isocyanide into a TM-C σ-bond also exists,^10,11,27^ forming TM-acyl/imidoyl species 14 (Scheme 3b). Both route I and II in Scheme 2 rely on 1,1-migratory insertion *via* the transfer of metal bound C = Y species towards the carbene/nitrene moiety in complex 11. Alternatively, non-metal bound species C = Y can be transferred to the coordinated carbene/nitrene moiety *via* an outer sphere addition to complex 3–4 (route III in Scheme 2).^4*a*,28^ In all routes I–III, intermediate 13 or TS 13 (Scheme 3a) is involved. However, there are also other less common possibilities and these will be highlighted when applicable in the specific section of this review. From intermediate **13** or *via* TS **13**, complex 12 can be accessed, which consists of linear intermediate 6–9 coordinated to the TM centre. These heteroallenes can be transformed *in situ* with *e.g.* nucleophiles while coordinated to, or after dissociation of, the metal centre. The operative mechanism (route I, II or II) depends on both the type of transfer reaction and the TM catalyst used, as well as on the nature and number of additional ligands on the TM center.

### 1.3. Organisation of the review

The reactions will be categorized based on the heteroallene formed, *i.e.* ketene (6), ketenimine (7), isocyanate (8), or carbodiimide (9). First the carbene transfer to CO (Section 2.1) and isocyanides (Section 2.2) will be discussed followed by the nitrene transfer to CO (Section 3.1) and isocyanides (Section 3.2). In each section the formation of the heteroallene is subdivided according to the transition metal catalyst used. In order to demonstrate the synthetic utility of these valuable linear intermediates, all known examples where these are formed and transformed *in situ* have been included. For every transformation several of the reported examples have been selected and shown in the respective scheme to create an awareness of the reader for the scope. The synthesis of several medicinally relevant compounds will also be highlighted when relevant. The suggested reaction mechanism of the processes is discussed referring to the general proposed cycle in Scheme 2. Substrates in multicomponent transformations will be colour coded to account for the origin of the atoms in the resulting product.

## 2 Transition-metal catalysed carbonylation and imidoylation of carbenes

In this section, the carbene transfer to carbon monoxide and isocyanides (5) will be discussed. Transformations proceeding through linear heteroallene intermediates 6 and 7 (Scheme 1) will be categorized according to the transition metal that was used in heteroallene formation. Mechanistic discrepancies with regard to the general catalytic cycle of Scheme 2 will be highlighted.

### 2.1. Carbene transfer to carbon monoxide

Ketenes are considered valuable synthetic building blocks, *e.g.* for the construction of β-lactams *via* [2+2]-cycloaddition with imines.^19*b*^ These are typically accessed *via* Wolff rearrangement of α-diazo ketones or base-mediated elimination of HCl from α-acidic acyl chlorides. However, the TM-catalysed carbene transfer to CO is a valuable alternative for the formation of intermediates 6. As this topic has been reviewed in 2011 by Wang *et al.*,^29^ we will focus on examples reported after 2010. Nonetheless, mechanistically relevant earlier examples will still be briefly mentioned.

#### Cobalt

**Scheme 4:**
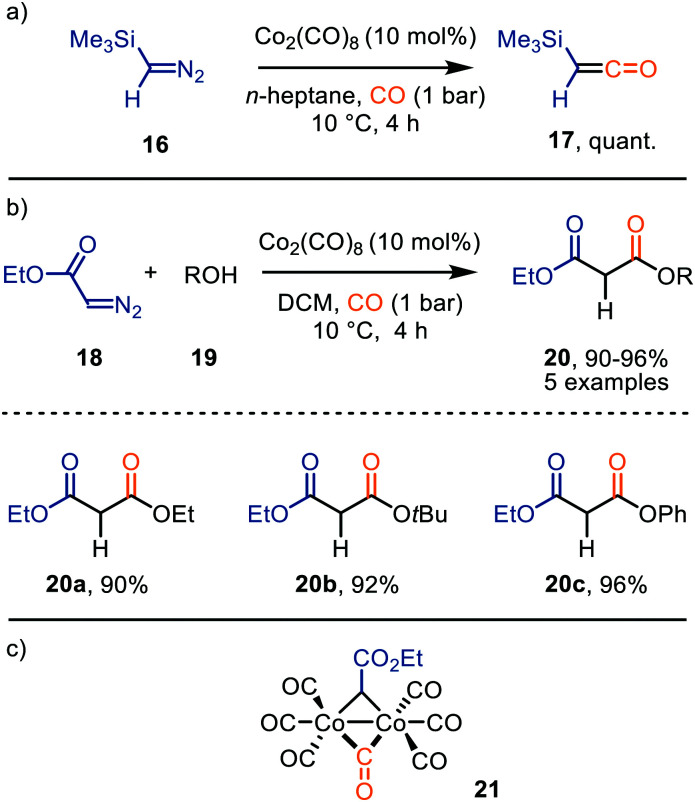
Co^0^-Catalysed synthesis of (a) trimethylsilylketene 1**7**, and (b) malonate derivatives 20. (c) μ^2^-Co–carbene intermediate 21.

The use of Co_2_(CO)_8_ as base-metal catalyst for the carbonylation of carbenes was nicely demonstrated by Ungváry *et al.* (Scheme 4). The synthesis of trimethylsilylketene 17 from trimethylsilyldiazomethane 16 proceeded in quantitative yield (Scheme 4a).^30^ Subsequently, the authors report the formation of malonate derivatives 20 with the same catalyst from α-diazo acetate 18 and anhydrous alcohols 19 (Scheme 4b).^31^ The reaction towards malonate derivatives 20 tolerates linear primary and tertiary aliphatic as well as aromatic alcohols to give the corresponding products in near quantitative yield (90–96%). Detailed mechanistic studies indicate that ketene formation proceeds *via* a 1,1-migratory insertion step from bridged Co–carbene intermediate 21 (Scheme 4c).^32^ Considering the nature of this catalyst, as the CO fragment is already coordinated to the metal centre, the mechanism follows general route I starting from intermediate 10 onwards in Scheme 2.

**Scheme 5:**
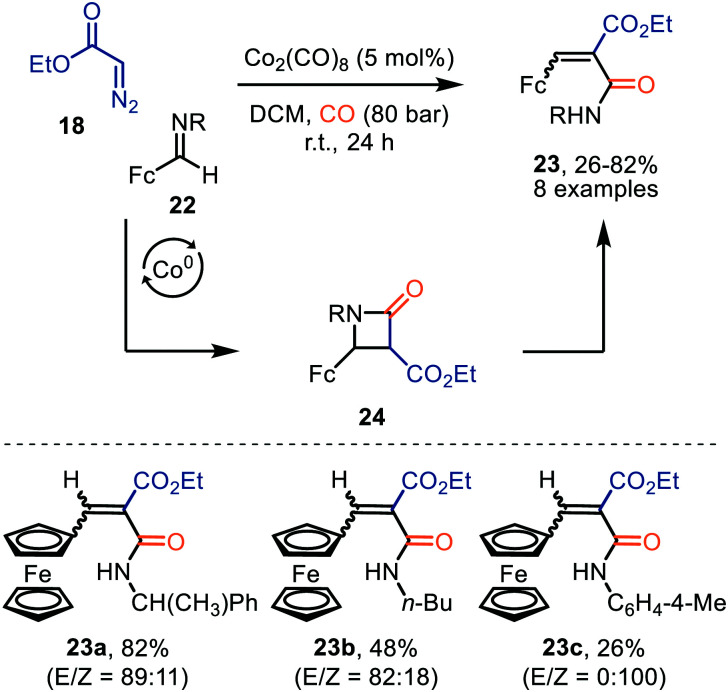
Co^0^-Catalysed synthesis of ferrocenyl-α,β-unsaturated amides 23. Fc = bis(η^5^-cyclopentadienyl)iron.

Co_2_(CO)_8_ was also employed for the formation of ferrocenyl α,β-unsaturated amides 23 from ethyl diazoacetate (18) and ferrocenyl imine 21, under high CO pressure (Scheme 5).^33^ After CO-catalysed carbonylation of 18, a [2+2]-cycloaddition occurs between the obtained ketene and imine 22, generating β-lactam intermediate 24. The authors highlight that these intermediates 24 are unstable and undergo β-lactam ring cleavage to α,β-unsaturated amides 23, which were isolated in low to good yields (26–82%). There is precedent in literature that β-lactams rearrange towards the corresponding amide 23.^34^ The transformation tolerates a variety of primary, secondary, tertiary alkyl, and activated aryl substituents on the imine nitrogen.

**Scheme 6:**
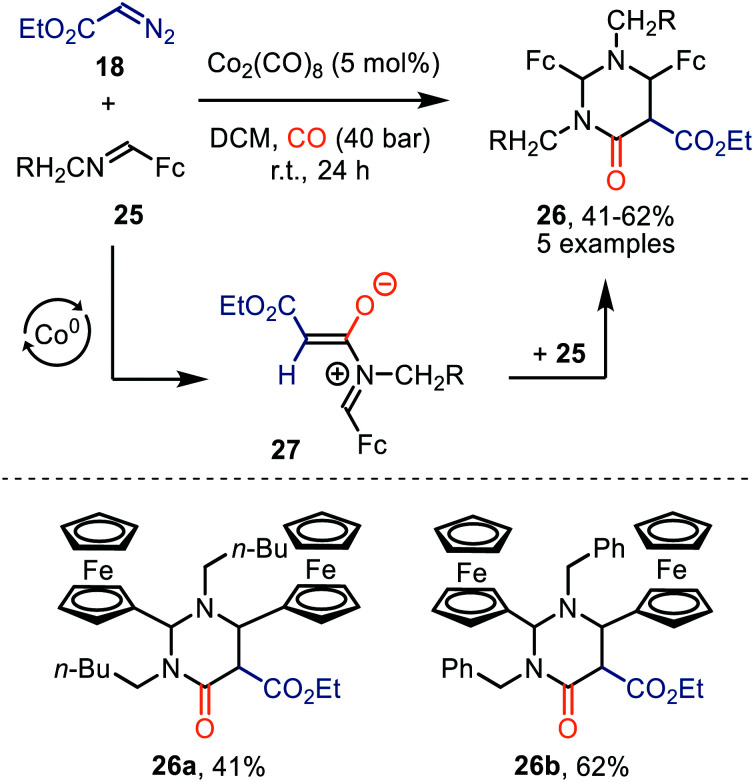
Co^0^-Catalysed formation of tetrahydro-4(1*H*)-pyrimidinones (26). Fc = bis(η^5^-cyclopentadienyl)iron.

Interestingly, when a *N*-methylene moiety is present in ferrocenyl imine 25 tetrahydro-4(1*H*)-pyrimidinones 26 are formed as alternative products (Scheme 6).^35*a*^*N*-Methylene-ferrocenylimines 25 (R = alkyl, aryl) were tolerated and products 26 were furnished in moderate yields (41–62%). The formation of the ketenes involved is consistent with our general mechanistic cycle *via* route I (Scheme 2). The reactive species that play a key role in the process are zwitterionic intermediates 27. Subsequent formal [4+2]-cycloaddition with a second imine 25 affords heterocyclic scaffold 26. Yields were not optimal as the authors add the diazo ester (**18**) and imine 25 in an equimolar amount.^35*b*^

**Scheme 7:**
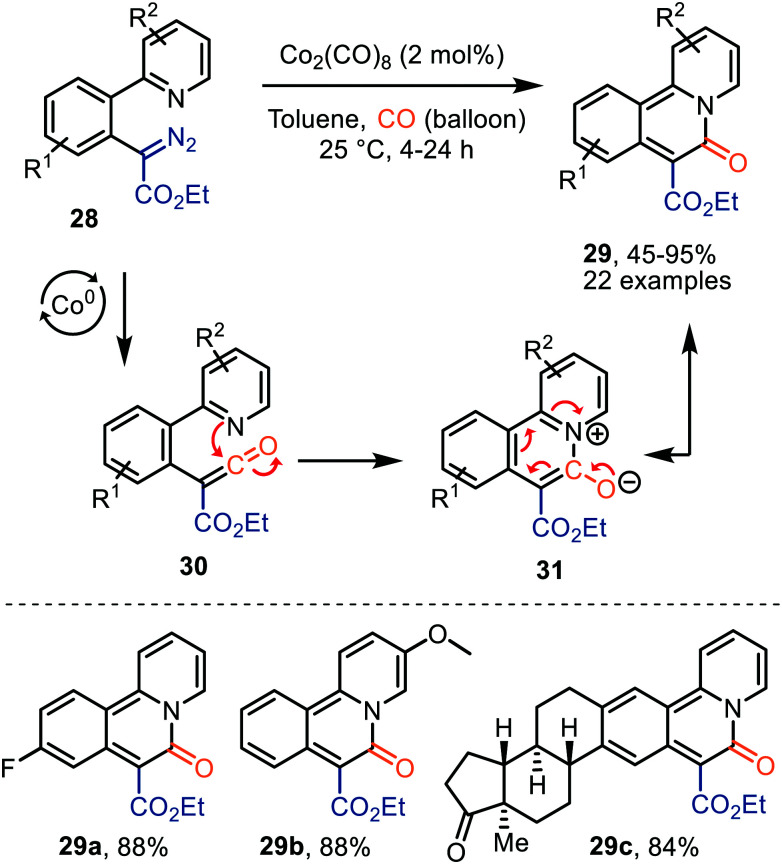
Co^0^-Catalysed formation of benzoannulated pyridin-2(1*H*)-one derivatives 29.

Lee *et al.* demonstrated the use of Co_2_(CO)_8_ as catalyst in the formation of pyridoisoquinolinones (29) *via* ketene intermediate 30 (Scheme 7).^36^ The mechanism complies with route I of our postulated mechanism in Scheme 2, based on the nature of the catalyst. The authors propose that subsequent cyclization of the ketene group with the pyridinyl moiety in 30 provides a zwitterionic pyridinium enolate (31), which is in resonance with the product 29. The presence of both electron-donating and electron-withdrawing -substituents in several positions of α-aryl-α-diazoacetates (R^1^ and R^2^) 28, indicates an overall high functional group tolerance for this carbonylative cyclization.

**Scheme 8:**
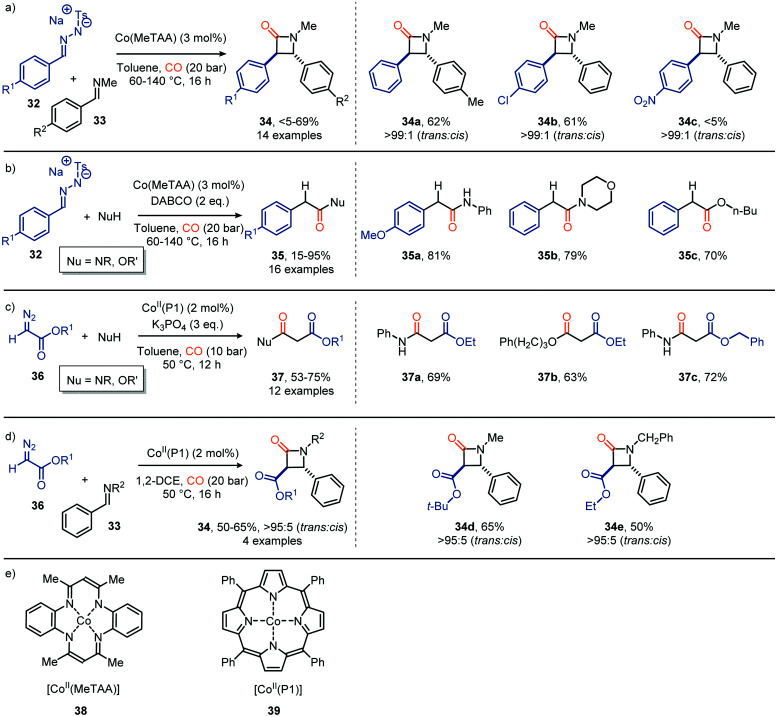
Synthesis of (a) β-lactams 34 with [Co(MeTAA)], (b) acyl derivatives 35 with [Co(MeTAA)], (c) malonate derivatives 37 with [Co(P1)], (d) β-lactams 34 with [Co(P1)]. (e) Structure of [Co(MeTAA)] (38) and [Co(P1)] (39).

Besides Co_2_(CO)_8_ other Co-based catalysts have also been reported to achieve the carbonylation of carbenes staring from diazo compounds and *N*-tosylhydrazones as carbene precursor (Scheme 8). For example, de Bruin *et al.* developed a catalytic route towards β-lactams 34 (Scheme 8a) and acyl derivatives 35 (Scheme 8b) based on Co^II^ tetramethyltetraaza-[1,4]-annulene (Co(MeTAA), 38, Scheme 8e).^37^ Their strategy produces β-lactams in a diastereoselective manner *via* a formal [2+2] cycloaddition of the corresponding *in situ* generated ketene with benzaldehyde imine 33 (Scheme 8a). Electron-donating or halogen substituents (R^1^) in the *para*-position of tosylhydrazones 32 as carbene precursor delivered the desired product in moderate yield (33–62%), whereas electron-withdrawing groups were less tolerated. In contrast, electron-withdrawing moieties on the aryl ring of imine 33 (R^2^) did not hamper the transformation and delivered the desired β-lactams in moderate yield (36–69%). In addition, the ketene formed from 32 using the same Co^II^ catalyst 38 are compatible with a wide variety of nucleophiles, such as anilines, aliphatic acyclic and cyclic amines primary-, secondary-, and tertiary- aliphatic alcohols, to furnish the corresponding amides and esters 35 (Scheme 8b). Employing catalyst 39, featuring a related tetra dentate nitrogen ligand, and α-diazo acetate 36 as carbene precursor and nitrogen nucleophiles afforded malonate derivatives 37 (Scheme 8c).^38^ The porphyrin-based catalytic system was able to accommodate a broad range of primary aliphatic and aromatic amines, morpholine and a primary alcohol. Next, catalyst 39 was also used in the formation of β-lactams 34, using diazo ester 36 as the carbene precursor (Scheme 8d).^35^ Decent yields were obtained with high *trans* diastereoselectivities (*trans* : *cis* = >95 : 5). In addition to the use of α-diazo acetate 36, in both transformations in Scheme 8c and d, *N*-tosylhydrazones also proved suitable carbene precursors in this transformation (not shown).^38^

**Scheme 9:**
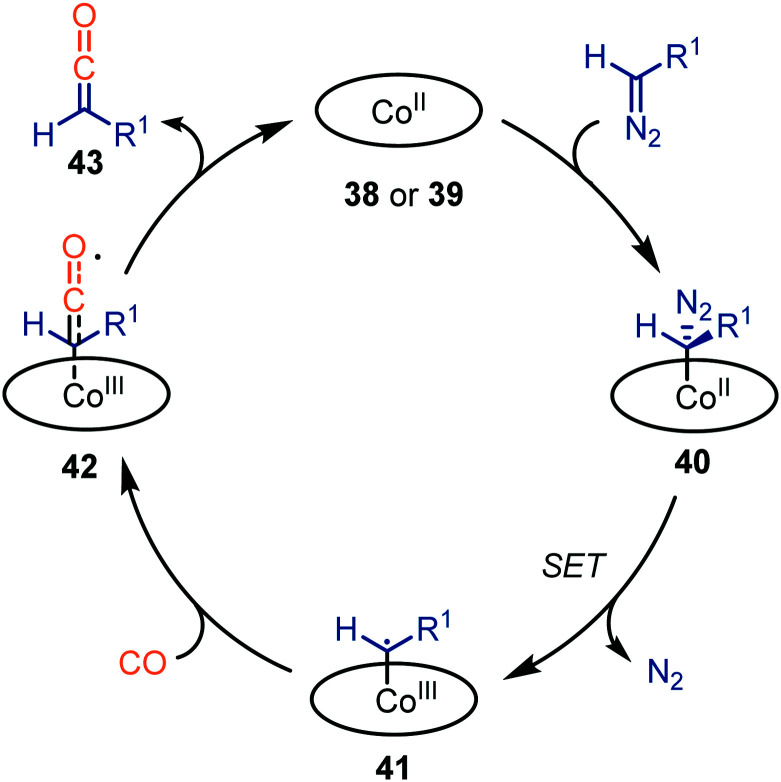
General catalytic cycle for the carbene transfer reaction to CO with Co^II^-catalysts 38 & 39.

The authors propose a mechanism, which accounts for both catalysts 38 and 39, supported by experimental work and DFT calculations (Scheme 9).^37,38^ Initially, the diazo compound coordinates to the Co^II^-centre, resulting in complex 40. Subsequent extrusion of N_2_*via* SET results in the Co^III^–carbene radical 41. Next, CO adds to carbene radical complex 41*via* an outer sphere mechanism, forming species 42. Notably, dissociation of the ketene with the [Co^II^(MeTAA)] complex 38 is less facile than with [Co^II^(P1)] complex 39. In case of catalyst 38, Co^III^-species 42 can be considered as a short-lived intermediate whereas with porphyrin catalyst 39, species 42 is found to be a transition state. Subsequently, the liberated ketene 43 can be transformed *in situ*. The mechanism complies with route III of our general proposed catalytic cycle (Scheme 2).

#### Rhodium

**Fig. 1:**
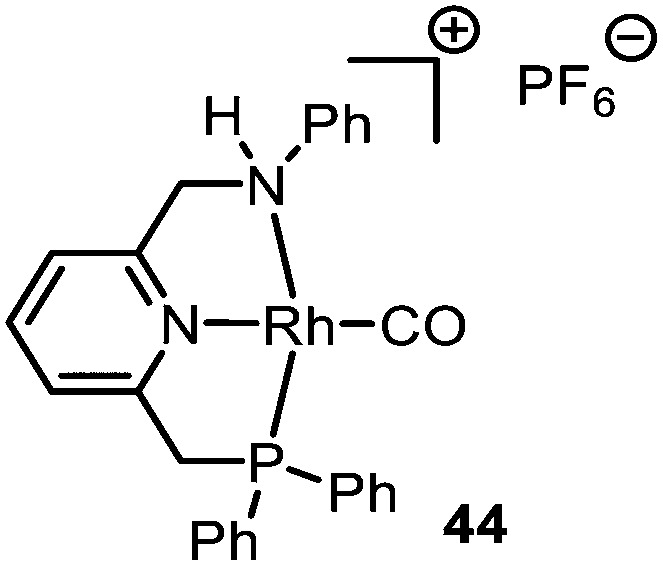
Rh^I^-PNN PF_6_ complex 44.

**Scheme 10:**
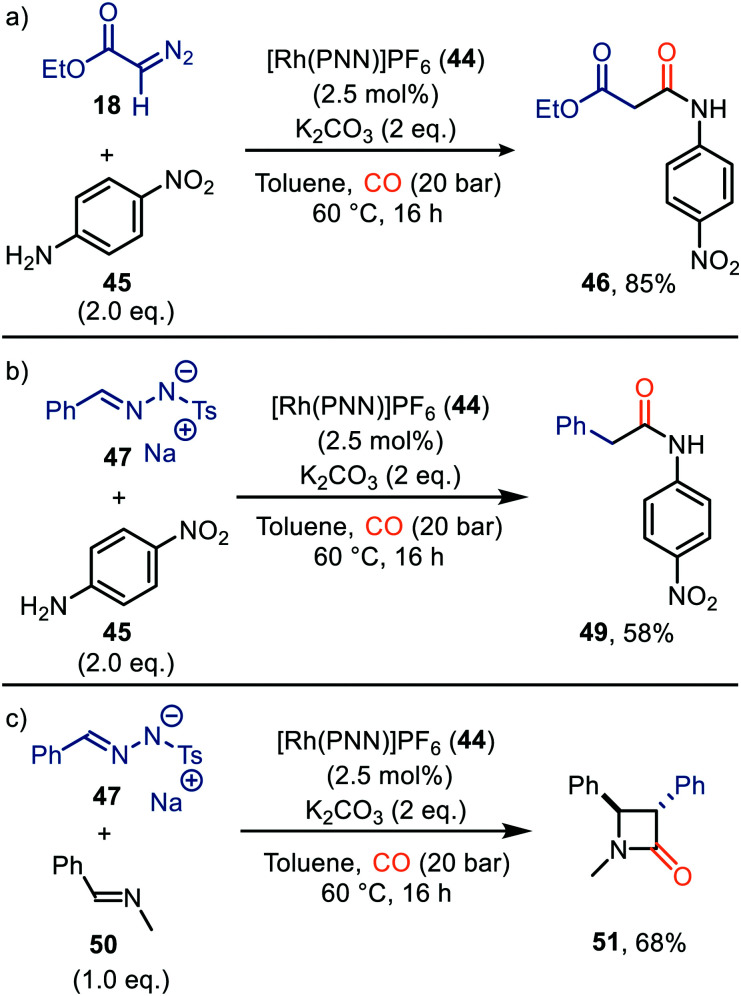
Formation of (a) malonate monoamide derivate 46, (b) amide 49, and (c) β-lactam 51 carbonylation of carbenes catalysed by Rh-PNN PF_6_ complex 44.

Rhodium was also found to be a suitable metal for the carbonylation of carbenes. De Bruin *et al.* have demonstrated the applicability of cationic Rh^I^-PNN pincer complex 44 (Fig. 1) for the synthesis of α-amido ester 46 (Scheme 10a), amide 49 (Scheme 10b) or β-lactam 51 (Scheme 10c).^39^ Multiple PNN ligands were evaluated, however, catalyst 44 proved to be superior. The *in situ* formed ketenes react either with 4-nitroaniline (45) (Scheme 10a and b) or *N*-benzylidenemethanamine 50 (Scheme 10c). A more detailed substrate scope of this carbonylation was not addressed by the authors. Notably, a direct *N*-H bond insertion of the carbene with 4-nitroaniline (45) was also observed as side product. The mechanism of ketene formation follows the general mechanism in Scheme 2. Although DFT calculations suggest route III is favoured, proceeding *via* an outer-sphere mechanism, routes I and II could not be fully excluded. In this catalytic system, the formation of the metal-carbenoid species is the rate determining step. The η^2^-intermediate species (13, Scheme 3a) is described by the authors as a Rh^III^-η^2^-CC_ketene_ species, which can be accessed upon 1,1-migratory insertion (route I, Scheme 2) or external attack of CO (route III, Scheme 2).

**Scheme 11:**
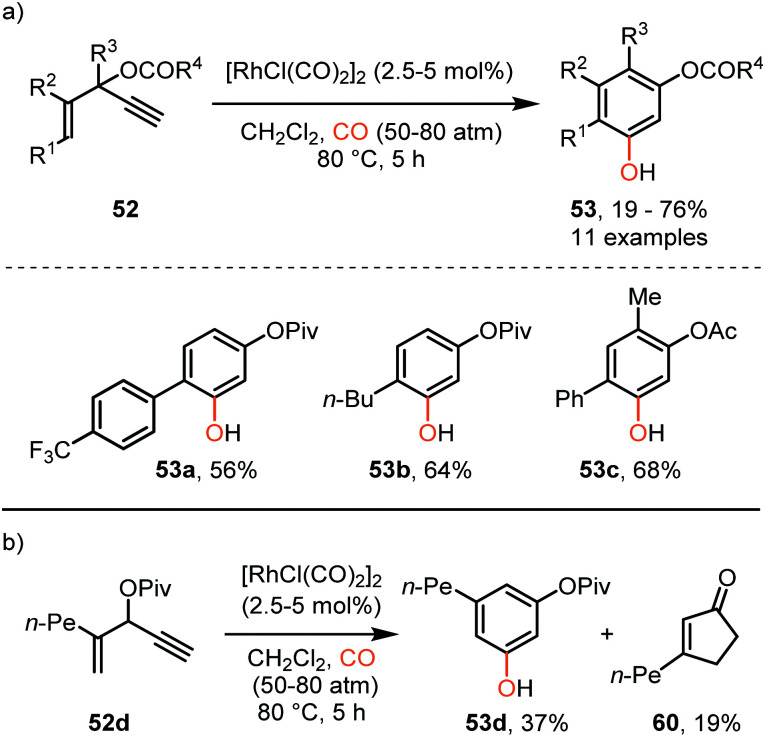
(a) Rh^I^-Catalysed [5+1] cycloaddition of 3-acyloxy-1,4-enynes (52) with CO. (b) Side reaction that occurs with terminal alkene 52d forming product 60.

So far, we discussed only reactions involving carbene sources derived from diazo compounds or deprotonated *N*-tosylhydrazones. However, unsaturated systems can also be efficient precursors. Malacria and co-workers employed 3-acyloxy-1,4-enynes 52 as carbene precursor. An electrophilic Rh^I^-complex was able to convert 52 and CO to a wide range of mono esterified resorcinol derivatives 53 (Scheme 11a).^40^ Various R^1^-substituents, such as electron-rich- and electron-poor aromatic groups as well as linear-, and branched aliphatic groups, are accepted and deliver the target resorcinols in poor to moderate yield (19–76%). In addition, primary aliphatic substituents can be decorated on both position R^2^ and R^3^ of product 53. Interestingly, for terminal enyne 52d a non-carbonylated cyclopentenone 60 is in competition with the desired resorcinol derivative (Scheme 11b).

**Scheme 12:**
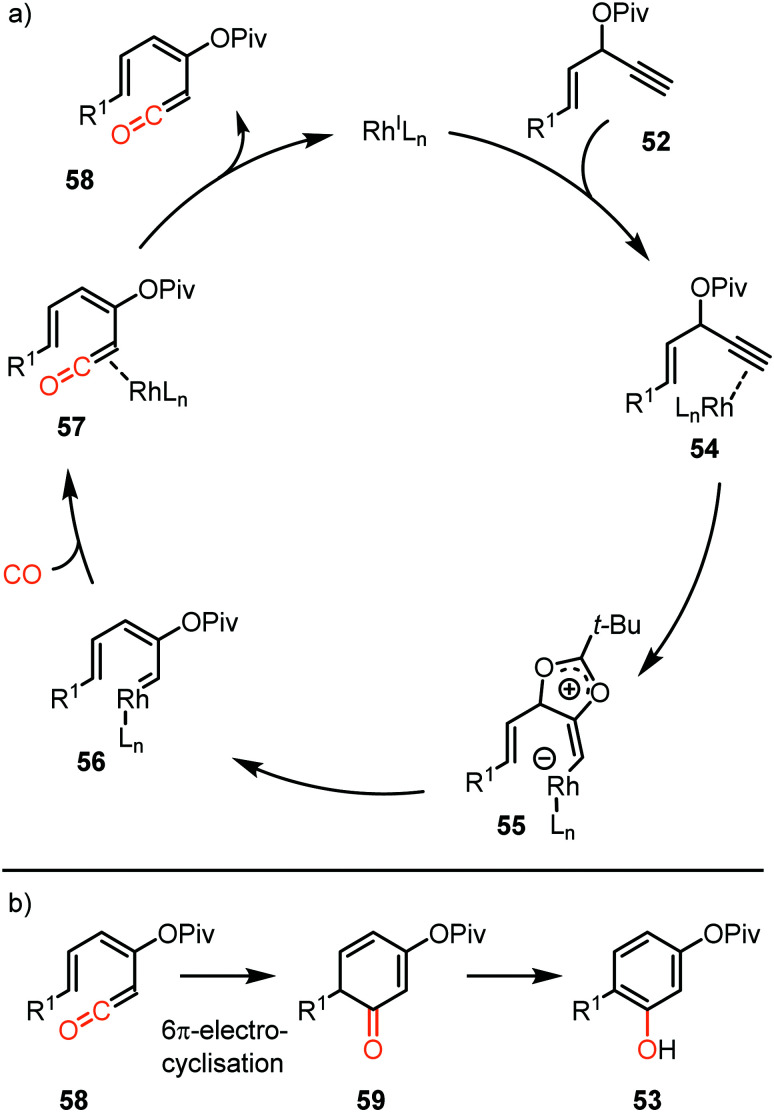
(a) The mechanism of formation of ketene 58 with carbene precursor 52. (b) Electrocyclisation of ketene 58.

The proposed mechanistic cycle (Scheme 12a)^41^ is initiated by electrophilic activation of the alkyne moiety, leading to an η^2^ π-complex 54. Intramolecular nucleophilic attack from the carboxylate on the activated triple bond affords zwitterionic Rh-complex 55. Subsequent 1,2-acyloxy migration generates Rh-carbenoid 56, which in turn undergoes 1,1-migratory insertion with carbon monoxide, finally resulting in Rh-ketene complex 57. After dissociation from the metal, ketene 58 participates in a 6π-electrocyclization, providing 59 (Scheme 12b), which undergoes spontaneous keto-enol tautomerisation towards aromatic phenol derivative 53. An important note is that cyclopentenone **60** is produced when the enyne ester has an internal alkyne moiety. This causes the reaction to proceed *via* a 1,3- rather than 1,2-acyloxy migration.

**Scheme 13:**
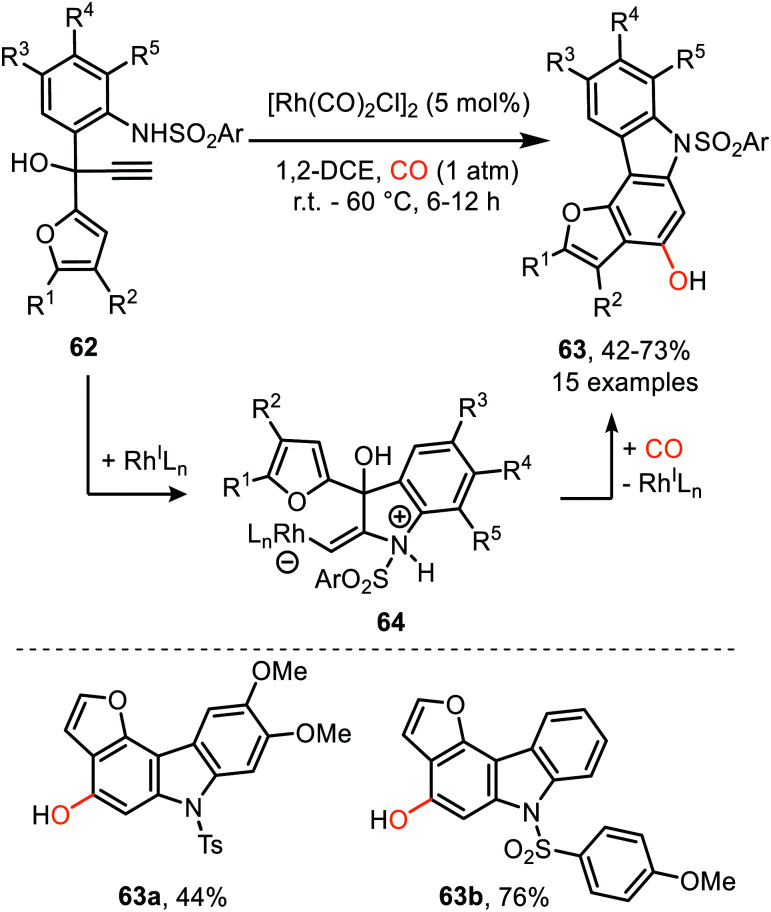
Rh^I^-Catalysed carbene transfer to CO employing 62 as carbene precursor followed by electrocyclisation of ketene.

Similarly, Tang *et al.* described a Rh^I^-catalysed process transforming propargylic alcohol 62 into furo annulated carbazole derivatives 63 (Scheme 13).^42^ The sulfonamide moiety performs well with both electron-donating and -withdrawing aryl groups. In addition, the furane and aniline ring could be decorated with a variety of substituents. Furthermore, the furan moiety can be replaced by a pyrrole or thiophene scaffold. The proposed mechanism of carbene formation is similar to the representation in Scheme 12. The crucial difference is the nucleophilic attack of the sulfonamide substituent on the metal activated alkyne of 62 thereby generating zwitterionic Rh-complex 64, rather than the carboxylate attack in 54 (Scheme 12a) leading to zwitterionic Rh-complex 55. Elimination of water in complex 64, Scheme 13, provides Rh carbenoid which upon CO insertion yields ketene. Metal decomplexation and 6π electrocyclisation followed by tautomerisation delivers target compounds 63.

#### Palladium

**Scheme 14:**
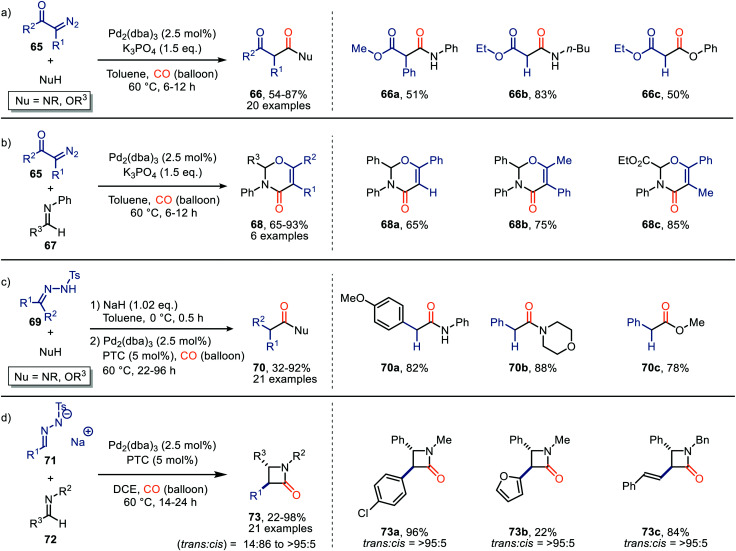
Pd-Catalysed carbonylation of carbenes forming: (a) malonate derivatives 66, (b) 2,3-dihydro-4*H*-1,3-oxazin-4-one derivatives 68, (c) acylated species 70, (d) β-lactams 73. PTC = Aliquat 336 or trioctylmethylammonium chloride.

As one of the most chemically diverse and explored noble metals, Pd also proved to be an efficient catalyst in the carbonylation of carbenes. Wang *et al.* described multiple Pd-catalysed carbonylation reactions based on α-diazo compounds 65 which *in situ* generate ketenes (Scheme 14a and b).^43^ The authors demonstrate the synthesis of malonate derivatives 66 both from substituted α-diazo ketones and α-diazo esters 65 (Scheme 14a). The formed ketene could be trapped by a range of nucleophiles, such as aniline derivatives, primary, and secondary aliphatic amines, and phenol derivatives. In addition, the ketene could also be trapped with imine 67 in a [4+2] cycloaddition to furnish 2,3-dihydro-4*H*-1,3-oxazin-4-ones 68 in good yield (65–93%) (Scheme 14b).

To further investigate the reaction of ketenes, generated by a Pd-catalysed carbonylation, the authors focused on *N*-tosylhydrazones (69), which are *in situ* deprotonated, as carbene precursor (Scheme 14c).^43^ With respect to the *N*-tosylhydrazone (69) the transformation tolerates aromatic substituents containing electron-donating, and electron-withdrawing groups, biaryl-, and alkyl substituents (R^1^ and R^2^). Primary, and secondary amines, phenol, and primary alcohols all furnished as nucleophiles providing the corresponding carbonyl compound 70 in moderate to good yields (32–92%). The authors also investigated the use of *N*-tosylhydrazone salts 71 under slightly modified conditions. Ketene formed reacts *in situ* with imine 72*via* [2+2]-cycloaddition reaction delivering the corresponding β-lactams 73 in moderate to good yield (Scheme 14d).^43^ Typically, a high preference for the *trans*-isomer was observed. Especially when α,β-unsaturated *N*-tosylhydrazones were employed (*trans* : *cis* = >95 : 5). The authors suggest a Pd-assisted isomerisation pathway, which allowed for the interconversion of the *cis*-isomer to the more stable *trans*-isomer of β-lactam 73. The provided mechanism is based on precedents in literature and DFT calculations. Furthermore, the authors suggest ketene formation to proceed *via* 1,1-migratory insertion where the Pd–CO complex is formed prior to formation of the Pd-carbenoid, which is in accordance with route I in our general proposed mechanism (Scheme 2).

Although several transition metals can catalyse the carbonylation of carbenes, as described in this Section 2.1, the provided examples are still rather limited. As ketenes can undergo a variety of transformations and are reported as intermediates in numerous syntheses we believe that further research into its alternative synthesis from metal carbenoids and carbon monoxide will allow for the development of novel cascade processes towards a much broader range of high-value products. While the main focus is still on noble metals, cobalt has been relatively well explored, though successful use of other base metals has not been disclosed. There seems to be a resemblance with other carbonylation reactions here where Co complexes are also common catalysts.^44^ Generally Co_2_CO_8_ is the most common cobalt source and the C1-building block is actually already present in the *in situ* formed catalytic active species without external carbon monoxide supply. Due to the nature of the catalyst the mechanism commences with carbene formation, therefore, reactions catalysed by these complexes are categorized under route I (Scheme 2). So, the nature of the catalyst is important to determine the actual mechanistic pathway *via* which the group transfer proceeds. The same holds for complexes with multidentate ligands such as Co-porphyrin complexes (Scheme 8), where there is no more vacant site to allow for *cis* coordination of the two fragments and, therefore, the carbene transfer to carbon monoxide in this case has to proceed *via* an outer sphere mechanism (route III, Scheme 2). Transition metals in low oxidation state with a lot of d-electrons such as Pd^0^ will form strong complexes with carbon monoxide *via* back bonding and therefore favor route I. Clearly, the type of transition metal (early, mid or late) and its oxidation state are at play here. Considering carbon monoxide is a gas the pressure will obviously also play a role in the discrimination between route I and II.

### 2.2. Carbene transfer to isocyanides

The carbene transfer to isocyanides affords a ketenimine (7) (Scheme 1) and was already discovered in 1919 by Staudinger.^45^ Multiple synthetic strategies towards ketenimines have been developed, of which the Wittig reaction with isocyanates is most commonly reported.^17*a*^ Ketenimines display versatile reactivity, in for example nucleophilic and radical additions, cycloadditions and sigmatropic rearrangements. They have shown to be valuable building blocks for the construction of *N*-enriched heterocycles, such as indole-, (iso)quinoline- or carbazole-like scaffolds.^17*b*^ In this section we highlight the formation and *in situ* transformation of ketenimines, accessed *via* the TM-catalysed carbene transfer to isocyanides.

#### Cobalt

**Scheme 15:**
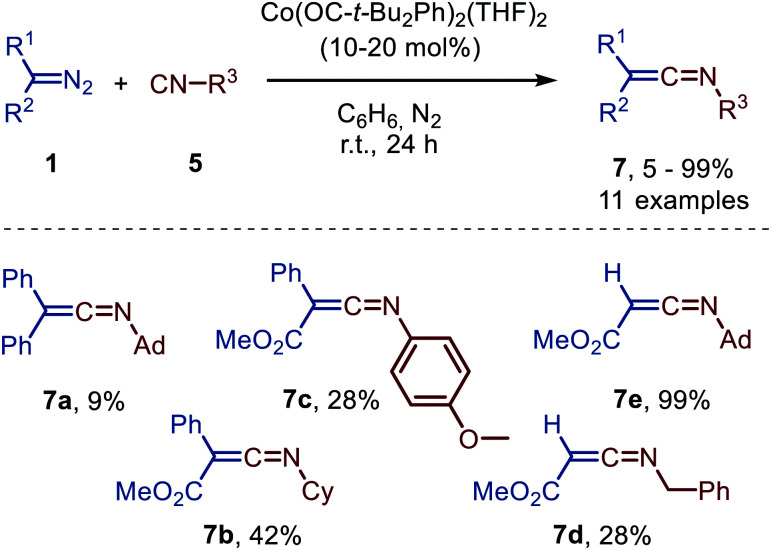
Ketenimine 7 formation from diazo compounds (1) and isocyanides (5) catalysed by a bis-alkoxy Co^II^-complex.

As observed for the carbonylation of carbenes, cobalt is also an efficient catalyst to achieve the carbene transfer to isocyanides (5). For example, Groysman *et al.* demonstrated that a high-valent bis-alkoxy Co^II^-complex catalyses ketenimine (7) formation from diazo compounds (1) and primary, secondary, tertiary or aromatic isocyanides (Scheme 15).^46^

Unfortunately, poor yields were obtained, when donor-donor carbenes were employed (R^1^ = R^2^ = Ph) and the corresponding ketenimine 7a could only be obtained efficiently using stoichiometric amounts of Co. Donor-acceptor (R^1^ = Ph, R^2^ = CO_2_Me) and acceptor (R^1^ = H, R^2^ = CO_2_Me) carbene precursor smoothly reacted to afford ketenimine 7b/7c and 7d/7e, respectively. The postulated mechanism is based on literature precedents, experimental data, and DFT calculations and is in accordance with route II of the general mechanism (Scheme 2).

**Scheme 16:**
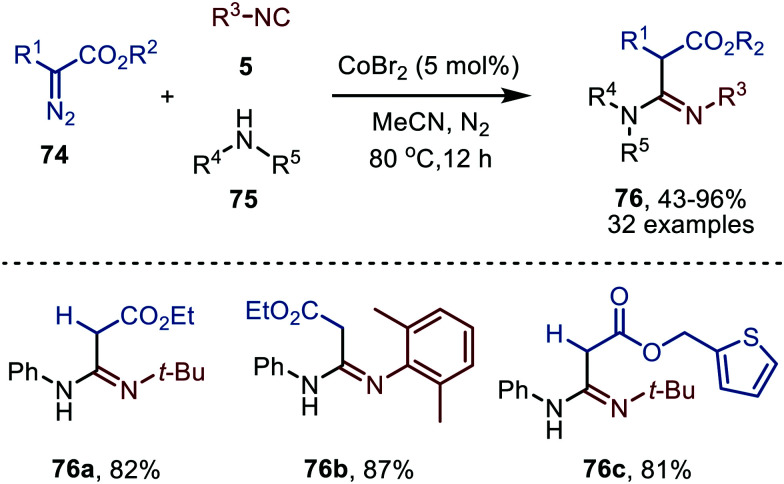
Co^II^-Catalysed synthesis of amidines 76 from α-diazo esters (74), isocyanides (5) and amines (75).

In 2021 a Co^II^Br_2_-catalysed carbene transfer to isocyanides was reported, employing α-diazo esters 74 as carbene precursors (Scheme 16).^47^ This three-component reaction employs amines (75), which capture the ketenimine intermediate to immediately afford amidines 76 in moderate to excellent yield. Notable is that the reaction tolerates tertiary aliphatic isocyanides and aromatic isocyanides as exemplified by amidines 76a/c and 76b, respectively (Scheme 16).

**Scheme 17:**
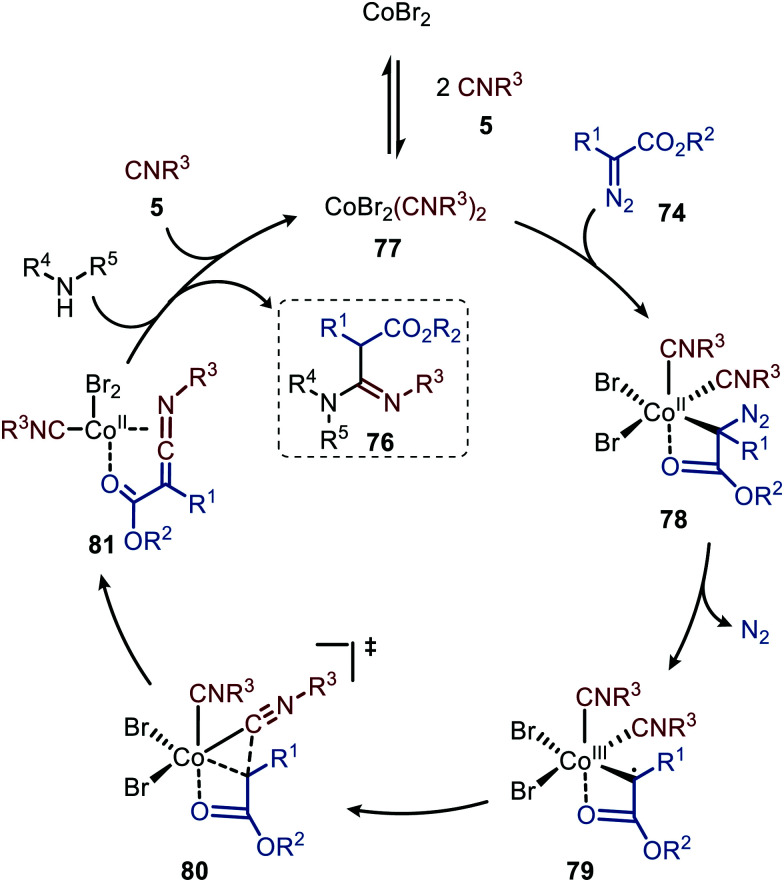
Proposed catalytic cycle of the CoBr_2_-catalysed carbene transfer to isocyanides (5) *via* a Co^III^–carbene radical intermediate 79 followed by trapping of ketenimine 81 with amine.

The authors propose a tentative mechanism, which proceeds *via* Co^III^-radical species 79, accessed from complex 78 after nitrogen extrusion (Scheme 17).^47^ Radical trapping experiments in the presence of TEMPO support this. The authors suggest that isocyanide complex 77 is formed prior to carbene formation and subsequent migratory insertion, which is in line with general route I of Scheme 2. Control experiments with the isolated ketenimine highlighted that CoBr_2_ has a dual catalytic role. Besides facilitating the carbene transfer, it also acts as Lewis acid in the activation of the ketenimine in intermediate 81 (Scheme 17).

#### Nickel

**Scheme 18:**
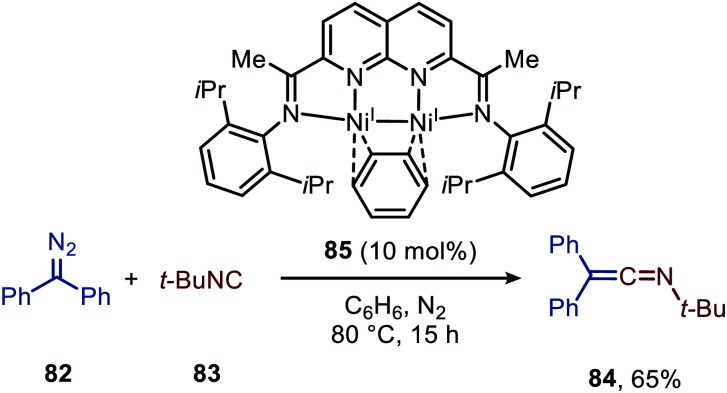
Ketenimine (84) formation from diazodiphenylmethane (82) *t*-butylisocyanide (83) utilising di-nickel complex 85 as catalyst.

The use of nickel in the carbene transfer to isocyanides was demonstrated by Uyeda *et al.*^48*a*^ Catalytic application of Ni was feasible using di-nickel(i) complex^48*b*^85 in the formation of ketenimine 84 from *t*-BuNC (83) and diazodiphenylmethane (82) (Scheme 18). However, despite this catalytic example they predominantly discuss the stoichiometric formation of ketenimine 84, using complex 85. No extensive optimisation of their catalytic system is reported, and the scope and limitations of the reaction were not studied. The proposed mechanistic pathway is in agreement with route II in Scheme 2. This is based on stoichiometric experiments with the corresponding isolated dinuclear Ni_2_(μ-CPh_2_)-complex followed by addition of the isocyanide. The authors state that for catalytic turn over, the isocyanide should be added dropwise over a long period of time. Direct addition of isocyanide 83 results in demetallation and formation of inactive Ni^0^(*t*-BuNC)_4_. In general, only a handful of examples are found in literature that describe base-metal catalysed carbene transfer to isocyanides, especially in comparison to the analogous base-metal catalysed carbonylation of carbenes. Therefore, there is plenty of room for further developments within this contemporary area.

#### Rhodium

**Scheme 19:**
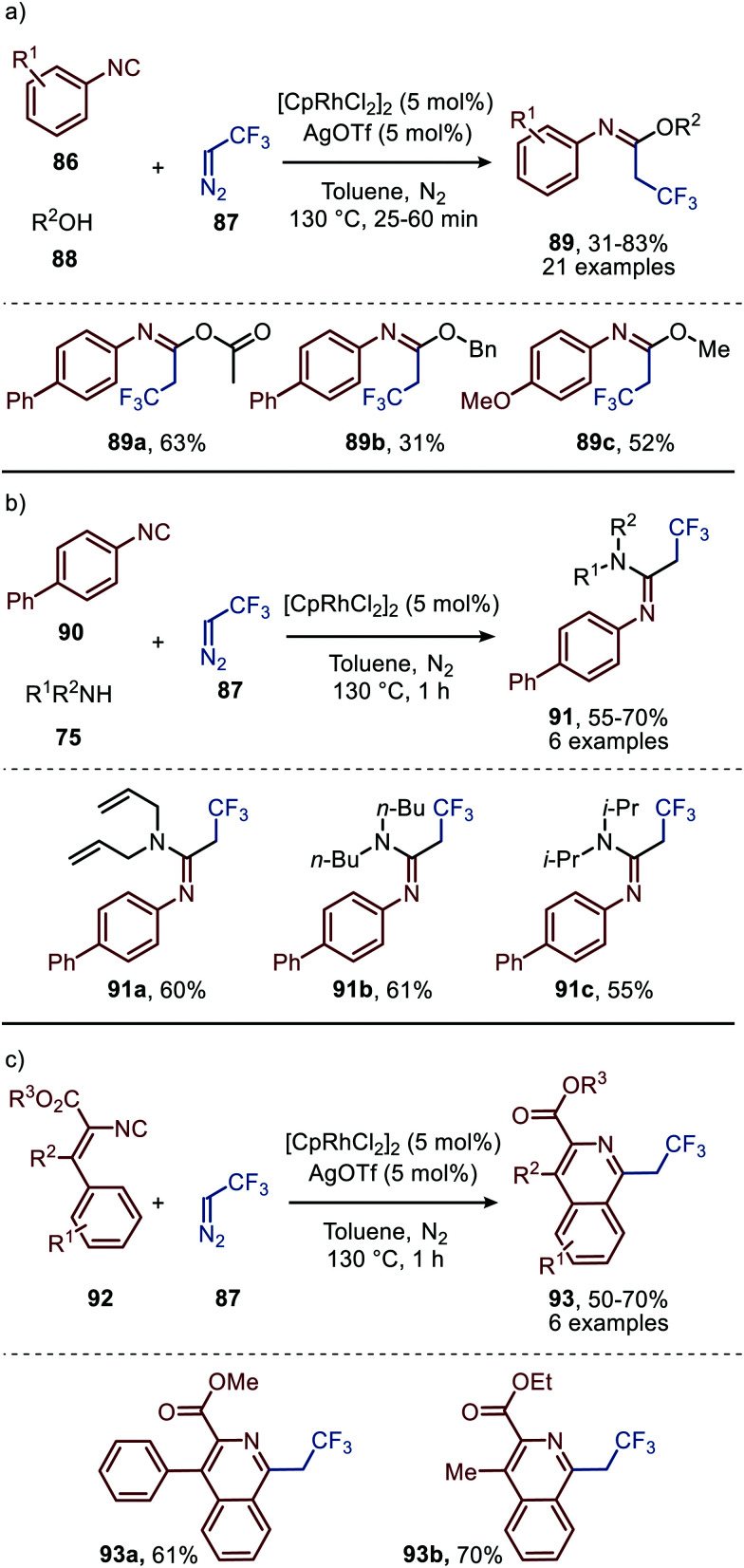
Rh^I^-Catalysed formation of (a) imidates (89) (b) amidines (91) and (c) isoquinolines (93).

Similarly, to the carbonylations discussed in Section 2.1, noble metals have been a major focus of research for the imidoylation of carbenes. For example, Zhao *et al.* reported multiple Rh^I^-catalysed carbene transfers based on trifluorodiazoethane (87) (Scheme 19a–c).^49^ First the authors present a three-component reaction towards imidates (89) using aromatic isocyanides (86) and alcohols (88) (Scheme 19a). The AgOTf was added as co-catalyst facilitating nucleophilic attack on the *in situ* formed ketenimine. Substituted aromatic isocyanides bearing electron-donating or electron-withdrawing groups are generally accepted. Noteworthy is that the reaction with aliphatic isocyanides did not afford the corresponding imidates (89). In addition, the reaction tolerates a wide variety of primary and secondary alcohols furnishing the corresponding imidates 89 in moderate to good yields (31–83%). The alcohols can be decorated with linear, branched, and cyclic alkyl-, allyl-, and benzyl- as well as electron-rich and electron-deficient aryl groups (R^2^). Acetate groups also proved to be effective in yielding the desired product 89a in good yield (Scheme 19a).

**Scheme 20:**
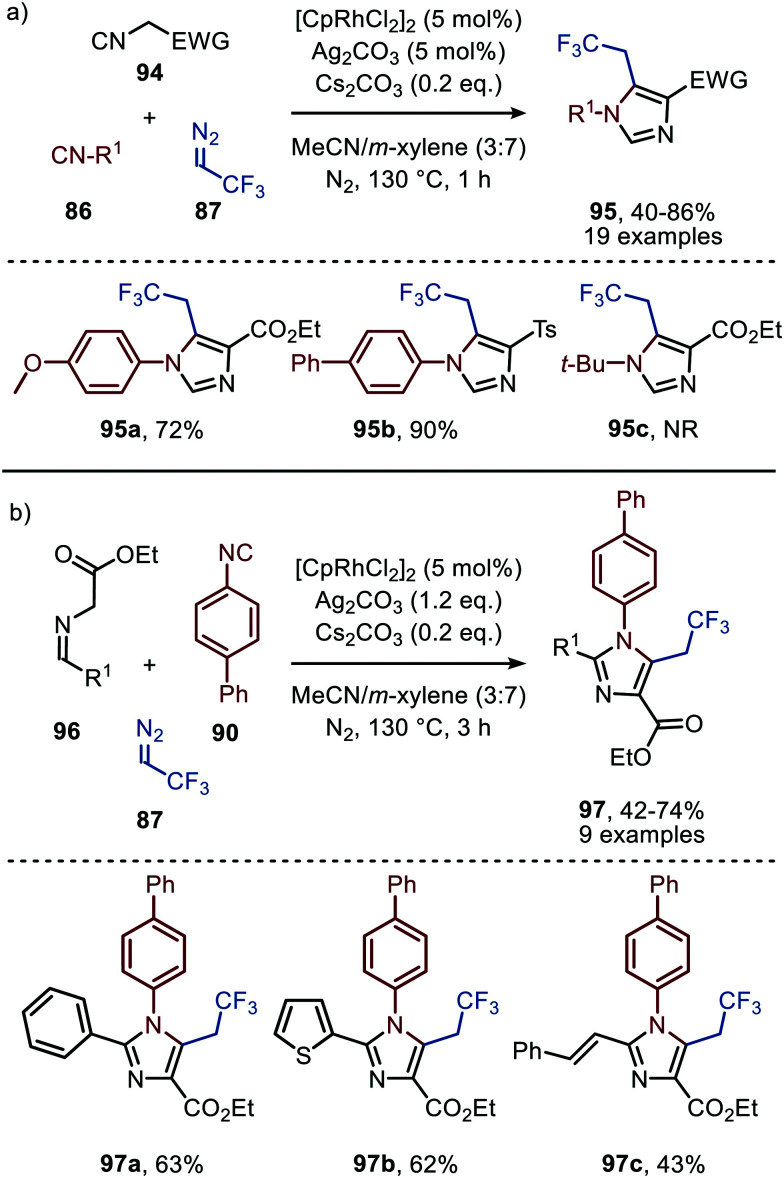
Rh^I^-Catalysed synthesis of trifluoroethyl-substituted imidazoles 95 & 97 based on α-acidic (a) isocyanides (94) or (b) imines (96). NR = no reaction.

The applicability of this tandem reaction was extended towards secondary aliphatic nitrogen nucleophiles (75), affording a variety of amidines 91 in moderate to good yield (Scheme 19b), some with functional handles that allow for follow-up chemistry *e.g.* a cyclisation (91a, Scheme 19b). No silvertriflate was required due to the increased nucleophilicity of amines compared to alcohols. Furthermore, the authors demonstrate the use of 2-phenylethenyl isocyanide 92 for the synthesis of 2-trifluoroethylisoquinolines (93) (Scheme 19c).^49^ The reaction proceeds *via* a AgOTf-catalysed intramolecular electrocyclisation of the arene and the corresponding ketenimine, affording heterocycle 93. The proposed mechanism involves isocyanide coordination prior to carbene formation, which follows route I in our general mechanism (Scheme 2). In continuation of this research the same group reported two additional Rh^I^-catalysed three-component reactions towards other heterocycles (Scheme 20a and b), in 2019.^50^ The reaction from aryl isocyanides (86), 2,2,2-trifluorodiazoethane (87), and α-acidic isocyanides (94) afforded trifluoroethyl-substituted imidazoles 95 in moderate to good yields (Scheme 20a).

**Scheme 21:**
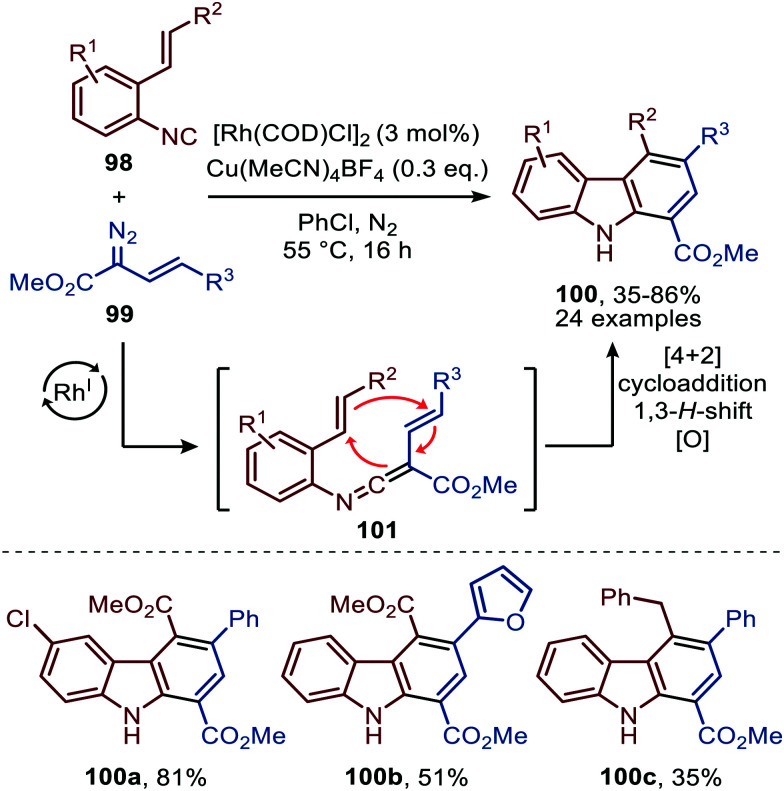
Rh^I^-Catalysed carbene transfer of 99 to isocyanide 98 followed by [4+2]-cycloaddition, 1,3-H shift and oxidation on *in situ* generated ketenimine 101.

The reaction proceeds *via* selective ketenimine formation between aromatic isocyanide 86 and carbene precursor 87, followed by an addition of the activated α-acidic isocyanide 94 on the ketenimine intermediate under basic conditions. The authors state that Ag_2_CO_3_ acts as a Lewis acid in the addition. After a 5-endo-dig cyclization and 1,3-*H* shift imidazoles 95 were obtained. This three-component reaction tolerates a wide variety of aromatic isocyanides (86) exceptionally well, but vinyl isocyanides also delivered the desired trifluoroethyl-substituted imidazoles. Unfortunately, the scope of the reaction could not be extended towards aliphatic isocyanides, such as *t*-BuNC (83) as illustrated for compound 95c. Similarly, imidazole scaffolds 97 were obtained from the ketenimine intermediate, formed from aromatic isocyanide 90 and 2,2,2-trifluorodiazoethane (87), by reaction with azomethine ylide generated from α-acidic imine 96 with base (Scheme 20b). The cyclisation was followed by a 1,3-*H* shift and oxidative rearomatization with silver carbonate, rationalising it cannot be used catalytically here. The transformation is compatible with a wide variety of R^1^ substituents on imine 96. These groups include electron-rich and electron poor aryls, 1-naphthyl, heteroaryls and alkenes (Scheme 20b). The ketenimine formation presumably proceeds *via* route I as described in Scheme 2. In related work Zhao *et al.* describe a Rh^I^-catalysed carbene transfer to *ortho*-alkenylaryl isocyanides 98 which is *in situ* transformed into carbazole derivatives 100 (Scheme 21).^51^ The cascade reaction has a high functional group compatibility (*e.g.* esters (*e.g.*100a/b), amides, (hetero)aryl (*e.g.*100b), and benzyl moieties (*e.g.*100c)), including a broad range of substituents on alkenyldiazoacetates 99, with different electronic characteristics, were tolerated in the reaction.

**Scheme 22:**
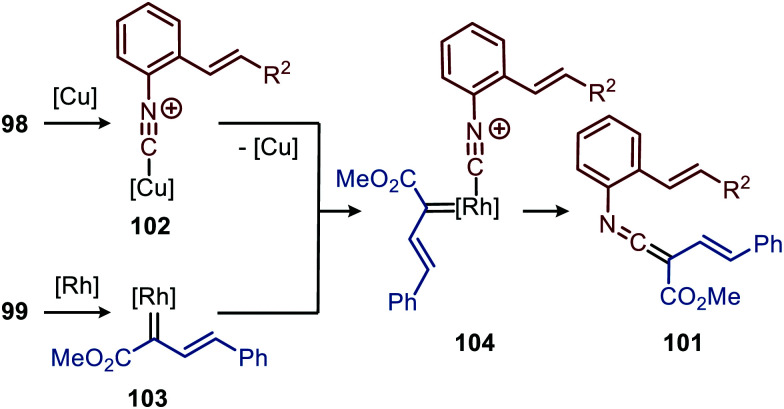
Rh^I^-Catalysed ketenimine 101 formation, employing *in situ* formed Cu^I^-complex 102 as isocyanide reservoir.

The authors show in their optimisation that the Cu^I^ salt co-catalyst is a crucial additive in order to obtain reasonable yields of carbazoles 100. They propose two plausible roles of the copper(i) additive.^51^ First the authors postulate that the Cu^I^ salt could aid in the aerobic oxidation towards the desired carbazole 100. Second is that the Cu^I^ species is actively involved in the proposed mechanism as isocyanide reservoir (Scheme 22). As Cu^I^ has a high affinity for isocyanides, it could facilitate the transfers of isocyanide 98 towards Rh-carbenoid complex 103, *via* coordination complex 102, generating Rh^I^-carbenoid 104. Subsequent 1,1-migratory insertion results in the desired ketenimine 101. A sequential intramolecular [4+2] cycloaddition, 1,3-H shift, and oxidation finally results in the carbazole reaction product 100 (Scheme 21).

#### Palladium

**Scheme 23:**
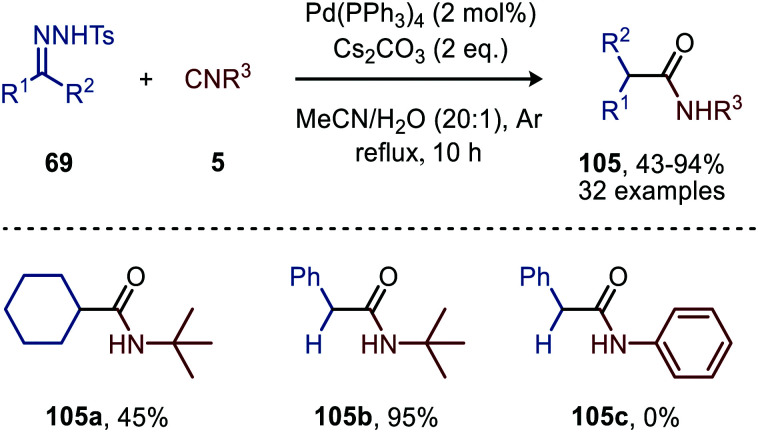
Pd^0^-Catalysed formation of amides (105) *via* a three-component reaction involving *N*-tosylhydrazones (69), isocyanides (5) and water.

As stated before, this noble metal is widely employed in especially homogeneous catalysis. Not surprisingly, Pd has also frequently been reported to catalyse the carbene transfer to isocyanides. In 2011, Cai *et al.*^52^ described the first Pd^0^-catalysed carbene transfer to isocyanides (5), using *N*-tosylhydrazones (69) as carbene precursor (Scheme 23). For the deprotonation of 69 Cs_2_CO_3_ is used. The authors demonstrated that in the presence of water acting as nucleophile, the *in situ* formed ketenimine could be converted into amides 105 in moderate to excellent yields.

Aryl substituted *N*-tosylhydrazones (69) afforded amides 105 without any problem, however (bis)-alkyl *N*-tosylhydrazones required a higher reflux temperature and therefore 1,4-dioxane : H_2_O (20 : 1) was used as solvent mixture to obtain a decent yield of the corresponding amide 105a (Scheme 23). With regard to the isocyanide scope, primary-, secondary- and tertiary aliphatic isocyanides are accepted (105b, Scheme 23). Aromatic isocyanides on the other hand did not lead to any product formation (105c, Scheme 23). The authors postulate a mechanism, which is in accordance with route I of our general mechanism (Scheme 2).

**Scheme 24:**
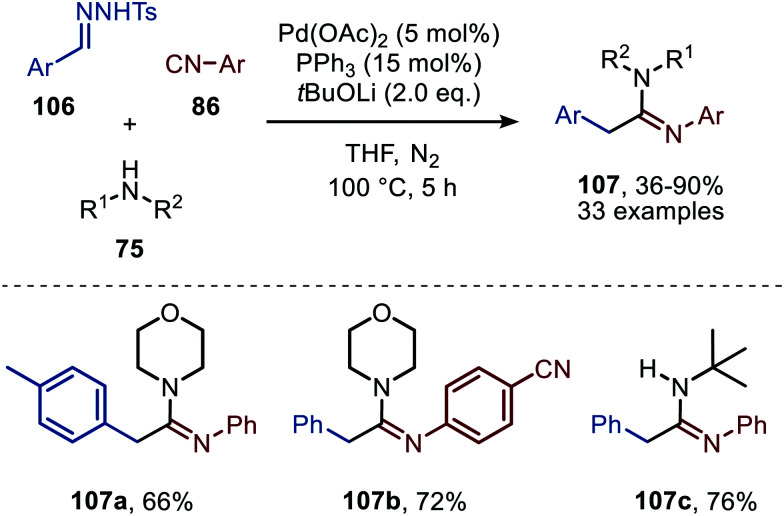
Pd-Catalysed formation of amidines (107) *via* a three-component reaction involving isocyanides (86), *N*-tosylhydrazones (106) and amines (75).

Consecutively, Cheng and co-workers describe a Pd-catalysed imidoylation of carbenes, using amines rather than water to trap the ketenimine *in situ* (Scheme 24).^53^ The formation of amidines 107 proceeds in overall reasonable to good yields from benzaldehyde *N*-tosylhydrazones (106), aromatic isocyanides (86), and amines (75). In this case *t*BuOLi was applied as base. The substrate scope with respect to the substituents on the aromatic ring of the benzaldehyde *N*-tosylhydrazones 106 is quite broad. In addition, diverse substituted aromatic isocyanides with both electron-donating and electron-withdrawing groups can be employed. Primary, secondary, and tertiary aliphatic isocyanides were not accepted in this reaction. Additionally, aliphatic primary, secondary, and secondary cyclic amines (75) are tolerated in this transformation. The authors propose a mechanism based on some control experiments, which is in compliance with route I of our general mechanism (Scheme 2). However, the oxidation state of the Pd catalyst involved is not specified by the authors.

**Scheme 25:**
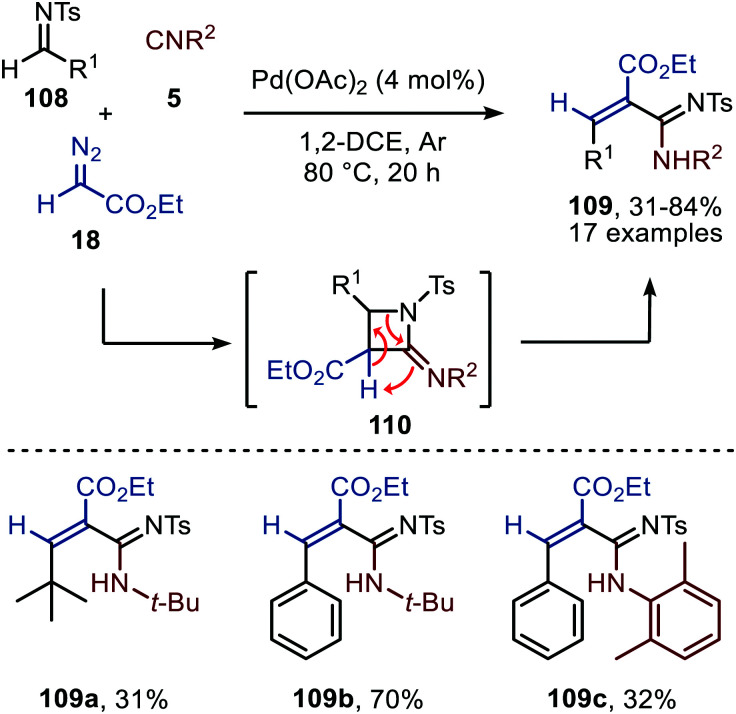
Pd(OAc)_2_-Catalysed three-component reaction of *N*-tosylimines (108), ethyl diazoacetate (18), and isocyanides (5) towards acrylamidines 109.

Cai and co-workers introduce a Pd(OAc)_2_-catalysed three-component reaction involving an *in situ* ketenimine formation followed by a [2+2]-cycloaddition with *N*-tosylimine 108 (Scheme 25).^54^ Interestingly, the expected four-membered 2-iminoazetidines (110) were not detected and undergo a ring-opening under the given reaction conditions, providing acrylamidines 109. Under the optimised conditions, a broad range of benzaldehyde *N*-tosylimines (R^1^ = aryl) are tolerated, resulting mainly in the *E*-acrylamidine product 109. The authors also provide an example of a tertiary alkyl *N*-tosylimine to afford alkenecarboximidamide 109a, albeit in low yield (Scheme 25). As for the isocyanide scope, the reaction with secondary and tertiary isocyanides afforded high yields, whereas aromatic isocyanides performed less in the reaction as exemplified for structure 109c. Also in this study the oxidation state of the Pd-intermediates involved is not specified.

**Scheme 26:**
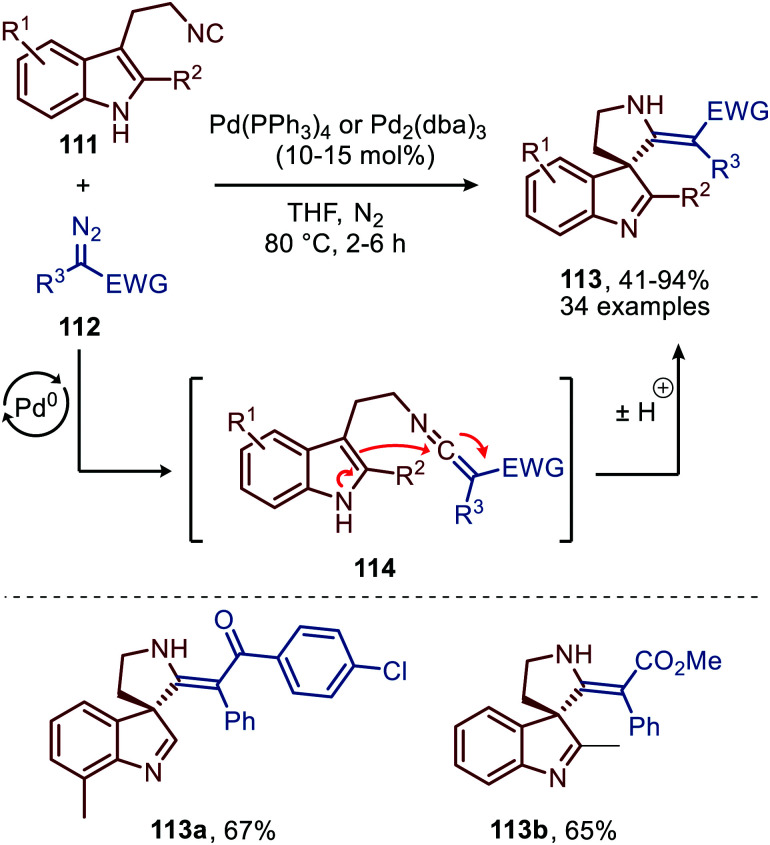
Synthesis of spiroindolenines 113*via* spirocylisation of *in situ* generated ketenimine 114 under Pd^0^-catalysis.

Liu and co-workers demonstrated that 3-(2-isocyanoethyl)indoles (111) are suitable reactants to synthesise complex spiroindolenines 113 (Scheme 26).^55^ These isocyanides 111 react with α-diazo-esters or ketones (112) under Pd^0^ catalysis to afford the corresponding ketenimine intermediate 114. Subsequent intramolecular nucleophilic attack of the indole C3-position on the ketenimine carbon in a 5-*endo*-dig fashion results in the target spiroindolenines 113 in moderate to excellent yield. Substituents were well tolerated on the benzene ring and C2-position of the indole moiety of tryptamine-derived isocyanide 111. The α-diazo scope 112 investigated was predominantly of the donor-acceptor type, bearing a substituted aromatic ring at the α-position (R^3^) (Scheme 26).

**Scheme 27:**
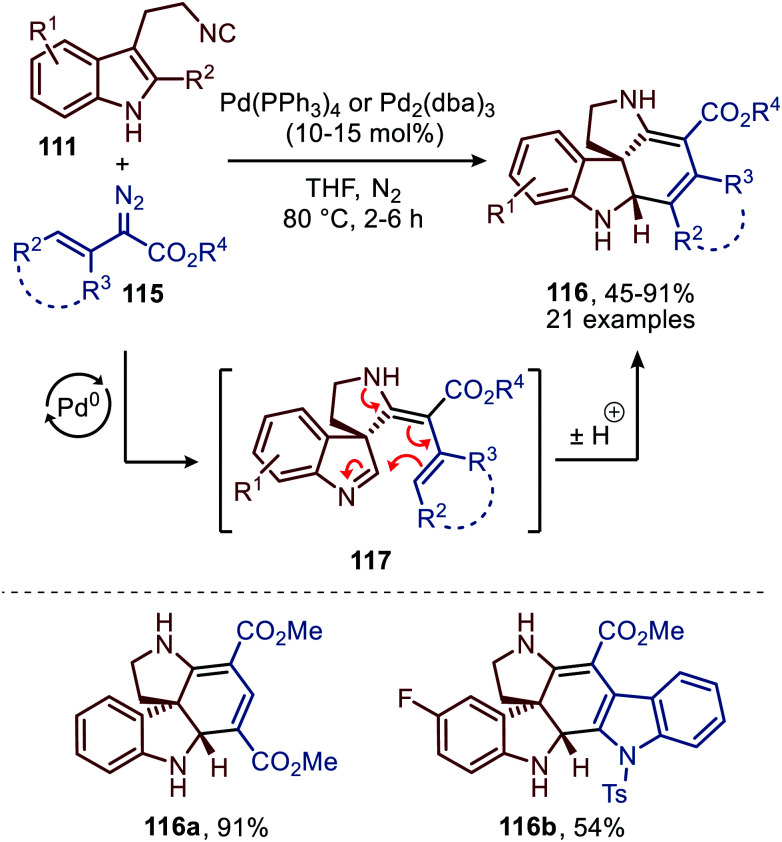
Carbene-transfer/Spirocyclisation/Mannich cascade using α-unsaturated diazo esters 115 and isocyanide 111.

Noteworthy is that the authors also demonstrated that spiroindolenines 113 could undergo a subsequent annulation in a Mannich type fashion when α-vinyl-, α-aryl and α-heteroaryl diazoacetates (115) were employed (Scheme 27).^55^ Formation of spiroindolenine 117 (analogous to 113) is followed by a Mannich type cyclisation on the 3*H*-indole affording the corresponding tetra-cyclic spiroindoline scaffolds 116 in moderate to excellent yield.

**Scheme 28:**
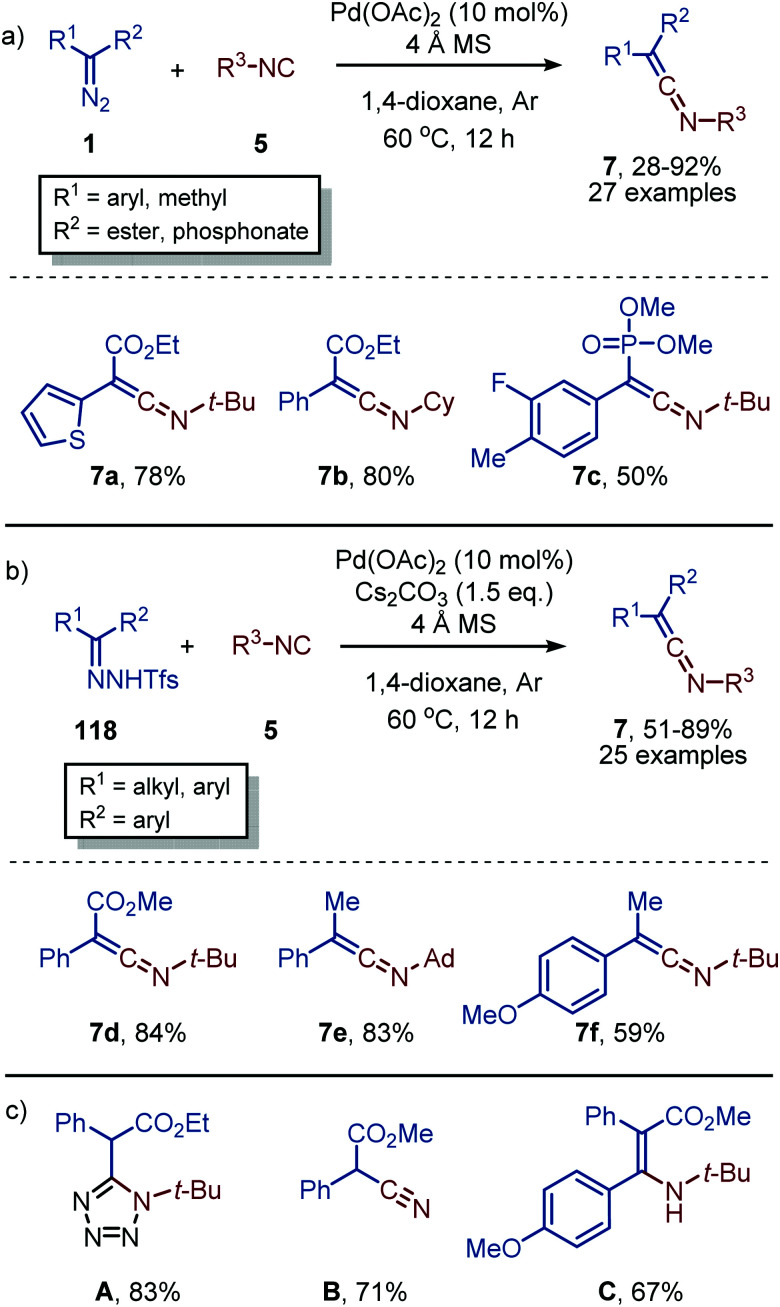
Pd^II^-Catalysed formation of ketenimines (7) from isocyanides (5) and carbenes derived from (a) diazo compounds 1 and (b) *N*-triftosylhydrazones 118. (c) Synthetic transformations from ketenimine 7.

Recently, Bi and co-workers reported the facile Pd^II^-catalysed synthesis and isolation of ketenimines (7), without further *in situ* transformation. α-Diazo compounds (1) were used as carbene precursor featuring an electron-withdrawing ester or phosphonate moiety (Scheme 28a).^56^ Similarly, they reported the use of *N*-triftosylhydrazones (118) for carbenes bearing electron-donating aryl- and alkyl substituents (Scheme 28b).^56^*N*-triftosylhydrazones were used as novel bench-stable non-stabilized diazo surrogates,^57^ omitting the need for an α-electron-withdrawing group. It is interesting that the ketenimines (7) synthesised were isolated in moderate to excellent yields, without any decomposition, which is typically observed upon isolation. The isocyanide scope is predominantly limited to tertiary and secondary isocyanides. However, limited examples with aromatic isocyanides afforded the corresponding ketenimines, albeit in low yield. The synthetic utility of ketenimines 7 was illustrated (Scheme 28c) *via* multiple transformations such as a [3+2] cycloaddition towards tetrazoles A, the cleavage of the ketenimine N–C bond to afford ethyl 2-cyano-2-phenylacetate B, or a Grignard addition towards a β-amino-acrylate C. Experimental and computational mechanistic studies suggest the *in situ* formation of a Pd^II^-isocyanide complex prior to the formation of a Pd–carbene species, suggesting that the mechanism proceeds *via* route I in our general proposed cycle (Scheme 2). According to their DFT studies, a η^2^-metallacycle intermediate (13) (Scheme 3a) was not observed and the 1,1-migratoy insertion step proceeds *via* a η^2^-metallacycle transition state instead.

**Scheme 29:**
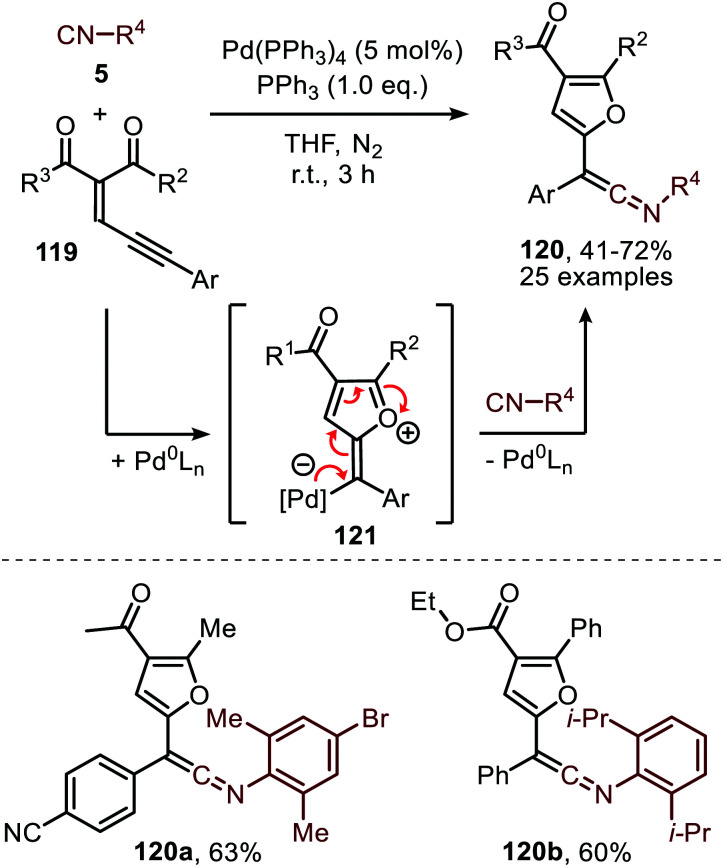
Pd^0^-Catalysed synthesis of stable ketenimines 120 using enyne 119 as carbene source.

As was demonstrated for the Rh-catalysed carbonylation of carbenes (Scheme 11) enynes can be utilized as an alternative carbene source.^58^ Li *et al.* demonstrated that enyne 119 could be transformed into furan-containing ketenimines 120 (Scheme 29). Carbene formation commences with activation of the alkyne moiety *via* a Pd-π complexation, which allows for the intramolecular attack of one of the carbonyl oxygens to the triple bond. This results in zwitterionic species 121, which in turn gives the corresponding carbene that subsequently transfers to the isocyanide resulting in ketenimines 120. The authors propose the reaction to proceed *via* route II (Scheme 2). However, based on the provided experimental data, the other pathways cannot be excluded. The authors report that aliphatic isocyanides and less bulky aromatic isocyanides are not tolerated in this catalytic transformation. The ketenimine could be isolated and was not transformed *in situ*. When ketenimine 120 is heated in the presence of a second isocyanide fragment (5), a [4+1] annulation occurs to furnish indenone scaffolds upon acid hydrolysis (not shown).

In this section, we showed that ketenimines can be versatile building blocks towards important functional groups (*i.e.* amide, amidine, imidate) and for the synthesis of numerous heterocycles (*i.e.* isoquinolines, imidazoles, furanes, carbazoles, spiroindolenines). Access of heteroallene 7*via* carbene transfer to isocyanides is predominantly catalysed by either precious Rh or Pd. For the Rh-catalysed carbene transfer to isocyanides, Rh^I^-complexes are typically employed and the group transfer reaction presumably proceed *via* route I as multiple authors assume that the isocyano complex is the active catalytic species. Noteworthy is that for the Rh^I^-catalysed processes, only aromatic or vinylic isocyanides were tolerated, whereas aliphatic isocyanides did not lead to product formation. In addition to Rh, Pd-catalysed carbene transfer to isocyanides are often encountered. Depending on the transformation either aliphatic or aromatic isocyanides are accepted. In some cases, both type of isocyanides are tolerated, but there typically is a large preference for one of the two. A direct rationalisation for this preference is not provided and will require a deeper understanding of the catalysis. The exact nature of the catalytic active species is in most Pd-catalysed examples illusive and the reaction can either proceed *via* route I or II. However, DFT calculations performed by Bi *et al.*^56^ indicate that a Pd-isocyano complex is the active species, thereby favoring route I (Scheme 2).

For the base-metals, a simple Co^II^-salt can catalyse the carbene transfer reaction to isocyanides with high functional group compatibility. For CoBr_2_ (Scheme 16) a Co-isocyano complex is believed to be the active species,^47^ therefore, favouring route I (Scheme 2) as mechanism for the group transfer reaction. However, further research in base-metal catalysis is definately required. The report on Ni catalysis shows feasibility, though there was still a focus on stoichiometric reactions. This will concomitantly stimulate the development of additional applications for *in situ* transformations of the ketenimine products. A notable advantage for the Co-catalysed example is the use of relatively cheap cobalt salt without involvement of additional ligands or additives, which are typically required for the noble-metals, such as Rh and Pd.

Noteworthy is that several non-metal-based processes have been reported in addition to the TM-catalysed processes. The feasibility of this inherently attractive approach towards ketenimines highly depends on the structure of the carbene formed. This include the direct addition of isocyanides to carbenes, *i.e.* various di(amino)carbenes, di(amido)carbenes and (alkyl)(amino)carbenes, under mild conditions.^59^ Metal free carbene formation from diazo compounds using direct blue light excitation typically starting from donor-acceptor α-aryl diazoacetates delivers both singlet and triplet carbenes. These carbenes can participate in a wide variety of transformations, *e.g.* in the cyclopropanation of alkenes and alkynes, in the Doyle Kirmse rearrangement with allylic thioethers and insertion in O–H, N–H and C–H bonds.^60^ However, extending this to the transfer to isocyanides afforded the corresponding ketenimines in only low to moderate isolated yields (12–64%).^61^ The authors mention that the low yields are partly caused by a low selectivity of the free carbene as they do observe by-product formation *via* carbene dimerization. Besides reaction of two diazo compounds into a ketazine and degradation of the triplet ketenimine *via* homolytical C–N single bond fragmentation was noted.^61^ Transition metals such as Pd under thermal conditions typically aid in the circumvention of these unwanted side reactions and higher yields for target ketenimine formation plus a broader substrate scope were observed, albeit under typically harsher conditions than room temperature.^56^

## 3 Transition-metal catalysed carbonylation and imidoylation of nitrenes

In this section, the nitrene transfer to carbon monoxide and isocyanides (5) will be discussed. *In situ* transformations proceeding either through heteroallene intermediate 8 and 9 (Scheme 1) will be categorized according to the transition metal used. In addition, mechanistic discrepancies with regard to the general catalytic cycle of Scheme 2 will be highlighted when applicable.

### 3.1. Nitrene transfer to carbon monoxide

Carbonylation of nitrenes result in the formation of an isocyanate fragment (8) (Scheme 1). Isocyanates were already discovered in 1849 by Wurtz^62^ and are industrially typically synthesised *via* stoichiometric reaction of an amine with phosgene generating corrosive HCl by-product.^20*a*^ Considering its toxicity, alternatives have been actively sought for.^20*b*^ The transition metal-catalysed carbonylation reaction of nitrenes is one of these interesting alternatives in the formation of 8 as gaseous benign N_2_ is the by-product. The *in situ* transformation of the heteroallene 8 avoids the isolation of inherently toxic isocyanates and is, therefore, very appealing. In polymer chemistry blocked isocyanates are an active domain of contemporary research to avoid using diisocyante monomers and hereby reduce the hazards of isocyanates.^63^

#### Iron

**Scheme 30:**
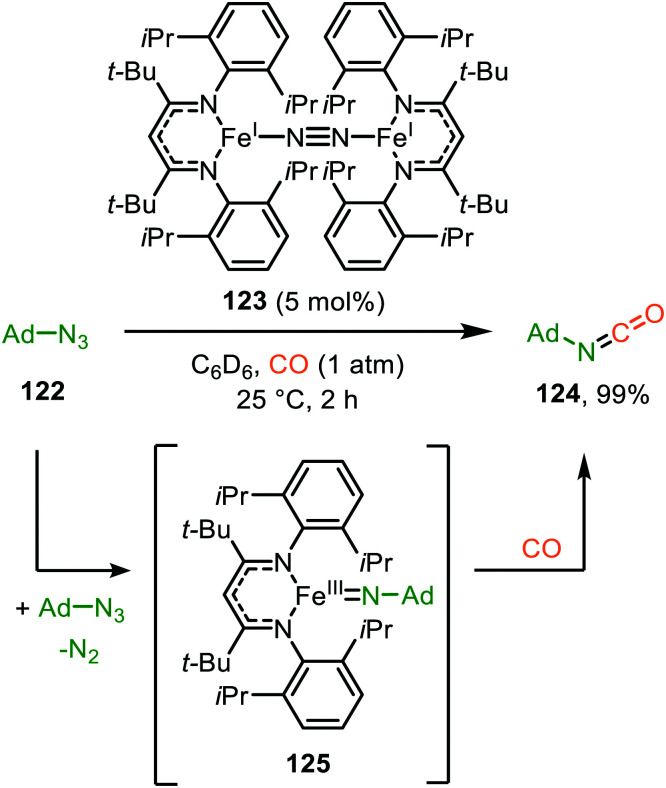
Fe^I^-Catalysed formation of adamantly isocyanate 124*via* Fe^III^-imido intermediate 125.

This is one of the most abundant base-metals on Earth,^64^ making its utilization highly interesting from an economical as well as ecological point of view. In addition, iron is able to adopt many oxidation states, resulting in versatile reactivity. Therefore, catalytic transformations with iron are highly desired alternatives to noble metal-catalysed processes. However, only one report is published. Holland *et al.* report the catalytic transformation of adamantyl azide (122) and CO into isocyanate 124 in excellent yield, using iron catalyst 123 (Scheme 30).^65^ The reaction proceeds *via* mono Fe-imido complex 125. The authors did not perform an optimisation of the catalytic system, nor do they report any substrate scope. However, based on their catalyst and stoichiometric experiments, imido-complex 125 is formed prior to coordination of CO, indicating that the mechanism follows route II in Scheme 2.

#### Molybdenum

**Scheme 31:**
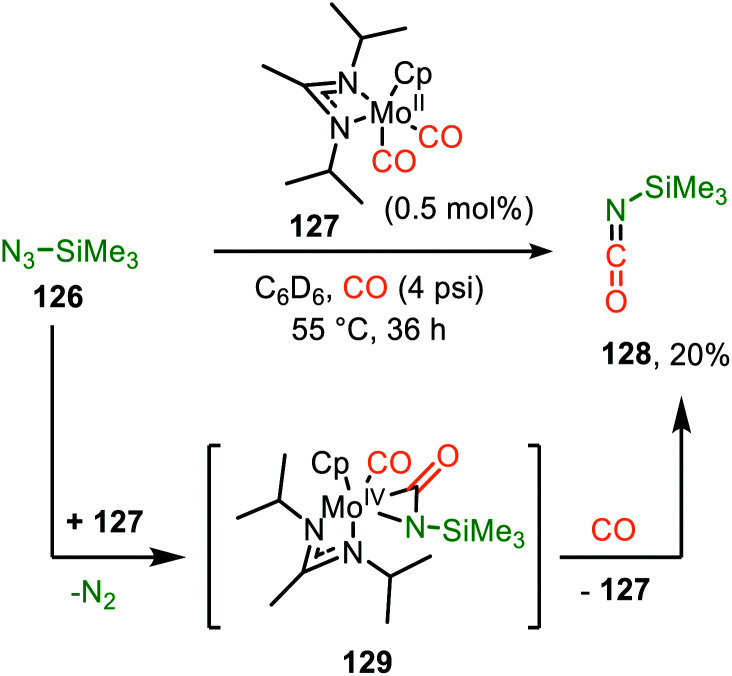
Mo^II^-Catalysed nitrene transfer from trimethylsilylazide proceeding *via* metallaaziridine intermediate 129.

A second still rarely encountered transition metal for the carbonylation and imidoylation of nitrenes is base metal Mo. Mo^II^-complex 127 was employed by the group of Sita to produce isocyanate 128 (Scheme 31).^66^ The authors demonstrate that isocyanate 128 could be obtained under catalytic conditions when treating trimethylsilyl azide (126) under CO atmosphere, albeit in low yield (Scheme 31). The proposed mechanism is mainly based on precedents in literature, and presumably proceeds *via* η^2^-coordinated metallacycle intermediate 129 (Scheme 31), accessed *via* route I, starting from intermediate 10 of our general mechanism (Scheme 2). Unfortunately, little comment is made on the catalytic cycle that should be operating and synthetic applicability of this Mo^II^-based system.

In comparison to the base-metal catalysed examples of carbene transfer to CO, only two catalytic examples are disclosed with nitrenes. Further research into base-metal catalysis, including ligand design, is therefore required. Nowadays, row V noble metals, such as palladium, offer more reliable transformations with a broader applicability.

#### Palladium

**Scheme 32:**
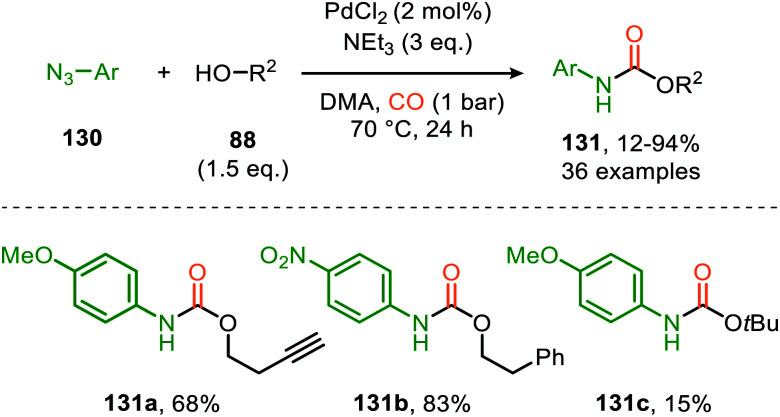
Pd-Catalysed synthesis of carbamates 131 from azides (130), alcohols (88) and CO.

Until today Pd proved to be the superior TM for the nitrene transfer towards CO. Jiao *et al.* demonstrated the use of PdCl_2_ for the synthesis of carbamates 131, starting from aryl azides (130) and alcohols (88) (Scheme 32).^67^ The reaction tolerates functionalized primary, secondary, and tertiary alcohols, albeit the yield decreases with increase of steric bulk. In addition, the system is susceptible to aromatic azides (130) with different electronic properties, performing exceptionally well in the reaction (60–94%). Noteworthy is that under the given conditions, heteroaryl- and alkenyl azides are also accepted. However, primary alkyl- and benzyl-azides afforded carbamates 131 in rather low yields (12–37%). The authors rationalize this result by the poor stabilisation of the metal nitrene intermediate by the alkyl group (R^1^), which is crucial for a facile transfer to CO. Different Pd-sources, such as Pd(PPh_3_)_4_, show no conversion towards carbamate 131. This indicates that a specific *in situ* generated complex is the active species, of which the exact structure is unknown. The postulated mechanism is solely based on literature precedents as no experimental nor computational mechanistic studies were performed. Both route I and II of the general catalytic cycle are possible (Scheme 2), the η^2^-isocyanate metal complex (13) is proposed to be a key intermediate (Scheme 3a) transforming into carbamate 131*via* alcohol addition.

**Scheme 33:**
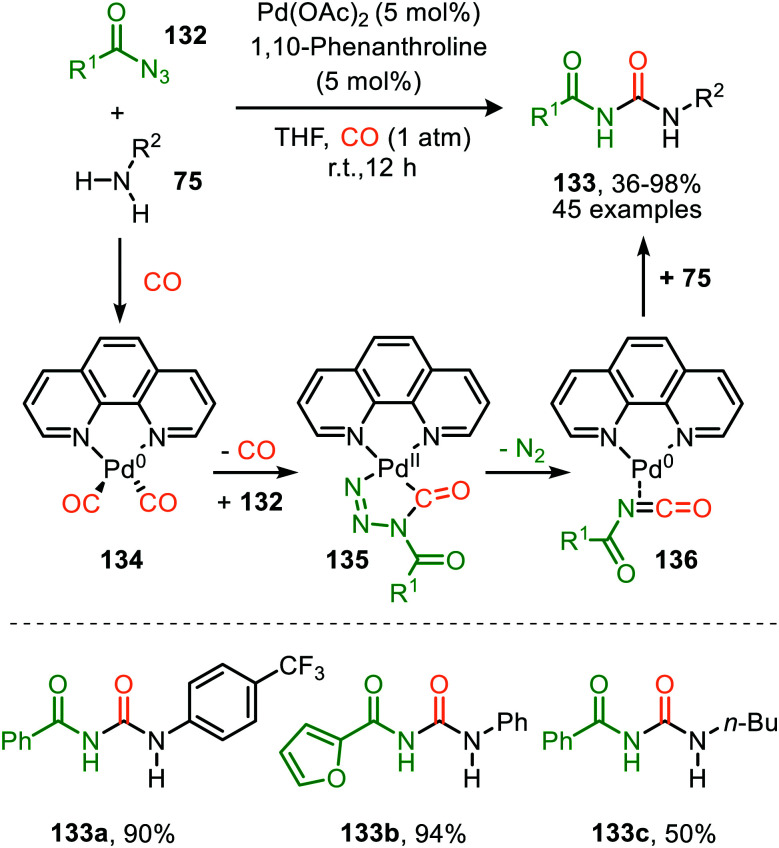
*N*-Acyl urea (133) synthesis *via* Pd^0^-catalysed carbonylation of acyl nitrene.

**Scheme 34:**
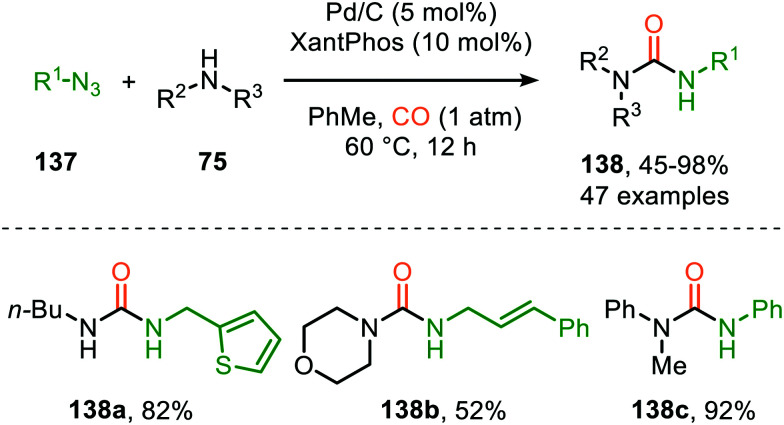
Synthesis of urea derivatives 138*via* Pd^0^-catalysed nitrene transfer to CO.

Zhang and co-workers report the use of Pd(OAc)_2_ and 1,10-phenanthroline as bidentate ligand for the synthesis of *N*-acylated urea derivatives 133 from acyl azides (132) employing amine nucleophiles (Scheme 33).^68^ A wide variety of electron-withdrawing and electron-donating acyl azides were tolerated providing moderate to excellent yields. Aromatic amines as well as (cyclic) aliphatic amines were compatible as nucleophiles under the reaction conditions. The authors base their proposed mechanism on kinetic and computational studies and suggest that Pd^0^-species 134 (Scheme 33) is the active species. Pd-complex 134 forms prior to coordination of acyl azide 132. Interestingly, instead of the formation of the conventional monometallic nitrene fragment followed by 1,1-migratory insertion of CO (Route I, Scheme 2), the authors suggest that acyl azide 132 reacts with the L_*n*_Pd–CO species 134 in an oxidative cyclisation step to provide five-membered metallacycle 135, from which *via* reduction L_*n*_Pd coordinated acyl isocyanate 136 and molecular nitrogen is formed (Scheme 33). The coordinated isocyanate intermediate 136 de-coordinates the TM-centre and is then captured by the amine to obtain *N*-acylated urea 133. The same authors also developed a different catalytic system based on heterogeneous Pd/C and XantPhos ligand for the synthesis of urea derivatives 138 (Scheme 34).^69^ The reaction accepts aromatic-, benzylic and aliphatic azides (137) and delivers ureas 138 in moderate to excellent yields (45–98%). The proposed mechanism may follow either route I or II of the general catalytic cycle (Scheme 2). However, no experimental or computational data is provided to support the proposed catalytic cycle. The authors propose that a Pd leaching process is highly likely.^70^ Presumably, Xantphos scavenges Pd from the carbon support generating a homogeneous catalyst.

**Scheme 35:**
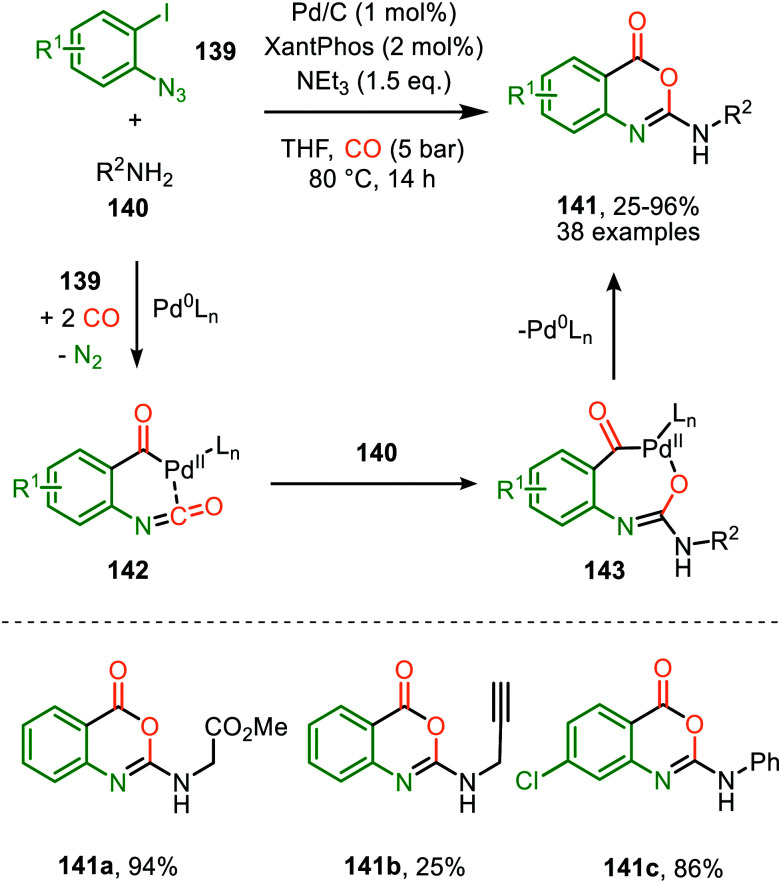
Pd^0^-Catalysed synthesis of 2-aminobenzoxazinones (141) *via* carbonylation of nitrene generated from 1-azido-2-iodobenzene (139).

A similar catalytic system, based on Pd/C as Pd^0^ source, was applied for the carbonylative synthesis of 2-aminobenzoxazinones (141) from 1-azido-2-iodobenzenes (139) and primary amines (140) by Wu and co-workers (Scheme 35), though mechanistically it is completely different.^71^ The scope of 1-azido-2-iodobenzenes (139) is limited. However, high tolerance is observed for a wide variety of primary, secondary and (cyclic) aliphatic-, and (hetero)aromatic amines. Some examples with interesting functional handles are depicted in Scheme 35. The proposed mechanism is initiated by oxidative addition of 1-azido-2-iodobenzene (139) to Pd^0^L_*n*_, followed by subsequent 1,1-migratory insertion of CO into the Pd–C σ-bond. This is followed by nitrene transfer to CO, affording intermediate 142 (Scheme 35). Subsequent nucleophilic attack of amine 140 results in palladacycle 143, which upon reductive elimination furnishes 2-aminobenzoxazinones (141). The mechanism is not investigated extensively, and key processes involved, such as isocyanate formation are not addressed.

**Scheme 36:**
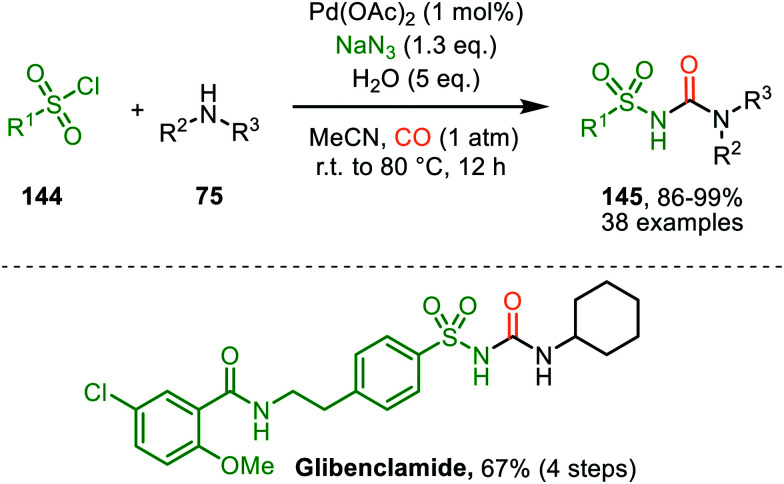
Pd^II^-Catalysed synthesis of *N*-sulfonyl urea (145) *via* carbonylation of sulfonyl nitrenes from *in situ* generated sulfonyl azides.

**Scheme 37:**
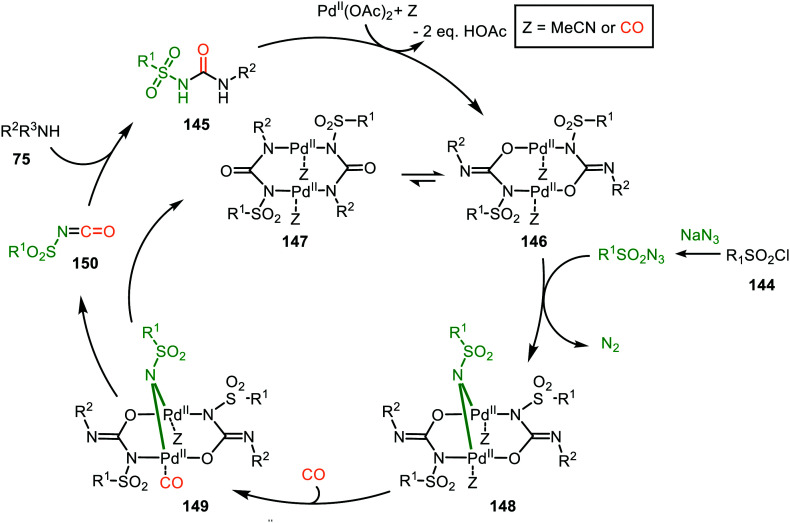
Proposed mechanism of the bi-metallic Pd^II^ complex 146/147-catalysed nitrene transfer to CO in the synthesis of *N*-sulfonyl urea (145).

These authors also reported a Pd-catalysed nitrene transfer for the formation of *N*-sulfonyl ureas 145, which involves the *in situ* formation of the sulfonyl azide from sulfonyl chloride 144 and NaN_3_ (Scheme 36).^72^ The reaction tolerated numerous substituents on both the sulfonyl as the amine moiety and the corresponding urea 145 is obtained in excellent yields in all cases. The authors demonstrate the synthetic viability of their method by synthesising Glibenclamide with an overall yield of 67% in four steps. The proposed mechanism is based on precedents in literature, HRMS and NMR-studies, and control experiments (Scheme 37). Based on their studies the authors state that the active catalytic species consists of bimetallic Pd-complex, where the urea product 145 acts as a ligand. This was supported by preformation of bimetallic *N*-sulfonylurea complex prior to nitrene transfer, which was far more superior than the system based on Pd(OAc)_2_ in terms of rate of product formation.

The proposed mechanism commences with the formation of a bi-metallic Pd-complex, where both the *N*,*N*-donor form 147 or the *N*,*O*-donor 146 can be present, and the *N*,*O*-form 146 is the dominant active species according to the authors. Subsequently, bridged nitrene complex 148 is formed from complex 146/147 and *in situ* generated sulfonyl azide. Consecutively, CO coordinates to one of the Pd-centres providing 149 followed by 1,1-migratory insertion into one of the Pd–N bonds, affording isocyanate 150. As CO can already coordinate to the Pd-centre prior to nitrene formation, either route I or II (Scheme 2) is in good agreement with the mechanism depicted in Scheme 37.

**Scheme 38:**
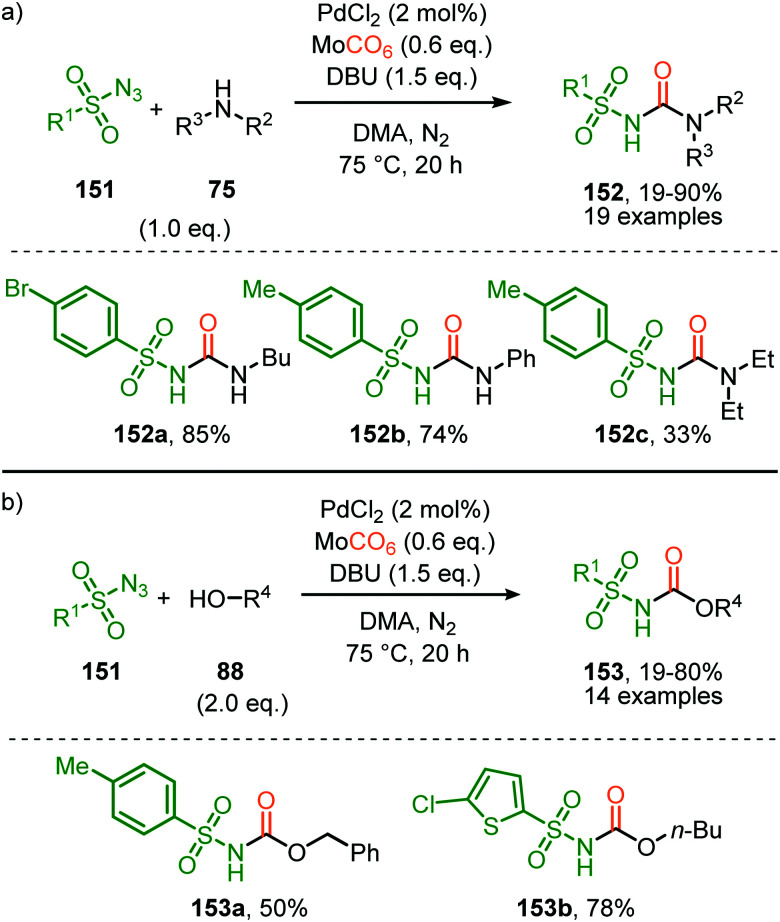
Pd^0^-Catalysed formation of: (a) *N*-sulfonyl urea (152) and (b) *N*-sulfonyl carbamates (153).

In the previous examples, CO was introduced as a gas. However, Odell *et al.* demonstrated the use of Mo(CO)_6_ as non-gaseous CO-source for the synthesis of *N*-sulfonyl ureas (152) and *N*-sulfonyl carbamates (153) from sulfonyl azides 151*via* a Pd-catalysed nitrene transfer to CO (Scheme 38).^73^ For both the synthesis of *N*-sulfonyl urea (152) and *N*-sulfonyl carbamates (153), azides 151 bearing a (hetero)arenesulfonyl moiety perform well in the reaction. Primary and secondary aliphatic and aromatic amines are generally accepted in the transformation of *in situ* generated isocyanate into urea. With respect to the carbamate synthesis, primary-, secondary-, and tertiary aliphatic alcohols are generally tolerated in the reaction, whereas phenol did not afford any product. Their proposed mechanism, based on literature precedents and control experiments, shows that the active catalyst is a Pd^0^ species. The mechanism is initiated by the reduction of the pre-catalyst PdCl_2_ to an active Pd^0^L_*n*_ species, followed by the generation of a nitrene-palladium complex from *N*-sulfonyl azide 151. Subsequent CO coordination and 1,1-migratory insertion delivers a sulfonyl isocyanate, which undergoes nucleophilic attack with amines (75) or alcohols (88) to deliver the corresponding final products. It remains unclear what the exact nature of the active catalytic species is during the reaction as either CO, the azide or the formed products could be involved in generating the active catalytic species.

To conclude, several transition metals can catalyse the nitrene transfer to CO but only palladium is yet well explored. For the Mo-catalysed system the yield was low and no *in situ* transformation of the heteroallene was reported. At first sight, iron seems to be an efficient and promising catalyst, however, no cascade processes have been developed yet. Detailed mechanistic studies point towards route II as the Fe-imido complex forms prior to involvement of CO (Scheme 30). Overall, when looking at the transition metals employed, only palladium has established itself as a mature catalyst in the formation of isocyanates *via* nitrene transfer to CO. The presented catalytic systems also allow *in situ* trapping of isocyanate (8) with various nucleophiles as is demonstrated in the formation of carbamates, urea derivatives and aminobenzoxazinones (141). The proposed mechanisms indicate that the nitrene transfer can either proceed *via* route I or II (Scheme 2). Interestingly, when a ligand was applied it was of the bidentate type.

### 3.2. Nitrene transfer to isocyanides

So far, we described in this review the formation of several heteroallenes *via* TM catalysis: ketenes (6) and ketenimines (7) *via* carbene transfer to CO and isocyanides respectively, and isocyanates (8) *via* nitrene transfer to CO. Imidoylation of nitrenes affording carbodiimides (9) is also known and described in this section (Scheme 1). Carbodiimides received only limited attention until the late 50's, but are nowadays a widespread building block and reagent in organic synthesis.^18^ Common stoichiometric methods^18^ for the synthesis of carbodiimide are for example the dehydrosulfirisation of thioureas,^74^ the rearrangement of *N*-substituted amidoximes (Tiemann rearrangement) *via* transformation of the hydroxy into a leaving group and the oxidative rearrangement of *N*-substituted amidines.^75^ Noteworthy, transition metal mediated processes towards (metallated) carbodiimides (9) involving organoazide species and isocyanides have been covered in a review by Beck *et al.* in 2015,^76^ but no catalytic examples or cascade processes with the formed 9 were included.

#### Titanium

**Scheme 39:**
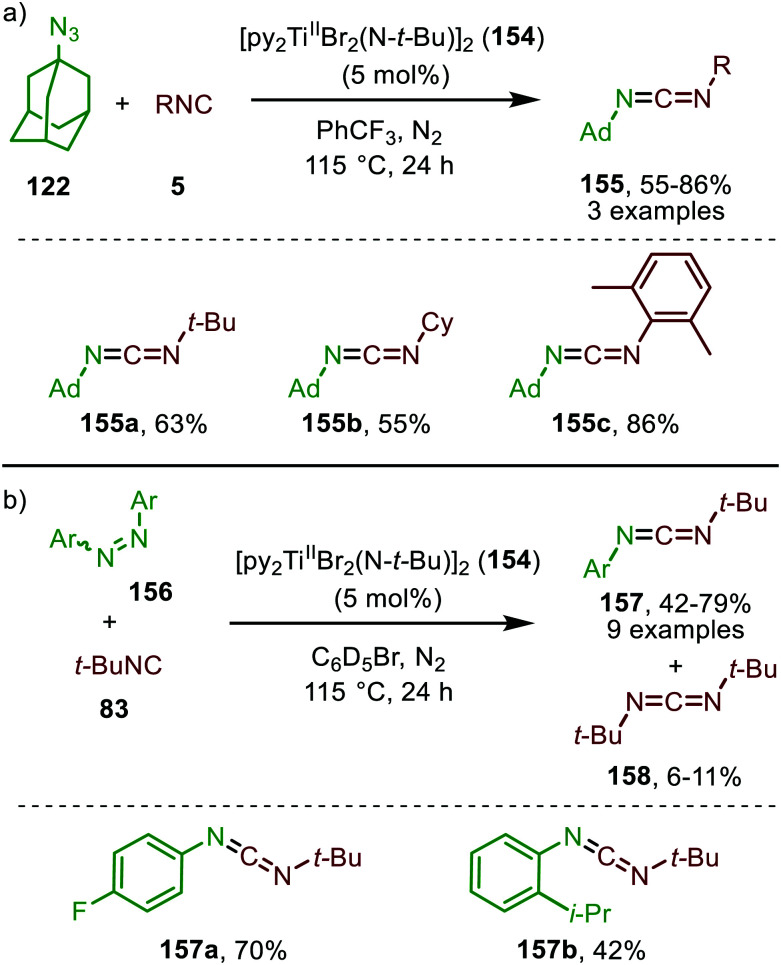
Carbodiimide synthesis *via* Ti^II^-catalysed imidoylation of nitrenes using: (a) adamantyl azide (122) and (b) diazenes (156).

Early TMs are less frequently applied as catalyst for group transfer reactions towards CO and isocyanides. However, the work of Tonks *et al.* illustrated that Ti^II^-catalyst 154 is able to realise nitrene transfer to isocyanides, using adamantyl azide (122) (Scheme 39a) or diazenes (156) (Scheme 39b) as nitrene precursor for the synthesis of carbodiimides (155 and 157), respecitvely.^77^

**Scheme 40:**
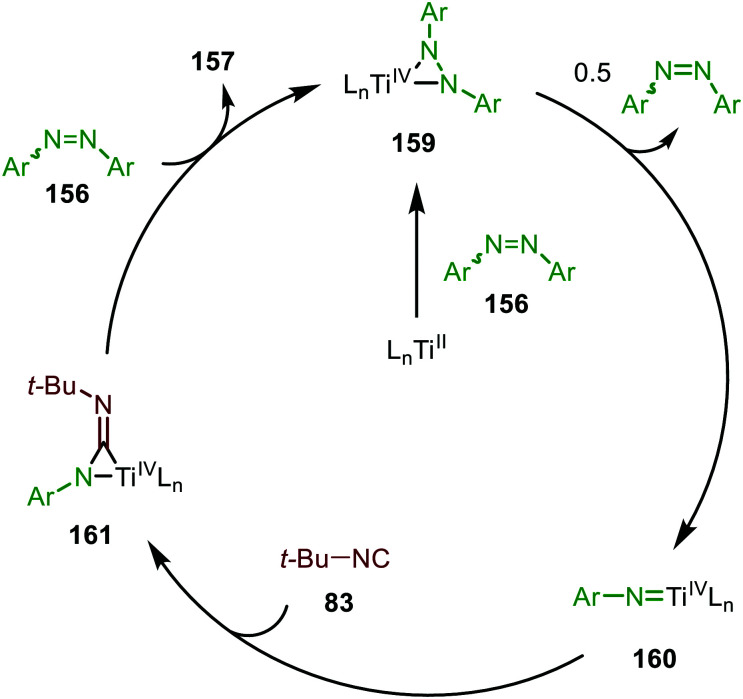
Proposed mechanism of the Ti^IV^-catalysed carbodiimide formation from diazenes (156) and *t*-BuNC (83).

The adamantyl azide (122) reacts either with cyclohexyl isocyanide, *tert*-butyl isocyanide, or aromatic 2,6-xylyl isocyanide to afford the corresponding carbodiimides (155) in good yield (Scheme 39a). Important note is that sterically encumbered azide 122 is crucial to prevent catalyst inhibition *via* multiple isocyanide coordination. In the nitrene transfer with diazenes, the reaction tolerates aromatic diazenes 156 bearing aliphatic- or inductively electron-withdrawing substituents on the aryl moiety (Scheme 39b). The formation of carbodiimides 157 deviates from the general proposed mechanism in Scheme 2 as here a different activation mode of nitrene precursor 156 is involved. Extensive mechanistic studies rationalize the formation and further transformation of Ti-imido complexes from diazenes 156.^77–79^ The catalytic cycle commences with oxidative addition of diazene 156 to the Ti^II^-centre forming Ti^IV^-species 159 (Scheme 40). Subsequently, Ti^IV^-imido complex 160 is formed by formally liberating 0.5 equivalent of diazene. Unfortunately, the authors provide no further comments to support this step. Coordination of isocyanide 83, and subsequent 1,1-migratory insertion, provides the η^2^-carbodiimide intermediate complex 161, which is in accordance with route II of the general cycle in Scheme 2. Subsequent release of the carbodiimide proceeds in an associative fashion involving diazene 156. The release is triggered by an electron transfer from the Ti-bound η^2^-carbodiimide (161) to the Ti-coordinated diazene ligand, releasing carbodiimide 157 and forming complex 159. Noteworthy is that the associative release prevents the formation of a discrete Ti^II^-intermediate.^77^

**Scheme 41:**
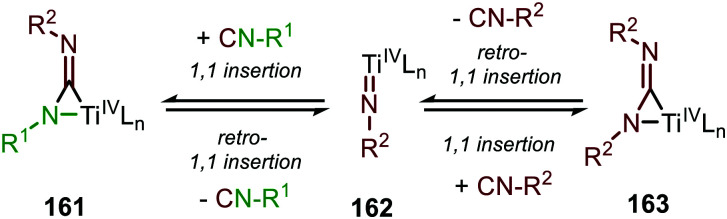
Metathesis mechanism of isocyanide scrambling *via* a retro-1,1-migratory insertion.

Another important note is the formation of homo-coupled by-product 158, with diazene 156 as nitrene source (Scheme 39b). The authors suggest the formation of the by-product *via* an uncommon isocyanide metathesis mechanism (Scheme 41). The occurrence of this retro 1,1-insertion, also known in the literature as *isocyanide scrambling*, implies the presence of a η^2^-carbodiimide intermediate (161) in the catalytic cycle as commonly proposed in the general mechanism (13, Scheme 3a.)

#### Chromium

**Scheme 42:**
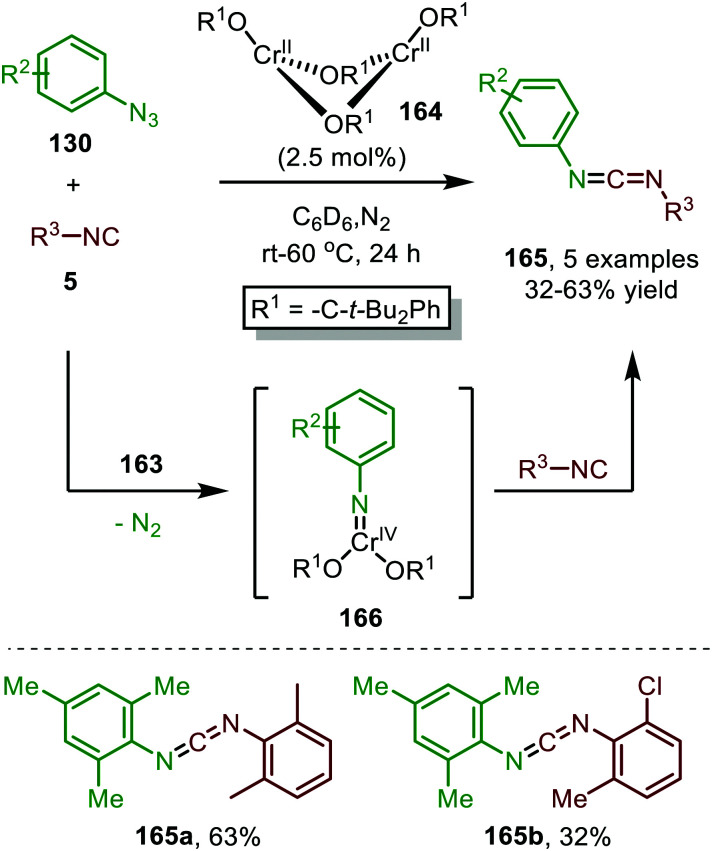
Cr^II^-Catalysed nitrene transfer of aromatic azides 130 to isocyanides 5 providing carbodiimides 165.

In 2015, Groysman *et al.* reported the coupling of aryl isocyanides with aryl azides 130 in the synthesis of asymmetrically substituted *N*-aryl carbodiimides 165, utilising bis-alkoxide Cr^II^-complex (164) as pre-catalyst (Scheme 42).^80^ The authors postulate that the pre-catalyst is converted to active mono-Cr^IV^-imido species 166 by reaction with aryl azides (130).

This trigonal-planar imido-Cr^IV^ intermediate 166 was isolated and fully characterized by the authors and could only be formed with sterically demanding aromatic azides. Decreasing the steric bulk of the azides or the alkoxide ligand of the pre-catalyst, resulted in the formation of a coordinatively saturated Cr^VI^-bis(imido) complex, which was unreactive in the subsequent nitrene-transfer step. However, the formation of an isocyano complex prior to nitrene formation could not be ruled out and the mechanism could proceed *via* either route I or II in Scheme 2. Although the scope of azides and isocyanides is limited, the authors nicely demonstrate the use of high valent early transition metals for catalytic nitrene transfer, which is inherently more difficult than with low valent late TM's. This is due to the increased stability of the TM-nitrene fragment with early TM's, compared to late TM's. The stronger bonding interactions between the nitrene fragment and early TM's in a high oxidation state are due to the availability of empty metal d-orbitals and strong σ- and π-coordination of the imido group.^80^

**Scheme 43:**
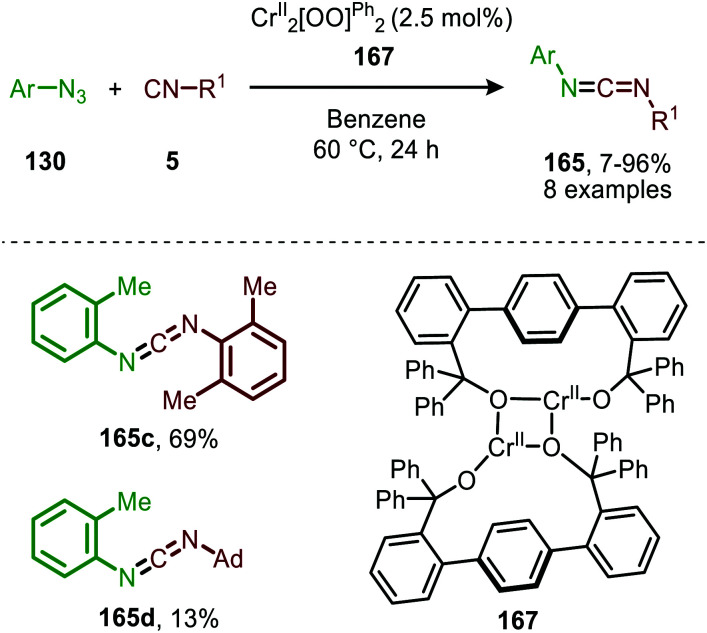
Cr^II^-Catalysed coupling of azides (130) and isocyanides (5) forming carbodiimides (165).

In 2020 the same research group reported the use of dichromium complex 167 with chelating bis(alkoxide) ligands in the context of catalytic synthesis of carbodiimides (165) from azides (130) and isocyanides (5) (Scheme 43).^81^ The authors observed that only sterically encumbered azides were able to participate in the nitrene transfer reaction. Based on their previous work (Scheme 42) the authors postulate that non-bulky azides form a saturated catalytically inactive Cr^IV^-bis(imido) complex. The tolerated isocyanides also beared sterically demanding electron-rich aromatic, secondary aliphatic or tertiary aliphatic substituents. The proposed mechanism by the authors is in accordance with route II of our general mechanism in Scheme 2.

#### Iron

**Scheme 44:**
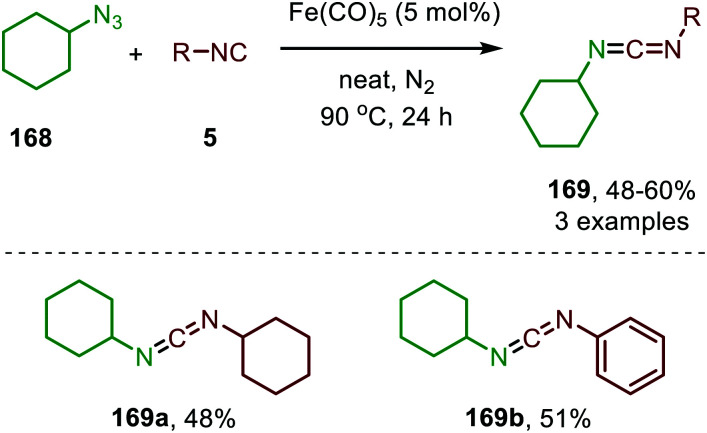
Fe^0^-catalysed synthesis of unsymmetrical carbodiimides (169) from isocyanides (5) and cyclohexyl azide (168).

The use of late TM low-valent Fe-species for nitrene transfer to isocyanides was already discovered in 1970 by Saegusa *et al.* (Scheme 44).^82^ Fe(CO)_5_ turned out to be a suitable catalyst. The nitrene coupling reaction was investigated with cyclohexyl azide (168) as nitrene source and several isocyanides. The corresponding non-symmetrical *N*-cyclohexyl aryl/alkylcarbodiimides (169) were obtained in moderate isolated yields. The authors did not comment on a plausible mechanism for this transformation.

**Scheme 45:**
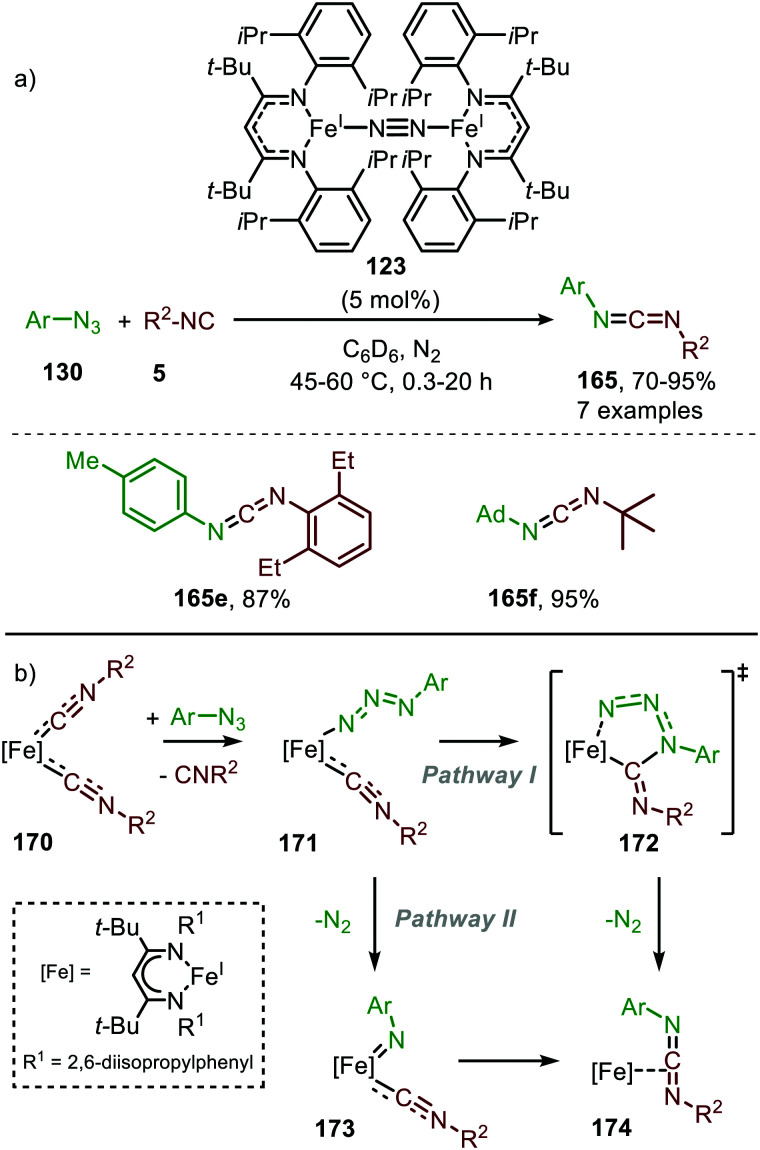
(a) Fe^I^-Catalysed carbodiimide (165) synthesis *via* nitrene transfer to isocyanides. (b) Postulated pathways towards Fe-carbodiimide (174).

It took until 2013 to follow-up on this early iron-based nitrene transfer. Holland and co-workers developed a bimetallic β-ketiminate ligand supported Fe^I^ complex 123 (Scheme 45a) to achieve a catalytic nitrene transfer to isocyanides providing carbodiimides 165.^83^ A brief scope study of the reaction under optimal reaction conditions indicates that the bulky tertiary aliphatic and sterically demanding aromatic azides and isocyanides perform well in the reaction (70–95%). The authors state that sterically hindered isocyanides are crucial to prevent the formation of a saturated and inactive tris-isocyanide Fe^I^-complex. The mechanism of the desired Fe^I^-catalysed transformation was elucidated through control experiments, EPR and NMR spectroscopy and complemented with DFT calculations. The proposed mechanism complies for the greater part with route I of our general cycle (Scheme 2). The Fe^I^-based cycle commences with the formation of mono-nuclear bis-isocyano Fe^I^-complex (170) (Scheme 45b). Subsequently, one isocyanide moiety is displaced by an aryl azide (130) to form intermediate 171. Interestingly, the authors propose that from this intermediate (171) the metallated carbodiimide (174) can be obtained directly *via* nitrogen extrusion involving a five-membered transition state 172 (Route I). Alternatively, formation of metallated carbodiimide (174) from Fe-complex 171 can occur in a stepwise fashion (Route II), which involves nitrogen extrusion to furnish nitrene intermediate 173 followed by 1,1-migratory insertion of isocyanide to provide metallated carbodiimide 174. 1,1-Migratory insertion in nitrene complex 173 proceeds *via* a 3-membered transition state (13) (Scheme 3a). Based on the provided data the authors could not discriminate between the two pathways.

#### Cobalt

**Scheme 46:**
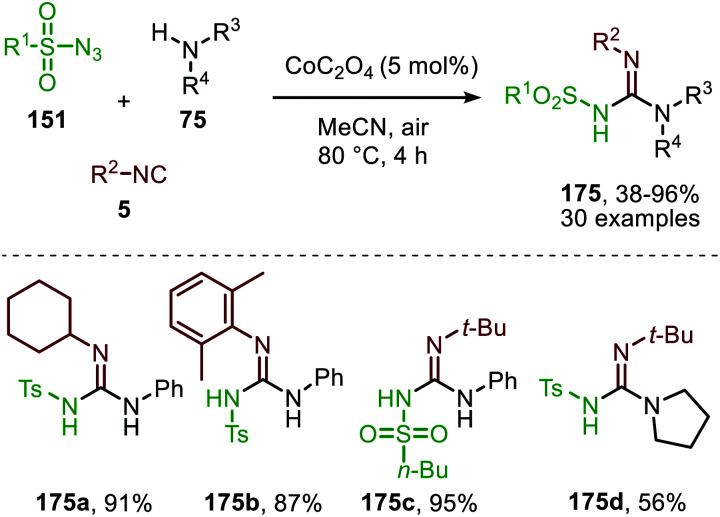
Cobalt oxalate-catalysed *N*-sulfonyl guanidine (175) formation.

Although several base-metals can catalyse the nitrene transfer to isocyanides, the presented catalytic systems until this stage did not show *in situ* transformation of the carbodiimide (9). However, in 2017 Ji *et al.* developed a three-component reaction between isocyanides (5), amines (75) and sulfonyl azides (151) under Co^II^-catalysis to afford *N*-sulfonyl guanidines (175) (Scheme 46).^84^ In contrast to previous base-metal catalysed nitrene transfer reactions, this transformation did not require specific ligands and the reaction could even be performed under air atmosphere. The transformation tolerates a wide variety of isocyanides with primary aliphatic- and α-acidic isocyanides as the only exceptions. Both aromatic- and aliphatic sulfonyl azides furnished the corresponding *N*-sulfonyl guanidines 175 in good to excellent yields. However, the yield decreased when electron poor aromatic amines or sterically encumbered cyclic secondary amines were employed.

**Scheme 47:**
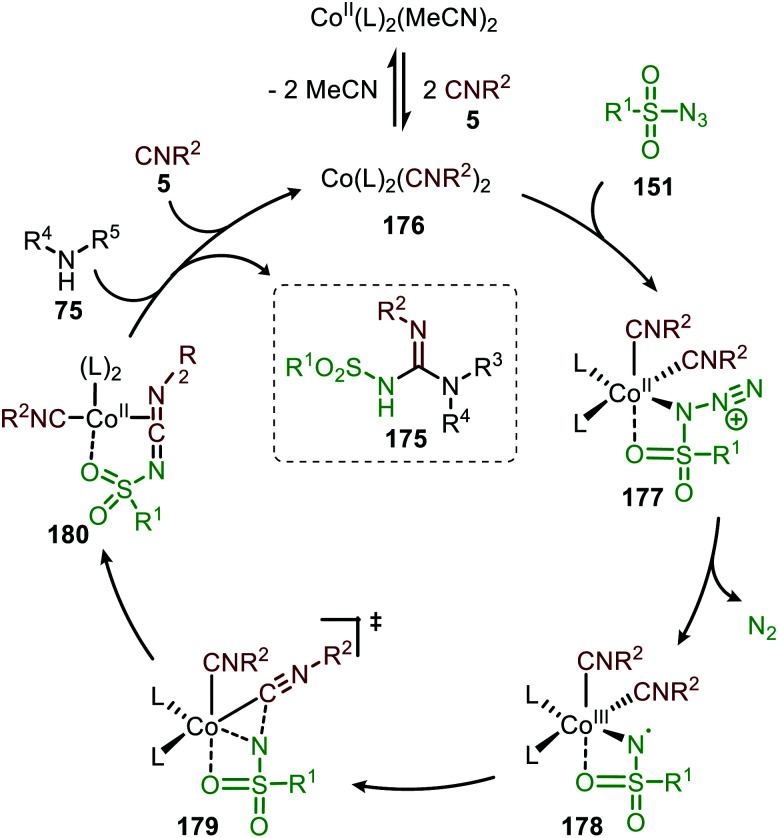
Proposed catalytic cycle of the Co^II^-catalysed nitrene transfer to isocyanides (5) *via* Co^III^–carbene radical nitrene intermediate 178.

The proposed mechanism for this Co^II^-catalysed nitrene transfer to isocyanides was based on experimental observations, EPR spectroscopy and detailed DFT-calculations (Scheme 47). Initial ligand exchange of MeCN with an isocyanide results in bis-isocyanide complex 176. This is followed by coordination of the azide (151) and extrusion of N_2_, furnishing the Co^III^-radical nitrene intermediate 178. Subsequent 1,1-migratory insertion proceeding through a η^2^-transition state (179), generates Co^II^-carbodiimide complex 180. The structure of the proposed transition state (179) is in accordance with metallacycle 13 in Scheme 3a. The metallated carbodiimide is subsequently attacked by a nucleophile furnishing the desired product and Co^II^-species 176, thereby closing the catalytic cycle. The proposed cycle is in line with route I (Scheme 2) involving a Co^III^-radical nitrene intermediate 178.

**Scheme 48:**
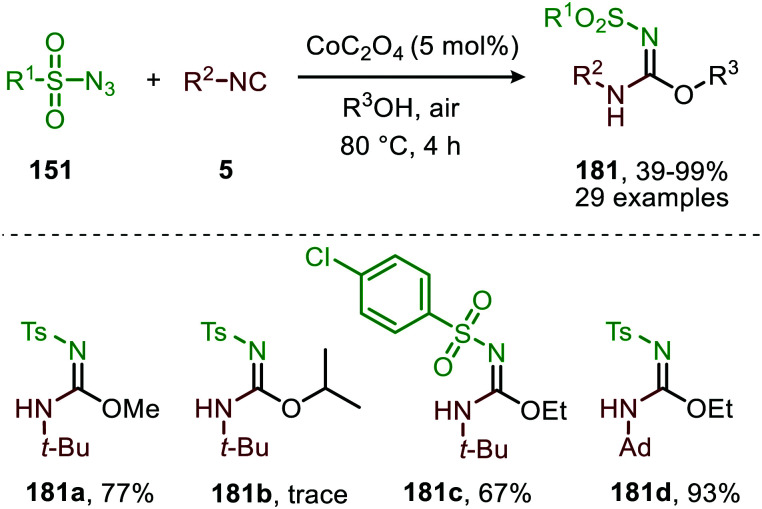
Co^II^-Catalysed imidoylation of nitrenes for the synthesis of *N*-sulfonyl isoureas (181).

The applicability of this three-component reaction was further extended by using alcohols as input instead of amines. This furnished interesting *N*-sulfonyl isoureas (181) (Scheme 48) as the products.^85^ Notably, for this reaction all types of isocyanides could be used except for primary aliphatic isocyanides, which were not compatible. In addition, a broad range of arenesulfonyl azides (151) with different electronic characteristics were well tolerated. Unfortunately, the method is limited to only primary alcohols, while secondary- and tertiary alcohols performed less efficient. Most likely this can be attributed to increased steric demand for these inputs reducing nucleophilicity.

**Scheme 49:**
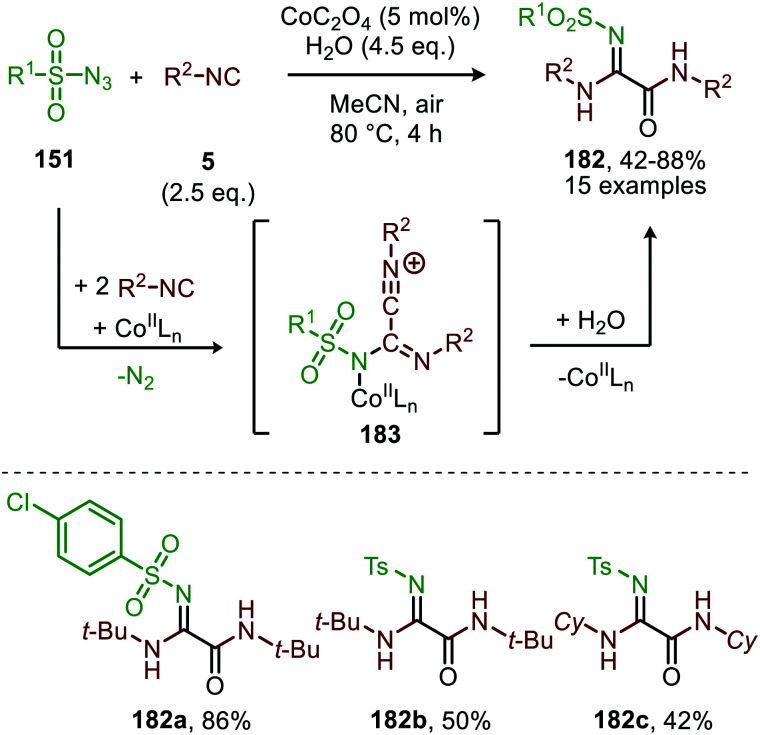
Co^II^-Catalysed nitrene transfer to isocyanides (5) in the synthesis of sulfonylamidinyl amides (182).

The same group reported the synthesis of sulfonylamidyl amides (182) from sulfonyl azides (151) and two equivalents of isocyanide *via* a similar Co^II^-catalysed approach in the presence of water (Scheme 49).^86^ The nitrene transfer proceeds *via* Co^III^-nitrene radical intermediate (178) and furnishes metallated carbodiimide (180) after 1,1-migratory insertion (Scheme 47). Subsequently, Co^II^-carbodiimide complex (180) (Scheme 47) is attacked by an additional isocyanide molecule (5) to furnish nitrilium ion intermediate (183) (Scheme 49). This cationic intermediate is then trapped by water, which after tautomerisation result in the sulfonylamidyl amides (182). The reaction tolerated a broad range of substituted aromatic- and aliphatic isocyanides to afford sulfonylamidinyl amides (182) in moderate to good yield.

**Scheme 50:**
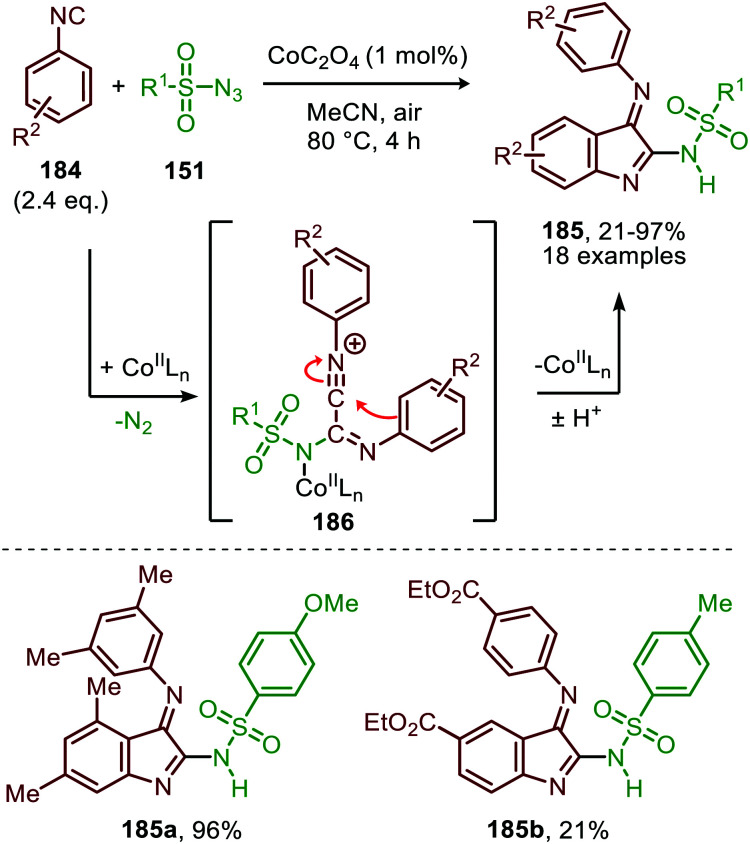
Co^II^-Catalysed nitrene transfer to aryl isocyanides (**184**) in the synthesis of 3-imino-3*H*-indole scaffolds 185.

Interestingly, when aromatic isocyanides are used in the absence of water, a completely different reaction product was obtained (Scheme 50).^86^ The reaction between sulfonyl azide (151) and aromatic isocyanides (184) results in Co^II^-carbodiimide complex (180) (Scheme 47), which in turn is attacked by a second isocyanide to furnish nitrilium ion 186 (Scheme 50). Subsequent intramolecular electrophilic aromatic substitution affords 3-imino-3*H*-indole derivatives 185 in low to excellent yields. Electron poor aryl isocyanides drastically lower the yield due to their poor performance in the electrophilic aromatic substitution step from nitrillium intermediate 186.

**Scheme 51:**
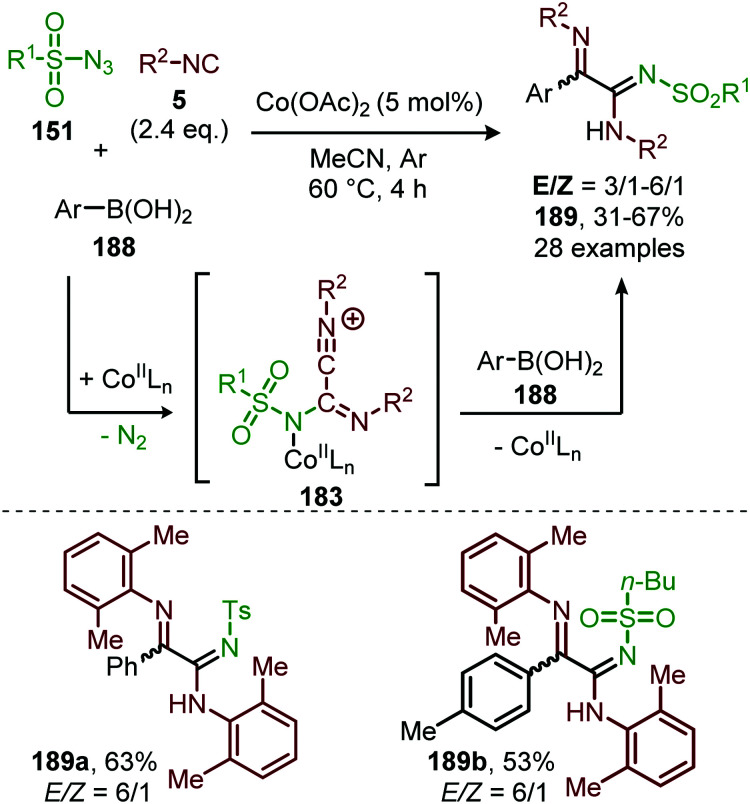
Co^II^-Catalysed four-component coupling between isocyanides (5), sulfonyl azides (151) and aryl boronic acids (188) towards amidinyl imines (189).

In 2018, Ji and co-workers developed a similar Co^II^-catalysed strategy to arrive at amidinyl imine derivatives (189) in low to moderate yields. For this they combined sulfonyl azides (151), isocyanides (5) and boronic acids (188) in one-pot (Scheme 51).^87^ The transformation commences with carbodiimide formation according to the mechanism in Scheme 47 and complies with route I of our general mechanism (Scheme 2). Again, the metallated carbodiimide (180) (Scheme 47) is attacked by a second isocyanide to furnish nitrillium intermediate (183) (Scheme 51). 183 is then trapped with boronic acids (188) to arrive at amidinyl imine 189. Bis-*o*-substituted aromatic isocyanides were tolerated in the reaction (51–67%), however, aliphatic isocyanides did not show any conversion. Only aryl boronic acids (188) served effectively as coupling partner determining the observed *E*/*Z*-ratio. The selectivity of the reaction depends on the position of the substituent on the aryl ring of boronic acid. Typically, *ortho*-groups achieve an *E*/*Z* selectivity of 3/1, whereas *para*- or *meta*-substituents afforded a better *E*/*Z* selectivity of 6/1.

**Scheme 52:**
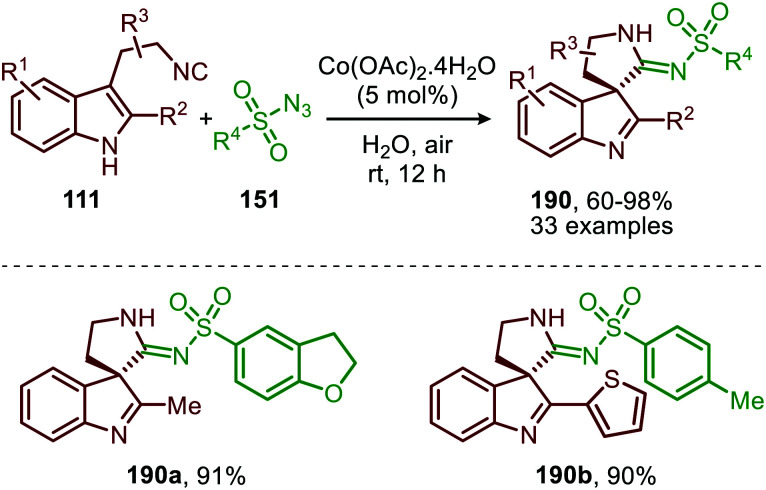
Co^II^-Catalysed nitrene transfer of sulfonyl azides (151) to tryptamine derived isocyanides (111).

Spiroindolenines (190) could also be synthesized from sulfonyl azides (151) and functionalized tryptamine-derived isocyanides (111) in good to excellent yield (Scheme 52).^88^ A major advantage is that the reaction could be performed in water. Arguably, this makes the reaction attractive from a sustainable point of view. Noteworthy is that a substituent on the C2-position of the indole is essential in order to isolate the desired product 190. However, when a hydrogen is present at this position the corresponding indoline was isolated, as a result of *in situ* imine reduction with NaBH_4_ of the obtained indolenine (190). Arene- as well as alkanesulfonyl azides (151) were suitable nitrene precursors. The authors propose the reaction to proceed *via* Co^III^/nitrene radical species (178) (Scheme 47), where bis-isocyanide Co^II^-complex (176) initiates the formation of the Co^III^-nitrene intermediate (Scheme 47).

**Scheme 53:**
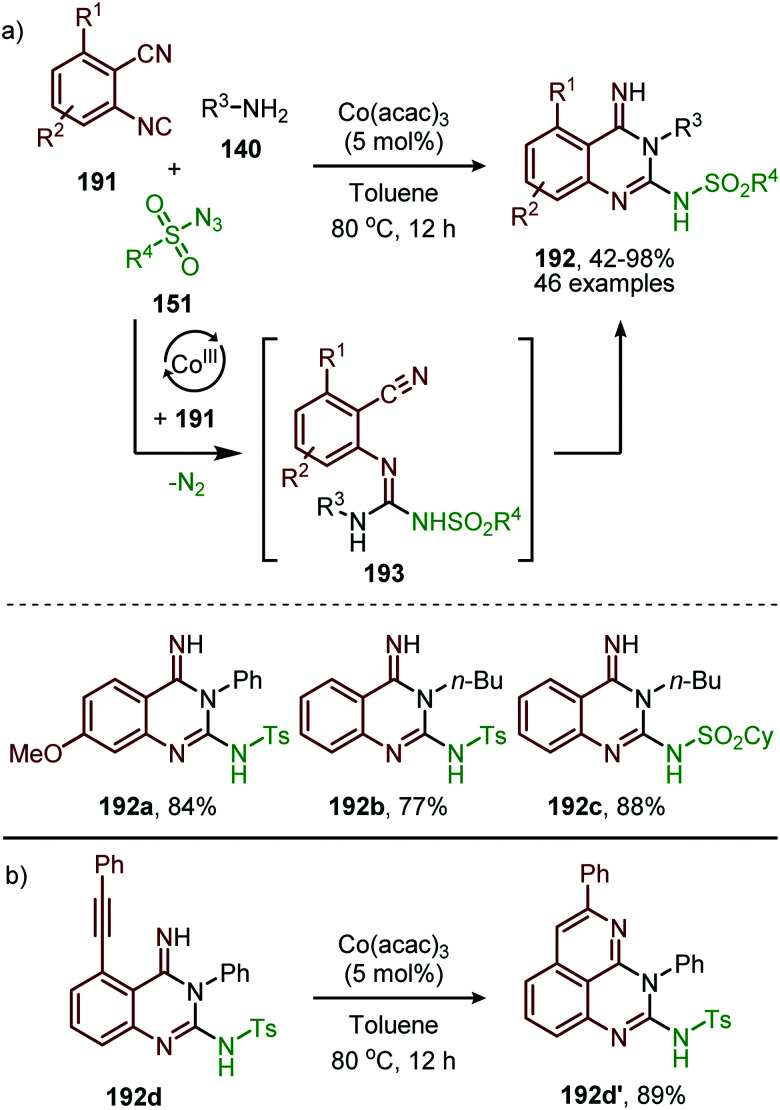
(a) Co^III^-Catalysed three-component coupling between 2-cyanoarylisocyanides (191), sulfonyl azides (151), and amines (140) towards quinazolin-4(3*H*)-imines (192). (b) When R^1^ is an alkyne *in situ* cyclization occurs forming a fused quinazoline derivative 192d′.

In recent work of this group, the Co^II^-catalysed nitrene transfer of sulfonyl azides (151) to cyanoarylisocyanides was applied for the formation of quinazolin-4(3*H*)-imines (192) (Scheme 53a).^89^ The cascade process commences with Co^II^-catalysed formation of the corresponding carbodiimide following the mechanism illustrated in Scheme 47. Subsequent nucleophilic addition of aromatic as well as aliphatic amines (140) furnished the corresponding *N*-sulfonyl guanidines (193). Finally, ring closure on the aromatic nitrile results in quinazolin-4(3*H*)-imines (192) in moderate to excellent yields. Interestingly, when an alkyne as R^1^-substitutent is present on substrate 191, a second cyclisation could occur to obtain product 192d′ (Scheme 53b).

#### Nickel

**Scheme 54:**
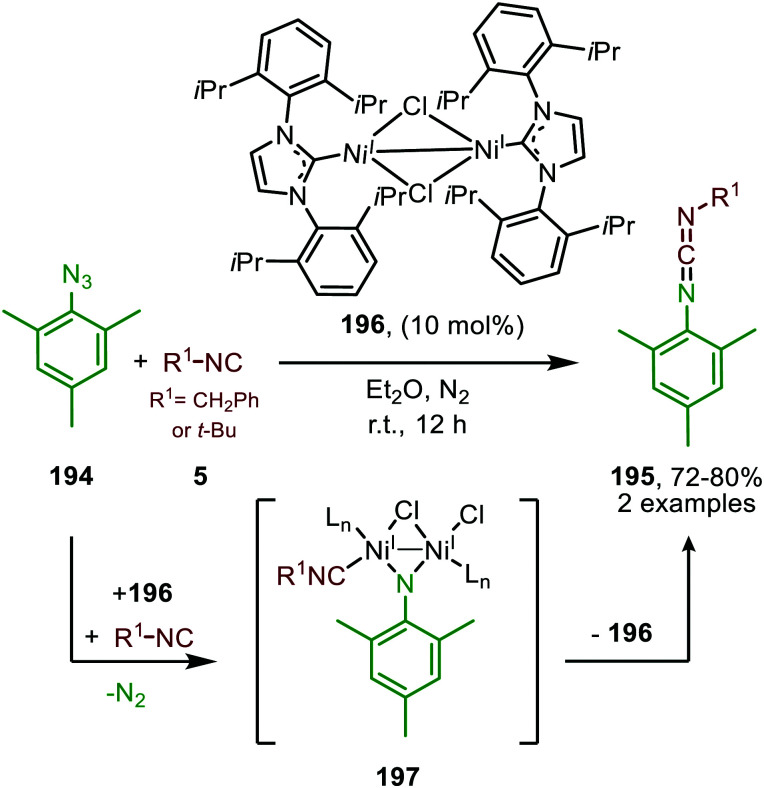
Ni^I^-Catalysed imidoylation of nitrene derived from mesityl azide *via* bridged-di-nickel imido species 197.

Also, nickel received some attention though only to form carbodiimides but not involving *in situ* post transformations with nucleophiles. The di-nickel^II^ bridged complex 196 featuring IPr ligands was developed by Hillhouse and co-workers for the synthesis of unsymmetrical carbodiimides 195 (Scheme 54).^90^ The scope of the reaction was not extensively studied and solely benzyl isocyanide and *tert*-butyl isocyanide were employed as input examples. The proposed mechanism follows either route I or II of the cycle presented in Scheme 2. One notable rare aspect is that the active intermediate is considered to be di-Ni^I^-μ-imido species 197 rather than a monometallic one. The authors do not demonstrate the use of additional azides beyond mesityl azide or *in situ* post transformation of the carbodiimide obtained.

**Scheme 55:**
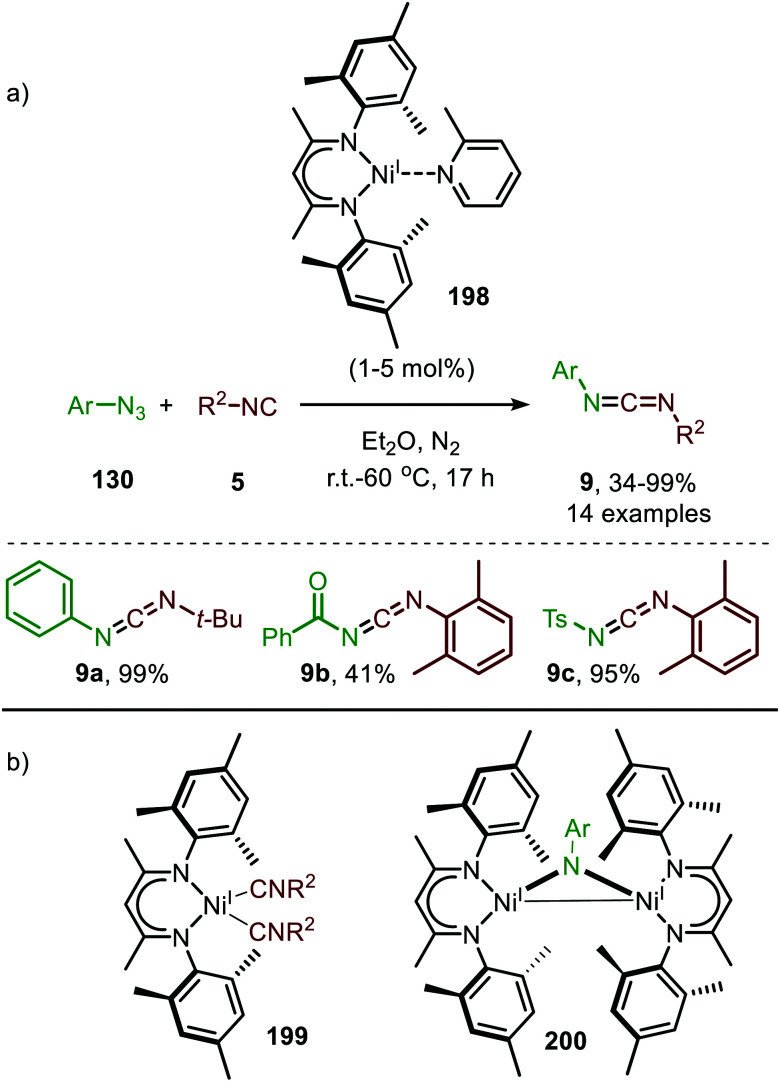
(a) Ni^I^-Catalysed imidoylation of aromatic azides, and (b) proposed active species in the nitrene transfer to isocyanides.

Warren and co-workers developed another Ni^I^-catalyst (198) for the nitrene transfer reaction to isocyanides (Scheme 55).^91^ This β-diketiminato Ni^I^-catalyst with picoline spectator ligand (198) proved highly active at relatively low catalyst loading. Under optimal conditions the reaction of aryl azides (130) afforded carbodiimides (9) in good yields. However, reaction with electron poor azides such as tosyl azide, furnished the product 9 in lower yield. The authors assume that the lower electron density of these organoazides hampers entering the coordination sphere of the metal centre as it competes with the more electron-rich isocyanide. In addition to aromatic azides, homobenzylic azide and benzoyl azide were successfully employed. Turning attention to the isocyanide substrate scope, only *tert*-butyl isocyanide and 2,6-dimethylphenyl isocyanide were tested. Moreover, the carbodiimides (9) were not always isolated and the reaction was carried out in a glove box. Notably, the authors synthesized β-diketiminato bis-(isocyanide)-Ni^I^ complex 199 (Scheme 55b) and imido bridged di-Ni^I^-imido complex (200) derived from β-diketiminato Ni^I^-catalyst (Scheme 55b). Stoichiometric experiments with complex 199 and azide (130), and with complex 200 and isocyanide (5), both afforded the carbodiimide (9), indicating that both Ni-species 199 and 200 could be active intermediates within the catalytic cycle. Unfortunately, the authors do not further comment on the exact mechanism of carbodiimide formation.

#### Zirconium

**Scheme 56:**
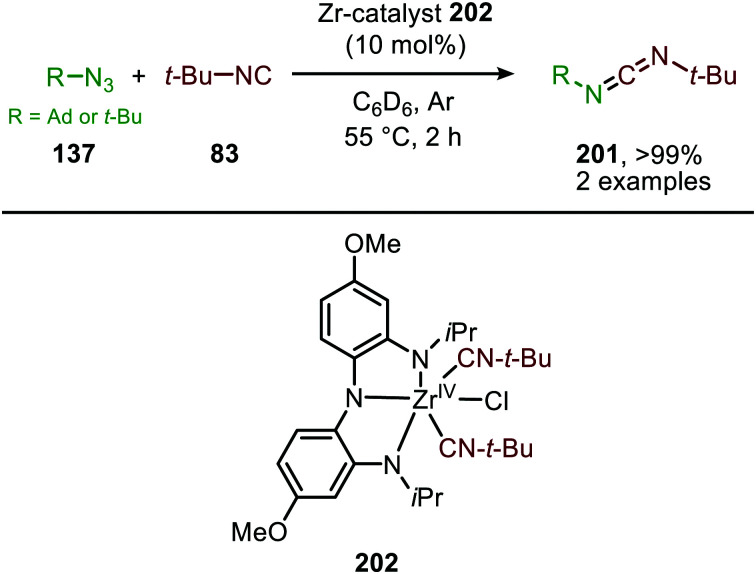
Zr^IV^-Catalysed nitrene transfer to *t*-BuNC using a redox active ligand.

Arriving at the early row V transition metals, Heyduk *et al.* described a rare Zr^IV^-catalysed nitrene transfer to isocyanides using a pincer complex 202. The bis(2-iso-propylamido-4-methoxyphenyl)amide ([NNN^cat^])^3−^) acting as a redox-active ligand is key here (Scheme 56).^92^ The redox activity of this ligand, cycling between the catecholate and quinonate form, allows the zirconium metal centre to remain in its more stable d^0^ – oxidation state. According to their scope studies the reaction with *tert*-butyl isocyanide tolerates bulky adamantyl azide and *tert*-butyl azide, furnishing carbodiimides 201 quantitatively. An inert Zr^IV^-imido dimer is observed when aromatic azides are employed. The proposed mechanism is generally in accordance with route I (Scheme 2), however there are important differences when zooming in as the ligand is actively involved here. Zr imido complex is formed *via* reaction with azide. Two electrons of the ligand are used for this process providing the quinonate ligand form. 1,1-Migratory insertion of coordinated isocyanide affords an η^2^- coordinated metallacycle similar to 13 (Scheme 3a). Subsequently, carbodiimide 201 is formed *via* a reductive elimination of the corresponding η^2^-carbodiimide Zr-complex, involving a two electron reduction of the quinonate form of the redox-active ligand into its catecholate rather than the metal centre. In principle, the nitrene fragment in early-transition-metal complexes is considered nucleophilic. Therefore, nitrene transfer to an isocyanide, which is intrinsically nucleophilic, can only occur after it is activated by the electrophilic metal centre.^92^ This in contrast with mid- to late TM processes where the nitrene fragment is considered to be electrophilic.^92^ Therefore, depending on the substrates and TM employed, different type of reactivity can be observed.

#### Niobium

**Scheme 57:**
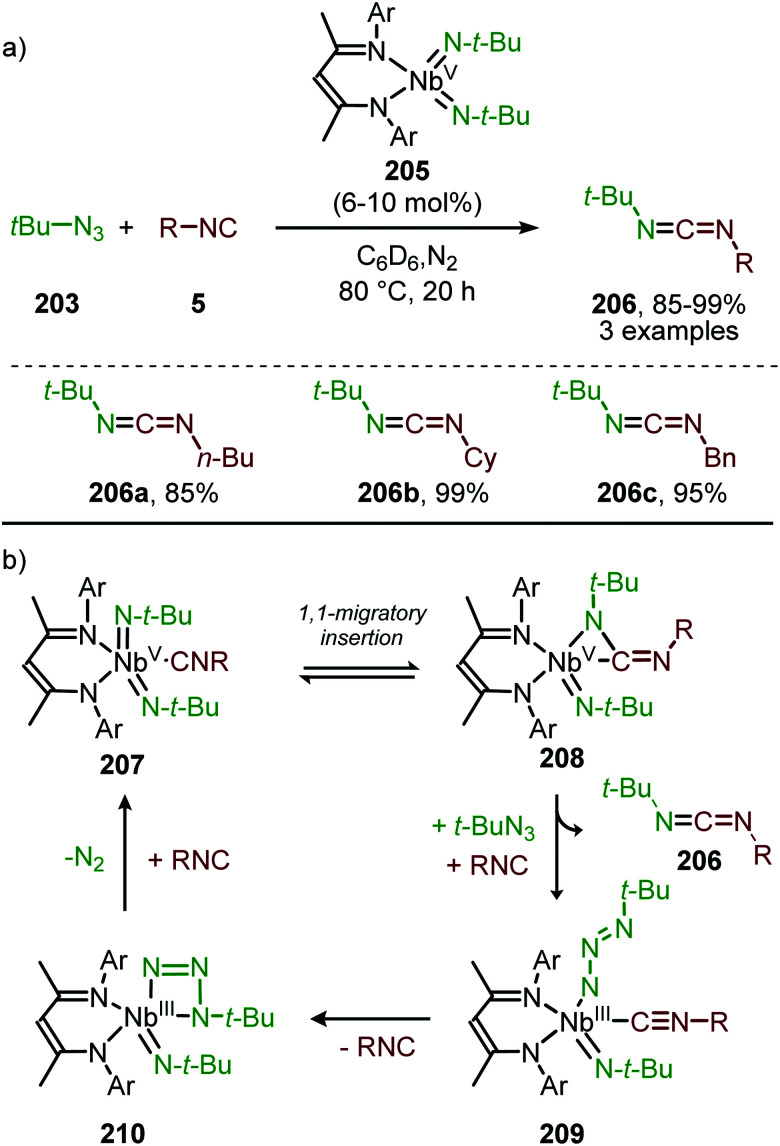
(a) Nb^V^-Catalysed imidoylation of nitrenes towards carbodiimides (206). (b) Postulated intermediates involved in the reaction mechanism.

Neighboring to Zr in row V is middle transition metal Nb. It can catalyse the nitrene transfer to isocyanides as is demonstrated by Arnold *et al.* (Scheme 57a).^93^ They report the use of bis-imido Nb-complex 205 in the nitrene transfer to cyclohexyl isocyanide or *tert*-butyl isocyanide in good to excellent yield. An extensive scope study, however, was not provided. The proposed mechanism is similar to route II of the general cycle in Scheme 2. Nb cycles in between oxidation state V and III in the catalytic cycle. First the isocyanide fragment coordinates to bis-imido complex 205, affording Nb-complex 207 (Scheme 57b), which is in equilibrium with η^2^- coordinated metallacycle 208. The latter undergoes reductive elimination and releases the carbodiimide 206, concomitantly coordinating azide and isocyanide forming Nb complex 209. In this step the oxidation state reduces from V to III. This is followed by dissociation of the isocyanide and formation of four-center intermediate 210. Subsequently, bis-imido complex 207 is regenerated with the release of N_2_ and coordination of the isocyanide. The authors observed that the use of aromatic isocyanides resulted in a nitrene metathesis instead of affording the corresponding carbodiimide. This *scrambling* process resembles the earlier described metathesis process in the Ti-catalysed nitrene transfer to isocyanides (Scheme 41).

#### Rhodium

**Scheme 58:**
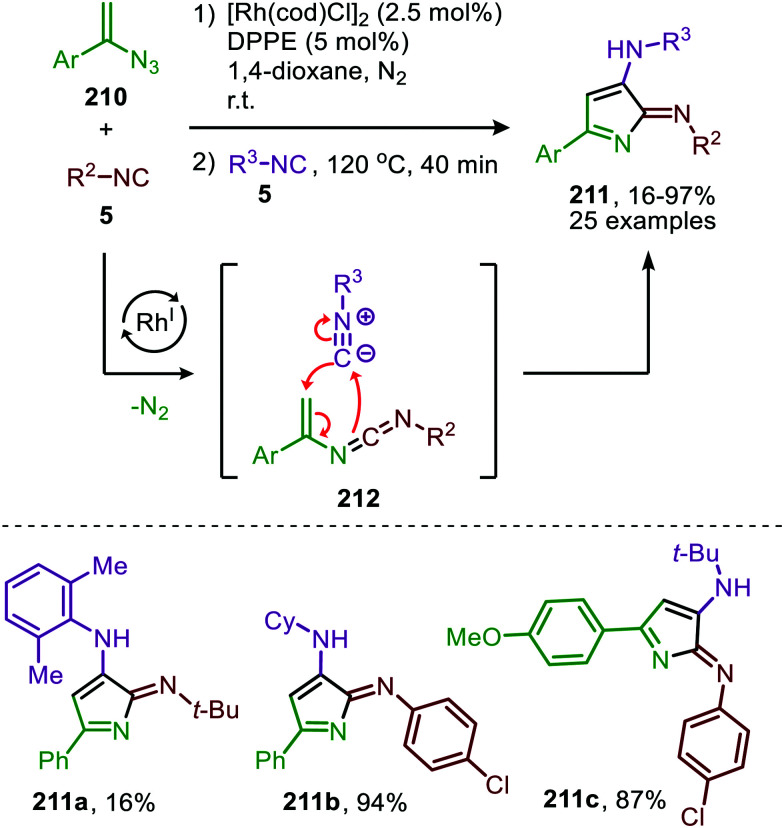
One-pot Rh^I^-catalysed nitrene transfer to isocyanide (5) followed by a [4+1]-cycloaddition furnishing 3-amino-2*H*-pyrrol-2-imines (211).

Rhodium is a reoccurring catalyst for the imidoylation and carbonylation of carbenes as well as in the imidoylation of nitrenes. This is often followed by *in situ* transformation of the resulting corresponding carbodiimide (9) making it especially powerful for organic synthesis. Zhang *et al.* demonstrated this *via* a Rh^I^-catalysed nitrene transfer to isocyanides followed by a [4+1]-cycloaddition with an additional isocyanide. This one-pot operation affords 3-amino-2*H*-pyrrol-2-imines (211) (Scheme 58).^94^ The reaction is receptive to an array of aryl substituted vinyl azides (210) (69–96%). In addition, aromatic isocyanides as well as aliphatic isocyanides were accepted in the formation of carbodiimide 212 and the subsequent [4+1]-cycloaddition. However, using aliphatic isocyanides in the first step (carbodiimide formation) furnished the product either in trace amount or low yields, indicating that aromatic isocyanides tend to work better for the nitrene transfer. A similar trend was observed in the Rh-catalysed carbene transfer to isocyanides, where aromatic isocyanides were superior to aliphatic isocyanides (*vide supra*). The presumed mechanism is initiated by generating a carbodiimide according to either route I or II of the general catalytic cycle (Scheme 2). Subsequently, by adding another isocyanide and heating to 120 °C the carbodiimide undergoes a thermal [4+1]-cycloaddition forming 3-amino-2*H*-pyrrol-2-imines (211).

**Scheme 59:**
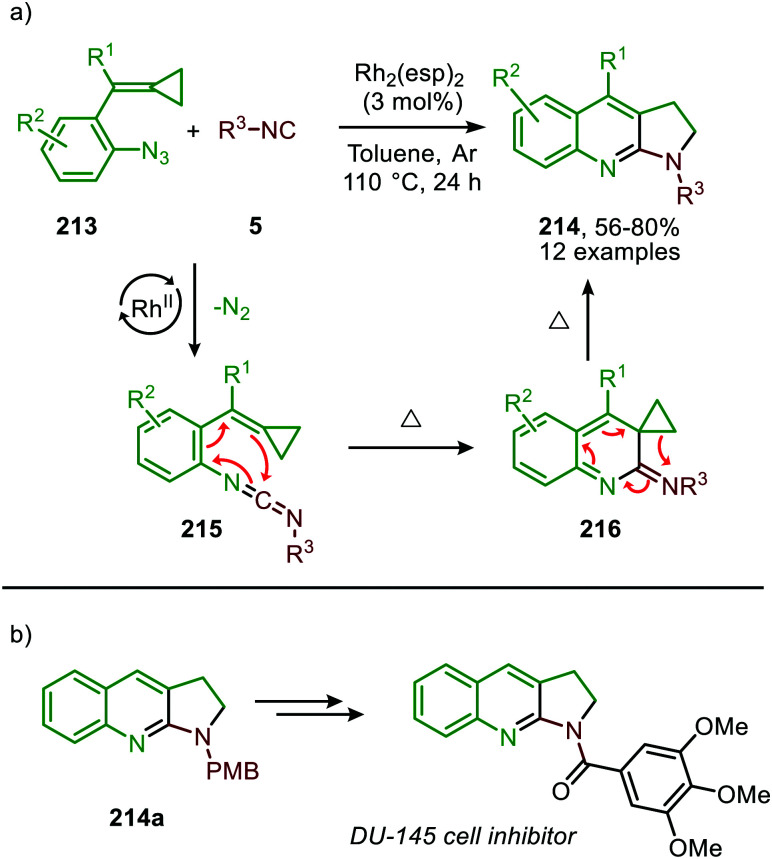
(a) Rh^II^-Catalysed nitrene transfer/electrocyclisation/thermal rearrangement cascade forming 2,3-dihydropyrrolo[2,3-*b*]-quinolines (214). esp = α,α,α′,α′-tetramethyl-1,3-benzenedipropionic acid. (b) Access to DU-145 cell inhibitor from product 214a.

Additionally, Shi *et al.* reported a Rh^II^-catalysed cascade process towards 2,3-dihydropyrrolo[2,3-*b*]quinolines (214) based on isocyanides and functionalised aromatic azides (213) (Scheme 59).^95^ The reaction proceeds *via* carbodiimide 215, which undergoes a 6π-electrocyclisation. The thus formed intermediate 216 furnishes, upon thermal rearrangement, the desired product 214. Both aromatic-, benzylic and secondary- or tertiary aliphatic isocyanides were tolerated in the tandem reaction. The authors propose a mechanism, which is in accordance with either route I or II of the mechanism depicted in Scheme 2. Although this Rh^II^ catalyst is known to participate in radical reactions involving distinct nitrene species,^96^ a radical mechanism was ruled out based on radical trapping experiments using TEMPO as radical scavenger. Noteworthy, the authors provided a facile method to synthesise a DU-145 cell inhibitor, starting from product 214a (Scheme 59b).

**Scheme 60:**
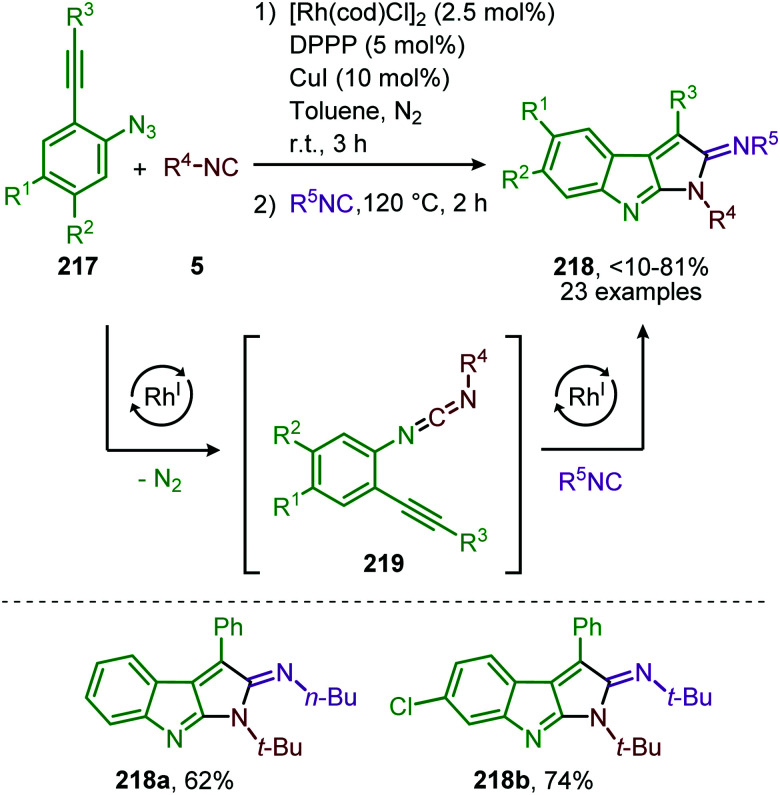
One-pot Rh^I^-catalysed nitrene transfer to isocyanides followed by isocyanide addition and aza-Pauson-Khand type cyclisation on carbodiimide 219.

Additionally, a self-relay^97^ Rh^I^-catalysed transformation towards pyrrolo[2,3-*b*]indol-2(1*H*)-imine scaffolds 218 was developed by Zhang and co-workers (Scheme 60).^98^ The reaction proceeds *via* a Rh^I^-catalysed nitrene transfer to isocyanides furnishing carbodiimide 219. By addition of isocyanide to the same reaction vessel and heating to 120 °C an aza-Pauson-Khand type cyclisation occurs yielding pyrroloindoles 218. In general, for the nitrene transfer step, linear primary-, cyclic secondary and tertiary aliphatic isocyanides were able to form the product 218 in good yields. When aromatic isocyanides were employed in the carbodiimide formation product formation occurs, albeit less efficiently (47–66%).

**Scheme 61:**
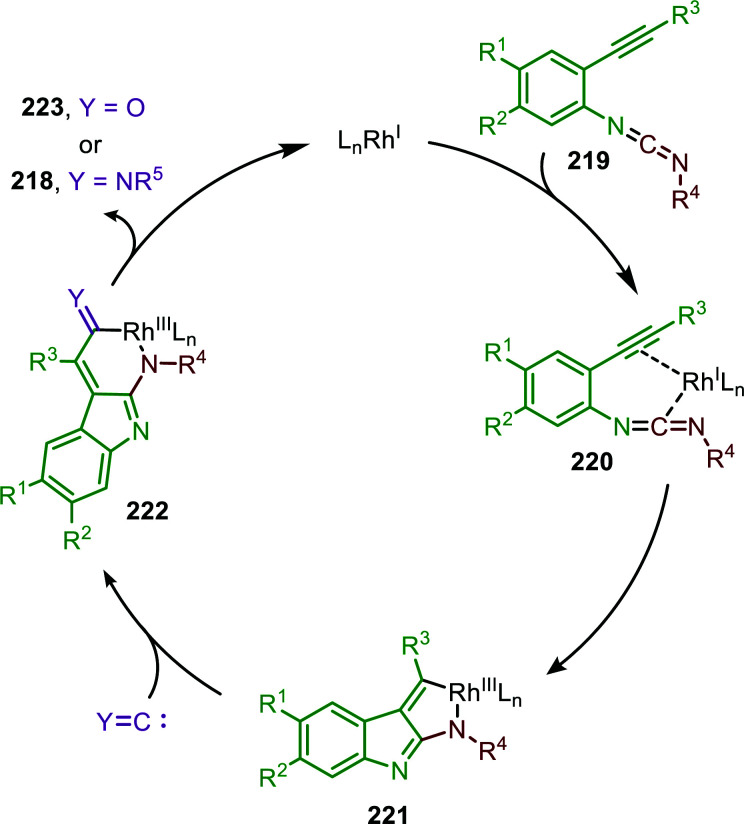
Mechanism of the Rh^I^-catalysed relay-cycle involving oxidative cyclometallation of carbodiimide 219 into 221.

Based on the provided mechanistic data the formation of carbodiimide 219 presumably proceeds according to either route I or II in the general mechanism in Scheme 2. The cyclisation commences with coordination of intermediate 219 to a Rh^I^-species (Scheme 61). After formation of π-complex 220 an oxidative cyclisation occurs, furnishing cyclometallated Rh^III^-species 221. Subsequently, an isocyanide can insert into the Rh-C bond, forming metallacycle 222, followed by reductive elimination to afford product 218. The authors observe that the addition of Cu^I^I improved the yield of the reaction. However, in their proposed mechanism they do not consider its catalytic role in either the nitrene transfer or the aza-Pauson-Khand type cyclisation.

**Scheme 62:**
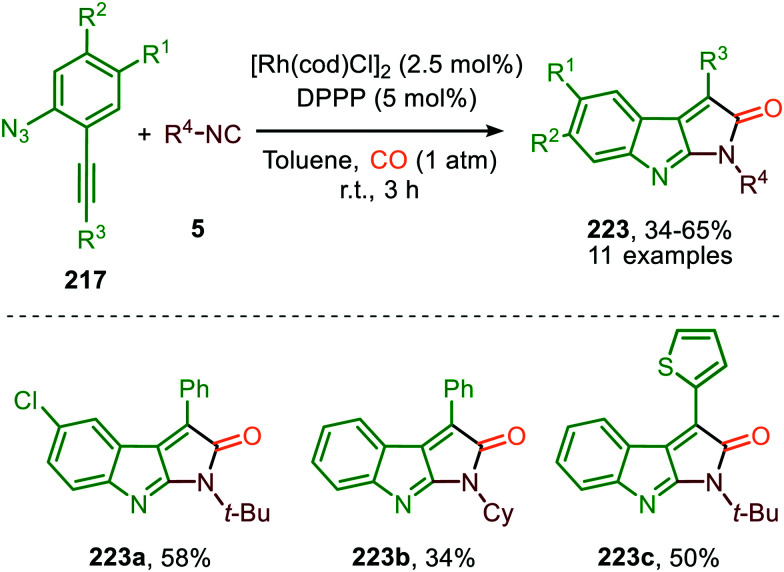
Tandem Rh^I^-catalysed nitrene transfer to isocyanides and Pauson-Khand type cyclisation on carbodiimide 219.

Instead of utilizing an isocyanide in the aza-Pauson-Khand reaction, CO could be used and present at the start of the reaction (reaction atmosphere) selectively forming scaffolds 223 in moderate yields (Scheme 62).^98^ Presence of both isocyanide and CO at the same time implies that in this catalytic system, the isocyanide reacts faster in the carbodiimide formation step than its isoelectronic counterpart CO. Noteworthy is that the Cu^I^-salt was omitted in this case.

**Scheme 63:**
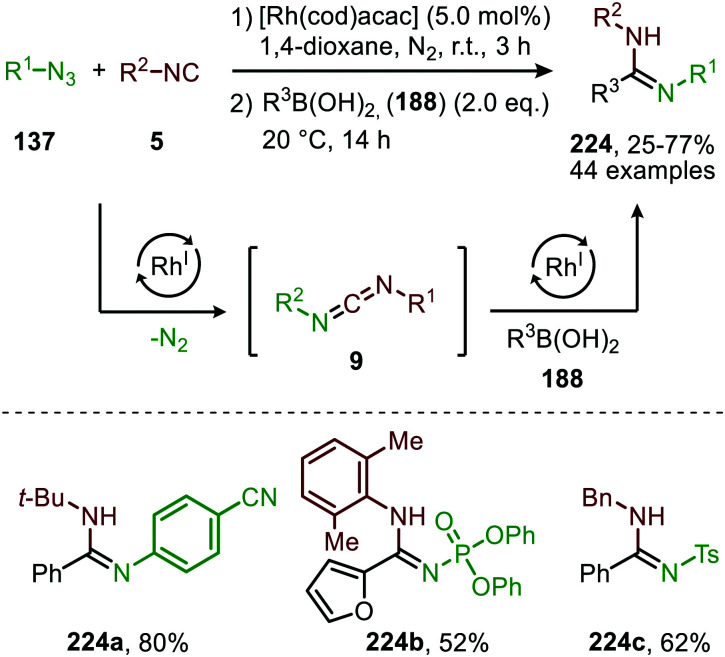
Self-relay Rh^I^-catalysed formation of amidines 224 from azides (137), isocyanides (5) and aryl- or alkenyl boronic acids (188).

An additional self-relay Rh^I^-catalysed cross-coupling of azides (137), isocyanides (5) and aryl- or alkenyl boronic acids (188) to access highly functionalized amidines (224) (Scheme 63) was reported by the group of Zhang.^99^ The reaction accepted a variety of electron poor nitrene precursors, such as sulfonyl-, phosphoryl-, and acyl azides (137). On the other hand, the isocyanide scope was limited to *tert*-butyl isocyanide, cyclohexyl isocyanide, benzyl isocyanide and 2,6-xylyl isocyanide. Presumably, the mechanism for *in situ* carbodiimide (9) formation operates *via* either route I or II of the generalized mechanism (Scheme 2). The second catalytic cycle is initiated by addition of boronic acid 188 providing transmetallation of the aryl or alkenyl moiety to the Rh-centre, followed by insertion in the C=N of the *in situ* generated carbodiimide. This generates a *N*-bound Rh^I^-complex, which undergoes protonolysis with another boronic acid to furnish a rhodium boronate complex accompanied by release of the desired amidine 224. The rhodium boronate complex regenerates an aryl or alkenyl rhodium complex through β-elimination to close the catalytic cycle.

#### Palladium

Pd-Catalysis holds a pivotal position in the imidoylation reaction of both carbenes and nitrenes. Although the Pd-catalysed imidoylation of nitrenes was covered in our broader review of 2020 on Pd-catalysed cross-coupling reactions with isocyanide insertion^11*b*^ here the focus is on the mechanistic implications.

**Scheme 64:**
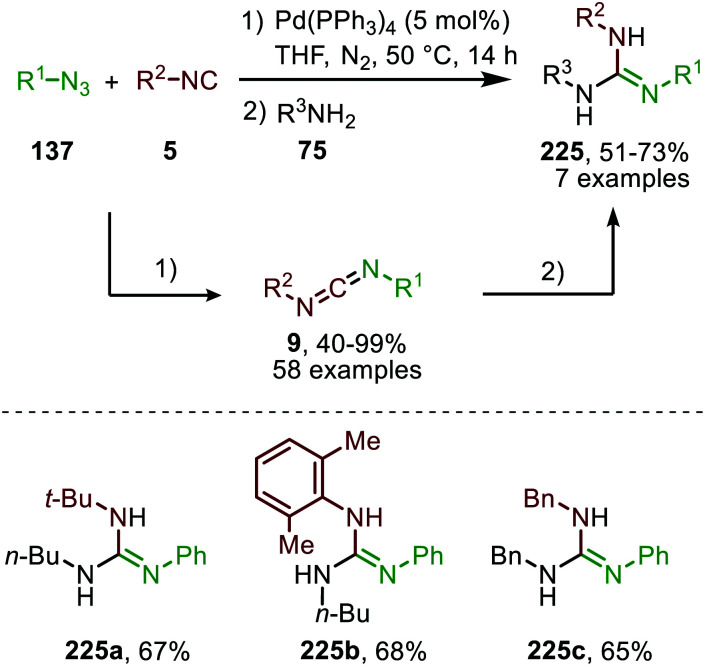
One-pot Pd^0^-catalysed synthesis of carbodiimides (9) *via* imidoylation of azides (137) followed by addition reaction of amines (75) providing guanidines (225).

Zhang and co-workers demonstrated the use of Pd^0^ as catalyst in the nitrene transfer to isocyanides and isolated numerous non-symmetrical carbodiimides (9) in moderate to excellent yield (Scheme 64).^100^ In addition, they extended their method to the one-pot synthesis of guanidines (225) by trapping the carbodiimides (9) with primary aromatic- and aliphatic amines. The reaction of aromatic azides in combination with a range of aliphatic and aromatic isocyanides was successful, however the carbodiimide yield dropped when benzylic- or aliphatic azides were evaluated. The authors postulate a mechanism, which is in accordance with either route I or II in Scheme 2. Unfortunately, no in depth mechanistic studies were provided to support their proposed mechanism.

**Scheme 65:**
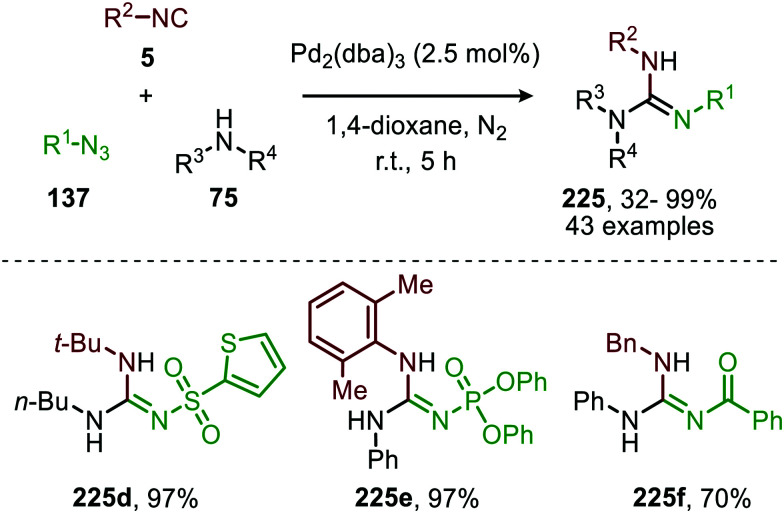
Tandem Pd^0^-catalysed synthesis of carbodiimides *via* imidoylation of azides (136) followed by addition reaction of amines (75) forming guanidines (225).

In continuation of their work, they also developed a Pd-catalysed three-component coupling of acyl-, sulfonyl- or phosphoryl azides (137), isocyanides (5), and primary- and secondary amines (75) towards guanidines (225) (Scheme 65).^101^ Electron poor azides were transformed towards the corresponding *N*-sulfonyl-, *N*-acyl-, and *N*-phosphoryl guanidines (225) in moderate to excellent yield. However, lower yields were obtained when benzyl isocyanide or aromatic isocyanides were employed. The amine scope proved to be broad and overall the reaction displayed a high functional group tolerance. The proposed mechanism is in accordance with route I or II of general catalytic cycle (Scheme 2). However, the authors did not perform any mechanistic or computational studies to provide support.

**Scheme 66:**
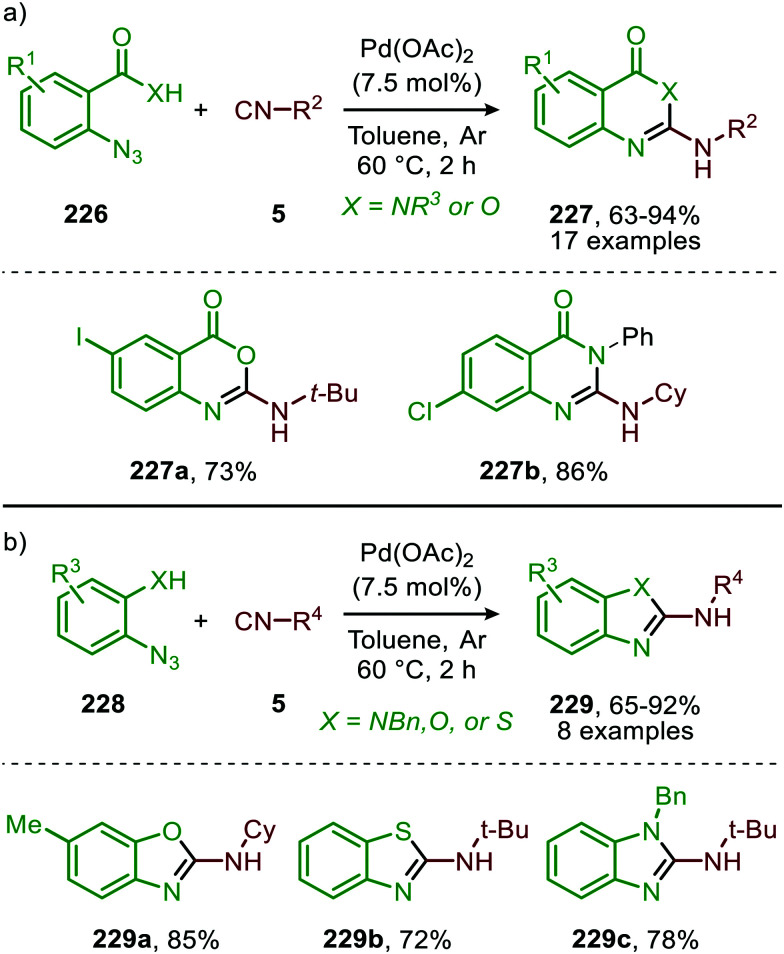
Tandem Pd^II^-catalysed synthesis of carbodiimides *via* nitrene transfer to isocyanides followed by intramolecular addition on the *in situ* formed carbodiimide providing (a) benzoxazinones (227, X = O), quinazolinones (227, X = NR) and (b) benzimidazoles (229, X = NR), benzoxazoles (229, X = O), and benzothiazoles (229, X = S).

In addition to the synthesis of guanidines (225) *via* initial Pd-catalysed nitrene transfer to isocyanides, Pardansani and co-workers extended the Pd-catalysed transformation to the synthesis of numerous *N*-heterocycles *via* combination with an intra- rather than an intermolecular addition reaction on carbodiimides (Scheme 66).^25^ They studied the nitrene transfer of aryl azides (226, 228) (Schemes 66a and b) to isocyanides (5) followed by an intramolecular nucleophilic addition on the *in situ* formed carbodiimide moiety to arrive at benzoxazinones (X = O), quinazolinones (X = NR) (227) (Scheme 66a), benzimidazoles (X = NR), benzoxazoles (X = O), and benzothiazoles (X = S) (229) (Scheme 66b), which could be obtained in good to excellent yield.

The authors conduct an extensive study to determine the substrate scope and both transformations display a high tolerance with respect to the azide 226 and 228 used. Moreover, the reactions with secondary- and tertiary aliphatic isocyanides, benzyl isocyanide, and α-isocyano esters furnished the products in good yield. Even notoriously unstable phenyl isocyanide reacted smoothly in the synthesis, affording the corresponding benzoxazole 229 in 65% yield. Unfortunately, aromatic isocyanides were not tolerated in the synthesis of benzoxazinones (227, X = O) and quinazolinones (227, X = NR). The authors provided some mechanistic insights for the synthesis of diverse *N*-heterocycles *via* experimental observations and extensive DFT studies. The postulated order of events for carbodiimide formation is in accordance with route I of the general mechanism in Scheme 2, with nitrogen extrusion as the rate-determining step. Interestingly, the authors could not find an energy minimum on the potential energy surface (PES) for the η^2^-metallaaziridine intermediate (13) (Scheme 3a), which is a commonly proposed intermediate after 1,1-migratory insertion of the isocyanide to the nitrene fragment. Instead, the authors found a transition state resembling the η^2^-metallaaziridine intermediate (13) (Scheme 3a), indicating that 1,1-migratory insertion towards the η^2^-metallated carbodiimide (12, Scheme 3a) can proceed in an asynchronous concerted fashion. It is reported in literature that metallaziridines can be stable intermediates for early TMs.^102–104^ However, such a metalla-aziridine intermediate has not been isolated in the case of Pd.

**Scheme 67:**
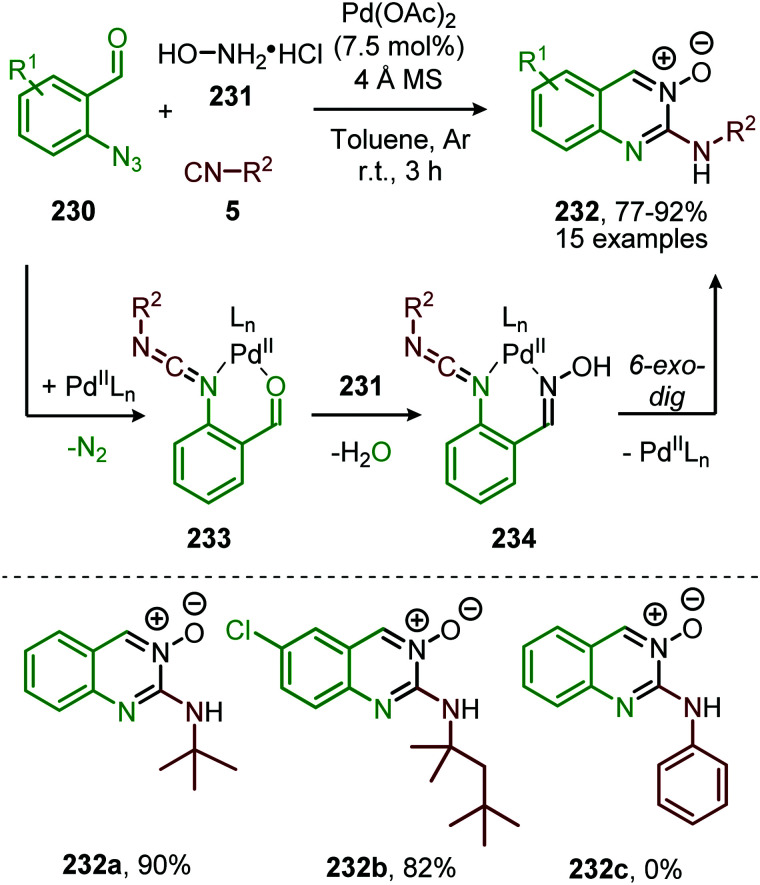
Three-component synthesis of quinazoline-3-oxides (232) *via* self-relay Pd^II^-catalysis involving initial nitrene transfer to isocyanides followed by Pd-catalysed *6-exo-dig* cyclisation.

Sawant *et al.* extended the redox neutral Pd^II^-catalysed nitrene transfer to a three-component reaction of 2-azido-benzaldehydes (230), isocyanides (5) and hydroxylamine hydrochloride (231) for the synthesis of quinazoline-3-oxides (232) in good to excellent yields (Scheme 67).^105^

Within this tandem catalytic process, the isocyanide scope is limited towards tertiary aliphatic isocyanides, such as in scaffolds 232a and b (Scheme 67). It is noteworthy to mention that the reaction operates under relatively mild conditions. Based on control experiments and literature precedents the authors postulate that after nitrene transfer carbodiimide chelated Pd^II^-complex (233) (Scheme 67) is formed. Subsequent condensation of hydroxylamine (231) with the benzaldehyde of 233 furnishes Pd-oxime complex 234, which after Pd-facilitated 6-exo-dig cyclization delivers quinazoline-3-oxides (232). The proposed mechanism of carbodiimide formation follows route I of our mechanism depicted in Scheme 2.

**Scheme 68:**
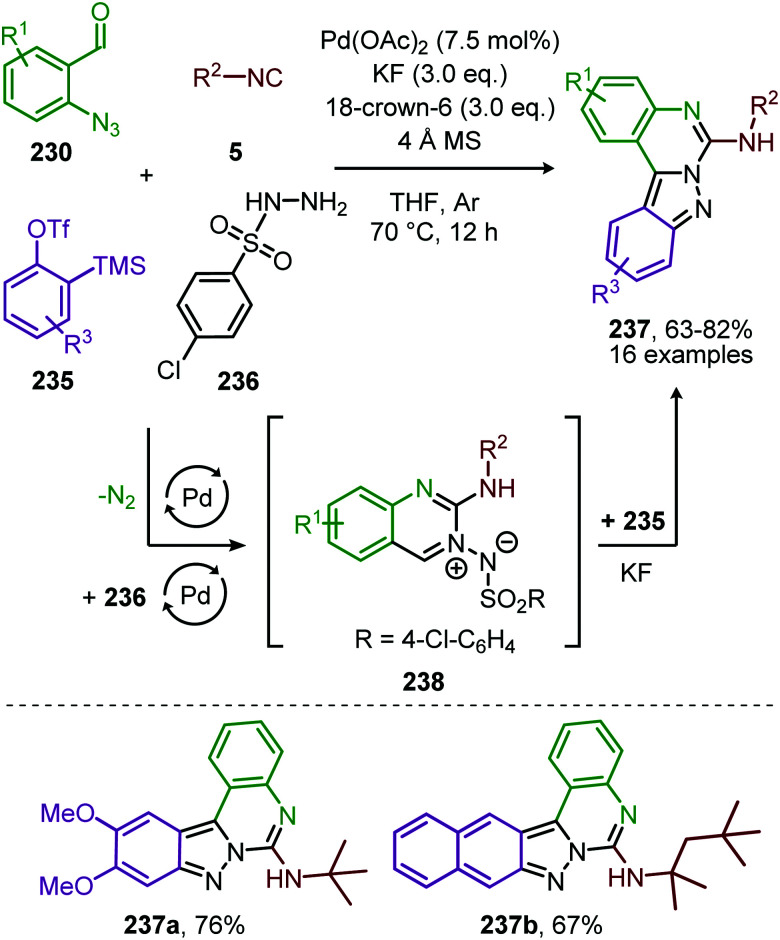
Four-component synthesis of indazolo[2,3-*c*]quinazolin-6-amines (237) *via* self-relay Pd^II^-catalysis involving initial nitrene transfer to isocyanides followed by Pd-catalysed *6-exo-dig* cyclisation.

This concept of self-relay Pd^II^-catalysed nitrene transfer followed by 6-exo-dig annulation was also applied by these authors in a four-component coupling towards fluorescent indazolo[2,3-*c*]quinazolin-6-amines (237) (Scheme 68).^106^ The transformation combines 2-azidobenzaldehydes (230), isocyanides (5) and *N*-tosylhydrazine (236) providing azomethine imine intermediate 238*via* carbodiimide intermediate 233 as described in Scheme 68. In this case *N*-sulfonylhydrazone rather than oxime is formed from benzaldehyde, which adds in an intramolecular fashion to the carbodiimide. Subsequent dipolar cycloaddition of 238 with an *in situ* generated benzynes derived from 2-trimethylsilylaryl triflates (235) and KF,^107^ results in products 237. The reaction tolerates various halogenated 2-azidobenzaldehydes 230, but the isocyanide scope was limited to tertiary isocyanides. Cycloadditions of benzyne precursors 235 were performed with electron-rich precursors and no 2-trimethylsilylaryl triflates (235) with electron-withdrawing substituents were included in this study.

**Scheme 69:**
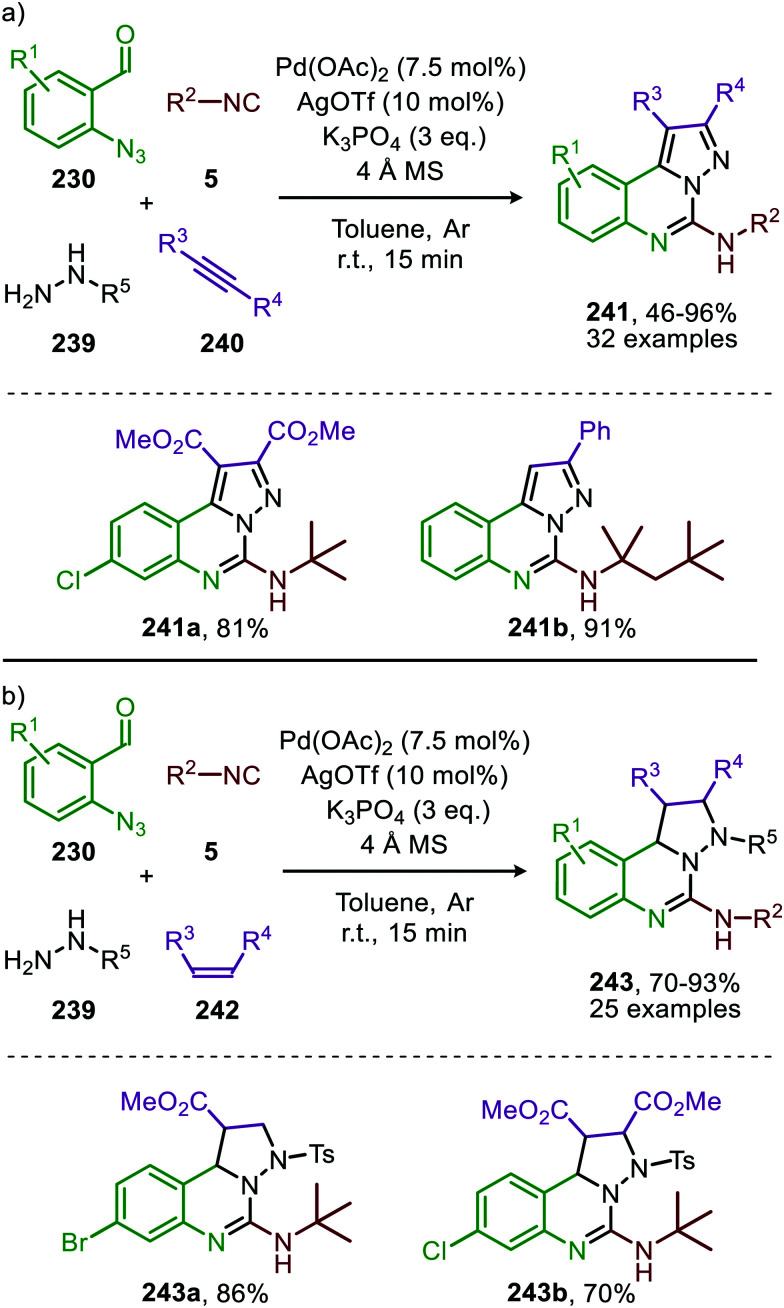
Orthogonal-relay Pd^II^/Ag^I^-catalysed four-component synthesis of (a) pyrazolo[1,5-*c*]quinazolin-5-amine (241) and; (b) tetrahydropyrazolo[1,5-*c*]quinazolin-5-amines (243).

The authors demonstrate the synthetic applicability in the formation of a high quantum yielding organic dye (237b). The dye has been used for life cell imaging of the mitochondria and cytoplasm. The authors propose a mechanism based on literature precedents and the carbodiimide is generated according to route I in the general proposed cycle of Scheme 2. Instead of benzynes also alkynes (240) (Scheme 69a) or alkenes (242) (Scheme 69b) could be used as fourth coupling partner in the cascade sequence between 2-azidobenzaldehydes (230), isocyanides (5) and *N*-tosylhydrazines (239).^108^

The transformation relies on an orthogonal relay Pd^II^/Ag^I^-catalytic process, in which Ag^I^ activates the alkyne or alkene moiety for the [3+2]-cycloaddition with azomethine imine intermediate 238 (Scheme 68), delivering pyrazolo[1,5-*c*]quinazolin-5-amines (241) (Scheme 69a) or tetrahydropyrazolo[1,5-*c*]quinazolin-5-amines (243) (Scheme 69b). Halogen- or -trifluoromethyl substituted 2-azidobenzaldehydes (230) reacted smoothly to afford product 241 or 243 in good to excellent yield. In accordance with previous methodology, the isocyanide scope was limited to tertiary aliphatic isocyanides. In addition, numerous *N*-sulfonyl hydrazines 239 were accepted a well as acyl hydrazines (**239**). Notable is that electron-deficient alkynes or alkenes allow for a successful [3+2]-cycloaddition with azomethine imine intermediate 238 (Scheme 68). The Pd-catalysed formation of carbodiimide intermediate 9 complies with route I (Scheme 2). Noteworthy is that various products (241) display anti-cancer activity with derivative 241a as the most effective according to the essay of the authors.

**Scheme 70:**
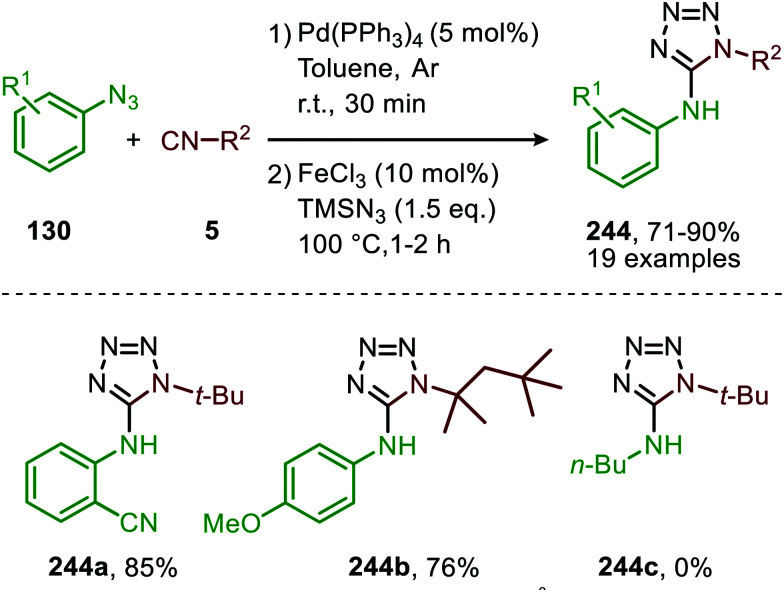
Tetrazole (244) synthesis *via* one-pot Pd^0^-catalysed formation of carbodiimides followed by an FeCl_3_-catalysed azide addition to carbodiimide.

Finally, these authors also developed a sequential Pd^0^-catalysed azide-isocyanide coupling into carbodiimide followed by a Fe^III^-catalysed formal [3+2]-cycloaddition with trimethylsilylazide yielding 5-arylaminotetrazoles (244) in good to excellent yield (Scheme 70).^109^ This one-pot transformation accepts a variety of diversely substituted aromatic azides (130). Aliphatic azides on the other hand did not lead to any product formation. Tertiary and secondary isocyanides were predominantly used throughout the scope study. However, a few examples demonstrate the use of aromatic isocyanides, which also furnished tetrazoles 244 in good yields. The authors propose a catalytic cycle for the nitrene transfer step, which is in agreement with either route I or II of our proposed mechanism (Scheme 2).

In conclusion, compared to the other heteroallenes, carbodiimide synthesis *via* nitrene transfer to isocyanides and *in situ* transformation thereof holds a pivotal position. This is also reflected in the amount of base-metal catalysed processes that are already reported, of which cobalt is most often encountered. The use of simple Co^II^-salts as base-metal catalyst is more prominent than for the carbene transfer to isocyanides (Section 2.2) and multiple nitrene transfer-based cascades have been reported. However, these cascade processes are limited to solely sulfonylazides as the nitrene precursor. Mechanistic studies^84^ reveal that the isocyano-Co^II^-complex is the active species as was also observed for the Co^II^-catalysed carbene transfer to isocyanides (Scheme 17). Co^II^ seems to tolerate both aliphatic- and aromatic isocyanides. However, in transformations where both aliphatic and aromatic isocyanides were used as input, there is typically a preference for either one of the two. As for the Iron-catalysed examples both aliphatic and aromatic isocyanides seem to be both equally well accepted in the synthesis of carbodiimides, indicating that iron has the potential to allow for the use of both type of isocyanides in a cascade process. However, to date noble metal catalysis still allows for accessing a more divers set of functionalities. In addition, the scope for nitrene precursors is typically more diverse. Unfortunately, rationalizing the acceptance of either aliphatic or aromatic isocyanides in different transformations with different TM's remains troublesome with the available experimental data. In the nitrene transfer to isocyanides, Rh typically performs well for tertiary aliphatic and aromatic isocyanides, whereas Pd holds a strong preference for tertiary- and secondary aliphatic isocyanides. Although aromatic isocyanides do work for Pd, they typically perform less in the reaction. With regards to the transformations accepting aliphatic isocyanides, tertiary aliphatic isocyanides generally work best whereas primary aliphatic isocyanides and α-acidic isocyanides typically do not afford the desired product. Aromatic isocyanides standardly require sterically hinderance, such as commonly encountered 2,6-dimethylphenyl isocyanide.

With respect to the preferred mechanism for the noble-metal catalysed transformations, in the case of Pd computational data supports the formation of a Pd-isocyano species prior to nitrene formation, thereby favoring route I.^25^ However, route II cannot be fully ruled out in most reports due to a lack of experimental supporting data. The same holds for the Rh-catalysed examples.

## 4 Summary and outlook

This review highlights the current synthetic utility and mechanistic considerations of the transition metal (TM)-catalysed synthesis of heteroallenes (*i.e.* ketenes, ketenimines, isocyanates and carbodiimides) *via* carbene- and nitrene transfer reactions towards carbon monoxide and isocyanides offering a lot of opportunities to create chemical diversity.

From the reviewed literature it is evident that *in situ* manipulation of these reactive heteroallenes is preferred and provides a powerful tool in the synthesis of a variety of functional groups and organic molecules, including numerous heterocycles. Typical follow up reactions can be executed in a one-pot or even more challenging tandem process. These follow up reactions can be catalysed by the metal involved in the group transfer (relay-catalysis). Sometimes an additional transition metal is required (orthogonal relay-catalysis). Of course also a non-transition metal-catalysed follow up process is possible, indicating the huge potential for organic synthesis. The tandem processes are often multicomponent reactions ideally suited to maximize diversity for molecular library synthesis in a step-efficient manner. Different reaction types with the double bond of the heteroallenes have been exemplified such as nucleophilic addition, (formal) cycloaddition and electrocyclisation reactions. In comparison to carbon monoxide, an isocyanide provides an additional point of diversity. It can both end up in or as amino substituent of the azaheterocyclic ring itself. This certainly does not make carbon monoxide less attractive, it just creates other opportunities. After all, it is a very important building block in contemporary chemical industry and more readily accessible in comparison to isocyanides. In heteroallene synthesis it fulfils a powerful role, as both isocyanates (transfer to nitrene) and ketenes (transfer to carbenes) can be accessed with important applications, *i.e.* monomers for polyurethanes and privileged β-lactams *via* [2+2]-cycloaddition with imines, respectively.

Palladium and rhodium are to date the most widely employed transition metals to catalyse the carbene or nitrene transfer to carbon monoxide and isocyanides. Pleasingly, a handful of base-metal catalysed processes have already been developed, but these generally do not include *in situ* transformation of the generated heteroallenes. However, cobalt proves to be a promising candidate in this respect, both in the carbonylation of carbenes as well as in the imidoylation of nitrenes it has been accompanied with *in situ* further transformations. Further research in other base-metal systems, such as iron and nickel is required to replace the best in class noble-metal catalysed processes in accordance with sustainable developments. A deeper understanding of the mechanism will certainly play a pivotal role in future developments of such novel catalytic systems that allow for both efficient formation and manipulation of the linear intermediates. The use of redox-active ligands is expected to even allow to use early transition metals and are therefore appealing. Only one example is currently available and more research is certainly needed here. Its combination with *in situ* further transformations is not explored yet, providing interesting opportunities.

We set out to provide general mechanisms for heteroallene formation (route I–III) in the introduction section of this review, based on the proposals found throughout the literature. These generalised mechanistic pathways hold merit for a wide variety of TMs in both group transfer reactions to CO and isocyanides. Deviations or more detailed aspects of the general mechanisms have been discussed at the place where the specific transformations are shown in the review. The key step in the catalytic cycle is the reaction of the C1-isocyanide/carbon monoxide fragment with the metal-carbene/nitrene species, where route I and II both involve a 1,1-migratory insertion step to arrive at the corresponding η^2^-metallacycle. This is proposed to be an intermediate for low valent early transition metals. However, for late transition metals this could best be considered a transition state, relying on the computational work available. The difference between route I and II is just the order of CO/isocyanide coordination, which is specific for each catalytic system. Outer sphere addition of the C1-fragment to the metal-carbene/nitrene species (route III) is less encountered, but can be observed with complexes lacking a vacant TM coordination site to allow 1,1-migratory insertion, such as in Co^II^-porphyrin-based systems. Further detailed mechanistic studies and generation of more information with relation to the transition metal employed will be of great importance in the development of additional cascade processes, especially with base metals. Ligand design is expected to play a major role in advancing this field further.

All in all, we hope this review will induce further research into the transition metal-catalysed group transfer reaction of carbenes and nitrenes to CO and isocyanides. Research is required to expand the range of accessible organic compounds *via* further transformation of *in situ* generated heteroallenes in one-pot or tandem fashion. Acquiring a deeper mechanistic understanding will be essential to advance heteroallene synthesis with base metal catalysis.

## Conflicts of interest

There are no conflicts to declare.

## Supplementary Material
